# Specific pathogen free ten gene-edited donor pigs for xenotransplantation

**DOI:** 10.1093/procel/pwaf075

**Published:** 2025-08-25

**Authors:** Kaixiang Xu, Heng Zhao, Baoyu Jia, Jiaoxiang Wang, Nazar Ali Mohammed Ali Siddig, Muhammad Ameen Jamal, Aqiang Mao, Kai Liu, Wenjie Cheng, Chang Yang, Taiyun Wei, Feiyan Zhu, Xiaoyin Huo, Deling Jiao, Jianxiong Guo, Hongfang Zhao, Wenmin Cheng, Yuemiao Zhang, Xiangyu Zhang, Lei Jiang, Zijie Zhang, Wei Zhang, Tingbo Liang, Hong-Ye Zhao, Bei-Cheng Sun, Hong-Jiang Wei

**Affiliations:** Yunnan Province Key Laboratory for Porcine Gene Editing and Xenotransplantation, Yunnan Agricultural University, Kunming 650201, China; Yunnan Province Xenotransplantation Engineering Research Center, Yunnan Agricultural University, Kunming 650201, China; College of Veterinary Medicine, Yunnan Agricultural University, Kunming 650201, China; Yunnan Province Key Laboratory for Porcine Gene Editing and Xenotransplantation, Yunnan Agricultural University, Kunming 650201, China; Yunnan Province Xenotransplantation Engineering Research Center, Yunnan Agricultural University, Kunming 650201, China; College of Veterinary Medicine, Yunnan Agricultural University, Kunming 650201, China; Yunnan Province Key Laboratory for Porcine Gene Editing and Xenotransplantation, Yunnan Agricultural University, Kunming 650201, China; Yunnan Province Xenotransplantation Engineering Research Center, Yunnan Agricultural University, Kunming 650201, China; College of Veterinary Medicine, Yunnan Agricultural University, Kunming 650201, China; Yunnan Province Key Laboratory for Porcine Gene Editing and Xenotransplantation, Yunnan Agricultural University, Kunming 650201, China; Yunnan Province Xenotransplantation Engineering Research Center, Yunnan Agricultural University, Kunming 650201, China; Yunnan Province Key Laboratory for Porcine Gene Editing and Xenotransplantation, Yunnan Agricultural University, Kunming 650201, China; Yunnan Province Xenotransplantation Engineering Research Center, Yunnan Agricultural University, Kunming 650201, China; Faculty of Animal Science and Technology, Yunnan Agricultural University, Kunming 650201, China; Yunnan Province Key Laboratory for Porcine Gene Editing and Xenotransplantation, Yunnan Agricultural University, Kunming 650201, China; Yunnan Province Xenotransplantation Engineering Research Center, Yunnan Agricultural University, Kunming 650201, China; Yunnan Province Key Laboratory for Porcine Gene Editing and Xenotransplantation, Yunnan Agricultural University, Kunming 650201, China; Yunnan Province Xenotransplantation Engineering Research Center, Yunnan Agricultural University, Kunming 650201, China; College of Veterinary Medicine, Yunnan Agricultural University, Kunming 650201, China; Yunnan Province Key Laboratory for Porcine Gene Editing and Xenotransplantation, Yunnan Agricultural University, Kunming 650201, China; Yunnan Province Xenotransplantation Engineering Research Center, Yunnan Agricultural University, Kunming 650201, China; Faculty of Animal Science and Technology, Yunnan Agricultural University, Kunming 650201, China; Yunnan Province Key Laboratory for Porcine Gene Editing and Xenotransplantation, Yunnan Agricultural University, Kunming 650201, China; Yunnan Province Xenotransplantation Engineering Research Center, Yunnan Agricultural University, Kunming 650201, China; Faculty of Animal Science and Technology, Yunnan Agricultural University, Kunming 650201, China; Yunnan Province Key Laboratory for Porcine Gene Editing and Xenotransplantation, Yunnan Agricultural University, Kunming 650201, China; Yunnan Province Xenotransplantation Engineering Research Center, Yunnan Agricultural University, Kunming 650201, China; Faculty of Animal Science and Technology, Yunnan Agricultural University, Kunming 650201, China; Yunnan Province Key Laboratory for Porcine Gene Editing and Xenotransplantation, Yunnan Agricultural University, Kunming 650201, China; Yunnan Province Xenotransplantation Engineering Research Center, Yunnan Agricultural University, Kunming 650201, China; Yunnan Province Key Laboratory for Porcine Gene Editing and Xenotransplantation, Yunnan Agricultural University, Kunming 650201, China; Yunnan Province Xenotransplantation Engineering Research Center, Yunnan Agricultural University, Kunming 650201, China; Faculty of Animal Science and Technology, Yunnan Agricultural University, Kunming 650201, China; Yunnan Province Key Laboratory for Porcine Gene Editing and Xenotransplantation, Yunnan Agricultural University, Kunming 650201, China; Yunnan Province Xenotransplantation Engineering Research Center, Yunnan Agricultural University, Kunming 650201, China; College of Veterinary Medicine, Yunnan Agricultural University, Kunming 650201, China; Yunnan Province Key Laboratory for Porcine Gene Editing and Xenotransplantation, Yunnan Agricultural University, Kunming 650201, China; Yunnan Province Xenotransplantation Engineering Research Center, Yunnan Agricultural University, Kunming 650201, China; College of Veterinary Medicine, Yunnan Agricultural University, Kunming 650201, China; Yunnan Province Key Laboratory for Porcine Gene Editing and Xenotransplantation, Yunnan Agricultural University, Kunming 650201, China; Yunnan Province Xenotransplantation Engineering Research Center, Yunnan Agricultural University, Kunming 650201, China; Yunnan Province Key Laboratory for Porcine Gene Editing and Xenotransplantation, Yunnan Agricultural University, Kunming 650201, China; Yunnan Province Xenotransplantation Engineering Research Center, Yunnan Agricultural University, Kunming 650201, China; Yunnan Province Key Laboratory for Porcine Gene Editing and Xenotransplantation, Yunnan Agricultural University, Kunming 650201, China; Yunnan Province Xenotransplantation Engineering Research Center, Yunnan Agricultural University, Kunming 650201, China; Faculty of Animal Science and Technology, Yunnan Agricultural University, Kunming 650201, China; Renal Division, Key Laboratory of Renal Disease, Peking University First Hospital, Peking University Institute of Nephrology, Beijing 100034, China; Renal Division, Key Laboratory of Renal Disease, Peking University First Hospital, Peking University Institute of Nephrology, Beijing 100034, China; Renal Division, Key Laboratory of Renal Disease, Peking University First Hospital, Peking University Institute of Nephrology, Beijing 100034, China; Bio-X Center for Interdisciplinary Innovation, Yunnan University, Kunming 650091, China; The First Affiliated Hospital, Zhejiang University School of Medicine, Hangzhou 311103, China; The First Affiliated Hospital, Zhejiang University School of Medicine, Hangzhou 311103, China; Yunnan Province Key Laboratory for Porcine Gene Editing and Xenotransplantation, Yunnan Agricultural University, Kunming 650201, China; Yunnan Province Xenotransplantation Engineering Research Center, Yunnan Agricultural University, Kunming 650201, China; College of Veterinary Medicine, Yunnan Agricultural University, Kunming 650201, China; The First Affiliation Hospital of Anhui Medical University, Hefei 230022, China; Yunnan Province Key Laboratory for Porcine Gene Editing and Xenotransplantation, Yunnan Agricultural University, Kunming 650201, China; Yunnan Province Xenotransplantation Engineering Research Center, Yunnan Agricultural University, Kunming 650201, China; College of Veterinary Medicine, Yunnan Agricultural University, Kunming 650201, China

**Keywords:** donor pig, gene editing, immune rejection, pathogenic microorganisms, xenotransplantation

## Abstract

Xenotransplantation has entered the clinical phase in an effort to address the global organ shortage. However, recent clinical studies have revealed that current xenografts from gene-edited (GE) pigs still pose a risk of immune rejection and biosafety concerns. In this study, we successfully produced a large batch of 582 GE cloned (GEC) pigs with 10-(GTKO/CMAHKO/β4GalNT2KO/hCD46/ hCD55/hCD59/hTBM/hCD39/hEPCR/hCD47) gene edits via gene editing and somatic cell cloning technologies, and successfully obtained the F1 generation. Phenotypic characterization of 10-GEC pigs revealed the deletion of three xenoantigens and the expression of seven human transgenes across various tissues. Digital droplet polymerase chain reaction and whole-genome sequencing revealed two copies of hCD46/hCD55/hCD59/hTBM/hCD39 and one copy of hEPCR/hCD47 in the pig genome with minimal off-target effects or damage to the porcine functional genes. The validation results showed that 10-GEC pigs could effectively inhibit attacks from human antibodies, complement and macrophages on porcine endothelial cells, and alleviated coagulation abnormalities between pigs and humans. Large-scale screening of pathogens revealed no evidence of 47 pathogens, including cytomegalovirus, in our 10-GEC pigs. Kidney, heart and liver xenografts from these 10-GEC pigs were transplanted into nonhuman primates (NHPs), which worked normally without hyperacute rejection (HAR). Among NHPs, the heart and liver orthotopic transplant recipients survived for 3 and 4 days, respectively, while the two kidney transplant recipients survived for 23 and 16 days, respectively. Pathological analysis showed interstitial hemorrhage and fibrosis, cellular hyperplasia with minor antibodies and complement deposition, but significantly reduced infiltration of CD68^+^ macrophages in 10-GEC pig kidney xenografts. In summary, we successfully produced specific pathogen-free 10-GEC donor pigs that resulted in effective mitigation of immune rejection upon multiorgan transplantation to NHPs.

## Introduction

Xenotransplantation is on the verge of entering the clinical phase to save the lives of patients with end-stage organ failure. In 2022, the University of Maryland Medical Center conducted the world’s first heart transplant from a 10-GE pig to a 57-year-old patient, which sustained the life for up to 60 days without significant immune rejection (Griffith et al., 2022). A year later, the same medical center performed the world’s second heart transplant from the same 10-GE pig to a 58-year-old patient who survived for 40 days without significant immune rejection (Griffith et al., 2025). Similarly, in 2024, the world’s first kidney xenograft from an 11-GE pig was transplanted into a 62-year-old patient at Massachusetts General Hospital, who survived for 52 days (Kawai et al., 2025). Subsequently, NYU Langone Medical Center performed the world’s second kidney transplant from a 1-GE pig to a 54-year-old patient who survived for 47 days (Gordon et al., 2025). At the end of 2024, the same center performed the world’s third kidney transplant from a 10-GE pig to a 53-year-old live patient, which sustained normal function for 130 days. Recently, Massachusetts General Hospital also performed xenotransplantation of the world’s fourth kidney from 11-GE pigs to living patients, which is functioning well, hereby indicating the great potential of GE pigs to address the global organ shortage.

Pig-to-human xenotransplantation still faces many obstacles, such as immune incompatibility, including antibody-mediated rejection (AMR), cell-mediated rejection, coagulation disorders and blood-mediated transient inflammatory responses, which have been effectively mitigated by gene editing (Puga Yung et al., 2017). Nonetheless, the development of immune rejection further involves the activation of multiple signaling pathways and the participation of multiple immune regulatory molecules, thus acquiring extensive gene editing in donor pigs. However, it is still unclear how many genes should be edited and which genetic combinations are suitable for which organ transplantation.

Knockout (KO) of the porcine GGTA1 gene (GTKO) was performed to eliminate HAR mediated by binding of human natural antibodies to the αGal xenoantigen (Lai et al., 2002). On this basis, it was further confirmed that the KO of two other xenoantigens (CMAH and β4GalNT2) exhibited reduced human IgM and IgG binding, demonstrating further reduction in AMR (Estrada et al., 2015). Thus, triple KO (TKO, GTKO/CMAHKO/β4GalNT2KO) donor pigs are currently considered as the basic configuration for xenotransplantation (Hara et al., 2023).

Human complement regulatory proteins (hCRPs) can effectively inhibit most of the AMR, as evidenced by *in-vitro* and *in-vivo* studies that the expression of hCD46 or hCD55 significantly reduced cytotoxicity in porcine cells exposed to human serum (Lee et al., 2016; Miyagawa et al., 1994), and prolonged the survival of kidney and heart xenografts after transplantation to NHPs (Schuurman et al., 2002). Similarly, the expression of hCRP in combination with GTKO reduced early graft rejection (Azimzadeh et al., 2015), and kidney xenograft from GTKO/hCD55 pigs survived up to 499 days into NHPs (Kim et al., 2019). Nevertheless, the recent clinical trials of heart and kidney xenografts from 10-GE and 11-GE donor pigs, respectively, revealed retained complement deposition, although both of hCRPs (hCD46/hCD55) were overexpressed (Griffith et al., 2025; Kawai et al., 2025). These findings suggest that overexpression of two hCRPs might be insufficient to completely inhibit the cytotoxic effect of the complement system, hereby necessitating the further genetic modification for the inhibition of complement system activation. Therefore, in this study, we propose the KO of 3 xenoantigens (GTKO/CMAHKO/β4GalNT2KO) and the simultaneous expression of three hCRPs (hCD46, hCD55 and hCD59) to further alleviate the immune injury caused by complement system activation.

The development of thrombotic microangiopathy (TMA) due to coagulation system incompatibilities between pig and human often leads to xenograft failure (Houser et al., 2004; Shimizu et al., 2005, 2008). Nevertheless, the expression of human coagulation regulatory proteins such as thrombomodulin (TBM) and vascular endothelial cell protein C receptor (EPCR) can act synergistically, and increase the efficiency of protein C activation, which in turn inhibits graft microcirculatory thrombus formation, thereby extending survival (Iwase et al., 2015). In addition, CD39 molecules hydrolyze the ATP and ADP, which prevents excessive inflammatory responses and tissue damage, thereby inhibiting the thrombus formation due to platelet aggregation (Kanthi et al., 2014). Therefore, we also propose the simultaneous expression of these three coagulation regulatory proteins (hTBM, hEPCR and hCD39) to reduce TMA.

Macrophage activation is also critical for xenograft survival, as evidenced by pig-to-NHPs and pig-to-brain-dead human xenotransplantation that a large number of CD68-positive macrophages infiltrated in pig kidney xenografts (Wang et al., 2024, 2025; Yang et al., 2024), which directly or indirectly led to loss of kidney xenograft function. Moreover, a large number of sedentary macrophages (Kupffer cells) are also present in the sinusoidal lining of porcine livers, and these cells continuously phagocytose human platelets, leading to a dramatic decrease in the platelet count. However, *in-vitro* and *in-vivo* experiments revealed that CD47 expression can inhibit macrophage activation and inflammatory factors production (Jung et al., 2017), thereby prolonging the survival of the kidney xenograft (Takeuchi et al., 2021). Therefore, we also consider the expression of human CD47 to prolong xenograft survival by inhibiting macrophage phagocytosis.

In recent years, zoonotic pathogenic microorganisms have been found to be critical for the survival of xenografts in both NHP and human recipients (Denner et al., 2020). For example, in the world’s first pig-to-live human patient cardiac xenotransplantation, reactivation and replication of porcine cytomegalovirus (PCMV/PRV) was a significant finding, demonstrating the potential to trigger a devastating inflammatory response that could lead to patient death (Mohiuddin et al., 2023). Moreover, patients undergoing xenotransplantation underwent stronger immunosuppressive treatment than allotransplantation, thereby increasing the likelihood of transmission of porcine pathogenic microorganisms. The World Health Organization (WHO) has clearly announced the serious biosafety concerns with 55 species of pathogenic microorganisms being potentially transmissible to humans (Fishman et al., 2012). Hence, the development of multi-GE pigs free from zoonotic pathogenic microorganisms could potentiate the clinical application of GE donor (GED) pigs for xenotransplantation. Therefore, in this study, we incorporated the first-time large-scale screening of pathogenic microorganisms for the development of GED pigs for the xenotransplantation field.

Our team has conducted xenotransplantation research for almost two decades and successfully inactivated the porcine endogenous retroviruses (PERVs) to prevent cross-species viral transmission (Niu et al., 2017), and produced 1-GE (GGTA1) (Cheng et al., 2016), 3-GE (GTKO, hCD55, hCD59) (Liu et al., 2018; Zhao et al., 2020), (GGTA1, B2M, CIITA) (Xu et al., 2022), 4-GE (GGTA1, hCD55, hTBM, hCD39) (Yang et al., 2024), 8-GE (GGTA1, CMAH, β4GalNT2, hCD46, hCD55, hCD59, hTBM, hCD39) (Wang et al., 2025), and 13-GE (PERV-pol, GGTA1, CMAH, β4GalNT2, hCD46, hCD55, hCD59, hB2M, HLA-E, hCD47, hTHBD, hTFPI and hCD39) pigs (Yue et al., 2021).

In the present study, we used gene editing and somatic cell nuclear transfer (SCNT) technologies to produce 10-(GTKO/CMAHKO/β4GalNT2KO/hCD46/ hCD55/hCD59/hTBM/hCD39/hEPCR/hCD47) GEC *Diannan* miniature pigs. For the first time, we conducted a large-scale screening of pathogenic microorganisms in these GED pigs and performed pig-to-NHPs xenotransplantation to provide safe and effective donor pigs for the clinical application of xenotransplantation.

## Results

### Generation of 10-GEC pigs

To produce 10-(GTKO/CMAHKO/β4GalNT2KO/hCD46/hCD55/hCD59/hTBM/hCD39/hEPCR/hCD47) GEC donor pigs, we utilized the CRISPR/Cas9 system, the PiggyBac (PB) transposon system combined with somatic cell cloning technology, and first co-transfected the previously constructed TKO (GTKO/CMAHKO/β4GalNT2KO) vectors, with 5 transgenes (hCD46/hCD55/hCD59/hTBM/hCD39) vectors, and PB transposase vectors into the fetal fibroblasts of male *Diannan* miniature pigs. Fibroblasts were screened for Puro resistance and genotyped to obtain 8-GE cell colonies with TKO and integration of 5 transgenes, which were then used as donors for the first round of SCNT and successfully obtained 8-GEC fetuses. After identification, the 8-GEC fetuses were selected for recloning to obtain 8-GEC pigs. Considering the 8-GEC pigs with high survival rates, the corresponding fetal cell lines were selected for the second editing. The PB vector of 2 transgenes (hEPCR and hCD47) was transfected into 8-GE fibroblasts, which were screened for HygR resistance and cultured in limited dilution to obtain 10-GE single cell colonies. These cell colonies were then used as a donor cell for SCNT, and 10-GEC fetuses were obtained and genotyped, and used as a donor for recloning to obtain 10-GEC pigs (Fig. S1A). For TKO, multiple sgRNAs targeting the exons 3, 4 and 2 of GGTA1, CMAH and β4GalNT2 genes, respectively, were designed (Fig. S1B). The expression of transgenes was driven by the human EF-1α promoter (hCD46, hCD55 and hCD59), the human endothelial cell-specific promoter ICAM2 promoter (hTBM and hCD39), and the CAG promoter (hEPCR and hCD47) (Fig. S1C).

**Figure 1.**
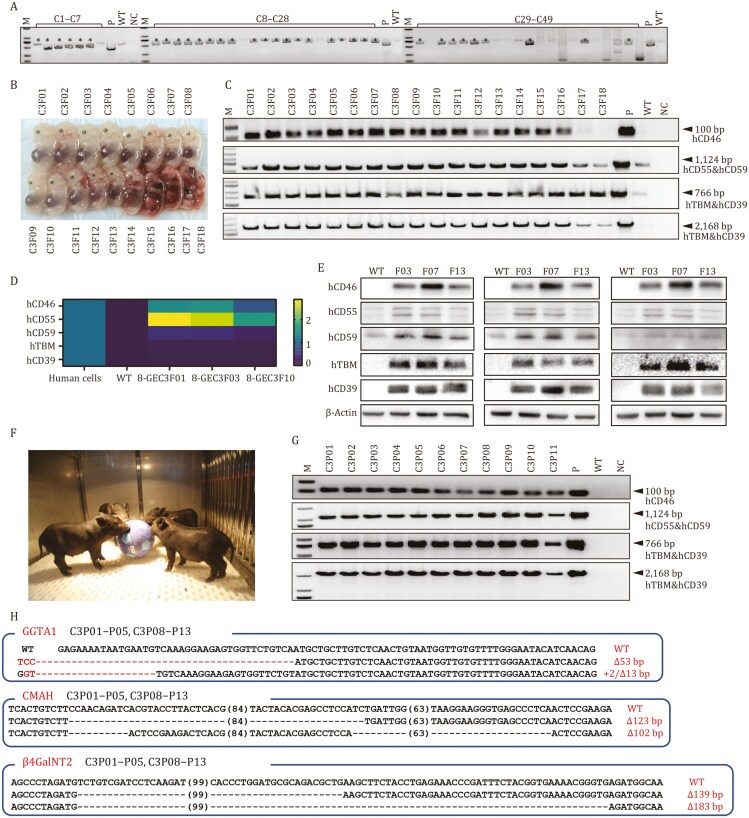
**Construction and identification of 8-GEC pigs with the KO of 3 xenoantigens and overexpression of 5 transgenes.** (A) PCR identification of cell colonies. The colonies with an asterisk (*) indicate successful integration of 5 transgenes (hCD46, hCD55, hCD59, hTBM and hCD39) into pig genome. (B) Photo of cloned fetuses obtained after 33-day pregnancy. (C‒E) Identification of cloned fetuses for successful integration of 5 transgenes by PCR (C), qPCR (D) and Western blot (triplicates) (E). (F) Photo of 8-GEC pigs. (G) Identification of integration of 5 transgenes in 8-GEC pigs by PCR. (H) Genotypes of the targeting regions of GGTA1, CMAH and β4GalNT2 genes in 8-GEC pigs determined by Sanger sequencing. Abbreviations: M, marker; P, plasmid; NC, negative control; WT, wild type; GEC, gene-edited cloned.

After electroporation and drug selection, a total of 49 cell colonies were obtained. First, these colonies were identified by PCR, out of which 34 carried the 5 transgenes (hCD46, hCD55, hCD59, hTBM and hCD39, Fig. 1A). Then a total of 32 colonies were randomly selected for Sanger sequencing and the results revealed that only the colony C3# might be biallelic TKO carrying transgenes (Tables S3 and S4), thus this colony was used as a donor for SCNT and eighteen 33-day-old cloned fetuses were obtained (Fig. 1B). Among them, 13 live fetuses (C3F01 to C3F13) successfully carried the 5 transgenes (Fig. 1C), and were biallelic TKO (Fig. S2; Table S5). The qPCR results showed robust mRNA expression of hCD46, hCD55 and hCD59 transgenes, whereas relatively weak expression of hTBM and hCD39 transgenes in 8-GEC fetal fibroblasts (Fig. 1D), which may have been due to the endothelial cell-specific expression of hTBM and hCD39 transgenes. Notably, the Western blot (WB) results confirmed the expression of five corresponding proteins in 8-GEC fetal tissues (C3F03, C3F07 and C3F13) (Fig. 1E). Therefore, these three 8-GEC fetal fibroblasts were used for recloning, and reconstructed embryos were transferred into eight surrogate sows, giving birth to 38 piglets, of which 18 survived (Table S6). Their genotypes were consistent with those of 8-GEC fetal fibroblasts, i.e., biallelic TKO (Table S7). Additionally, we also cloned a batch of piglets (*n* = 13) directly by using the cell colony C3# as a donor cell (Fig. 1F; Table S8), which exhibited normal reproduction. Their genomes successfully carried 5 transgenes (Fig. 1G), with biallelic TKO (Fig. 1H; Table S9).

**Figure 2.**
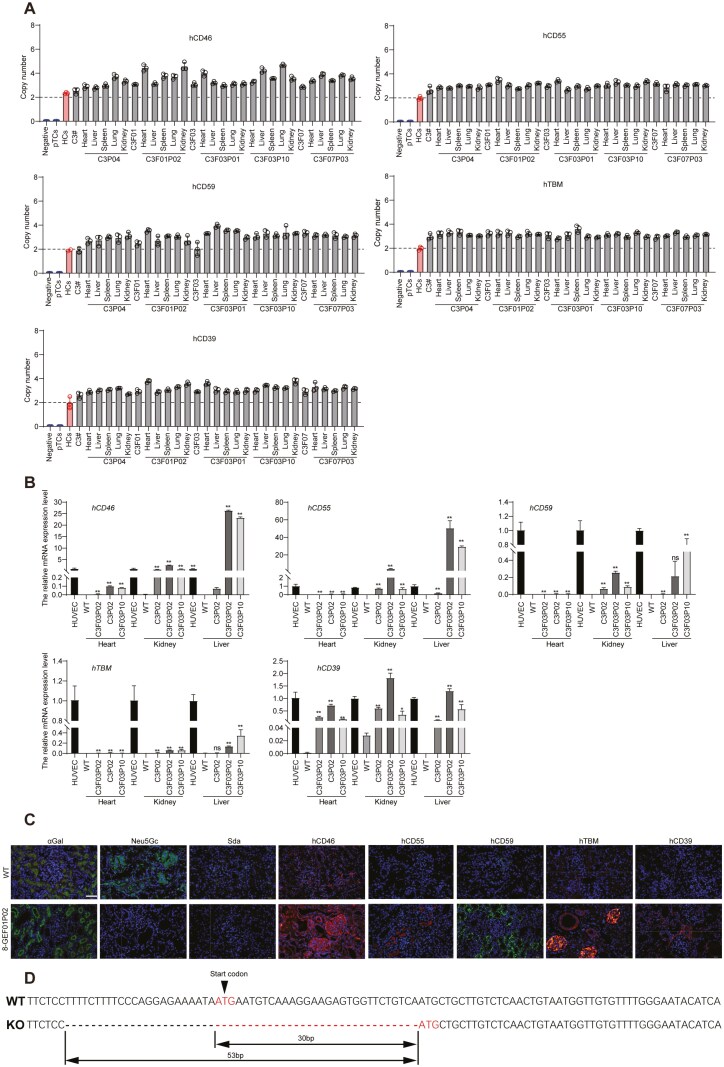
**Copy number and phenotype of 8-GEC pigs.** (A) Copy numbers of 5 transgenes in donor cell lines, 8-GEC fetuses and piglets. (B) mRNA expression levels of 5 transgenes in heart, kidney and liver tissues of 8-GEC pigs. (C) The expression of three xenoantigens and five transgenes in 8-GEC pig kidney by immunofluorescence. Scale bar = 50 µm. (D) Analysis of deleted fragments at the targeting region of GGTA1 gene in 8-GEC pigs. *P < 0.05, **P < 0.01, ns, no significance. Abbreviations: pTCs, pre-transfected cells; HCs, human cells; Negative, water; C3#, transgene positive cell colony; HUVEC, human umbilical vein endothelial cell; WT, wild type, GEC, gene-edited cloned.

We next performed copy number analysis of 5 transgenes in the heart, liver, spleen, lung and kidney tissues of 8-GEC pigs, and the results revealed that these tissues contained 3 copies of each transgene (Fig. 2A). The qPCR analysis showed the mRNA expression of 5 transgenes in heart, kidney and liver tissues of 8-GEC pigs (Fig. 2B). Moreover, the immunofluorescence staining showed that except αGal antigen, the other 2 xenoantigens (Neu5Gc and Sda) were deficient and 5 transgenes were expressed in the kidneys of 8-GEC pigs (Fig. 2C). We carefully analyzed the genotype of the GGTA1 gene in the 8-GEC pigs and found that despite the homozygous mutation at the exon 3 of GGTA1 gene, an allele with a deletion of 53 bp, exactly a deletion of 30 bp when calculated from the start codon ATG, presenting a multiple of 3 (Fig. 2D), which did not result in a frame-shift mutation of GGTA1 gene, rather caused the GGTA1 gene to form a truncated protein. This truncated protein could likely catalyze the synthesis of αGal antigen, which ultimately led to incomplete KO of GGTA1 gene. Therefore, in the process of generating 10-GEC pigs, we redesigned the targeting vector at the exon 8 of GGTA1 gene (Fig. S1B).

**Figure 3.**
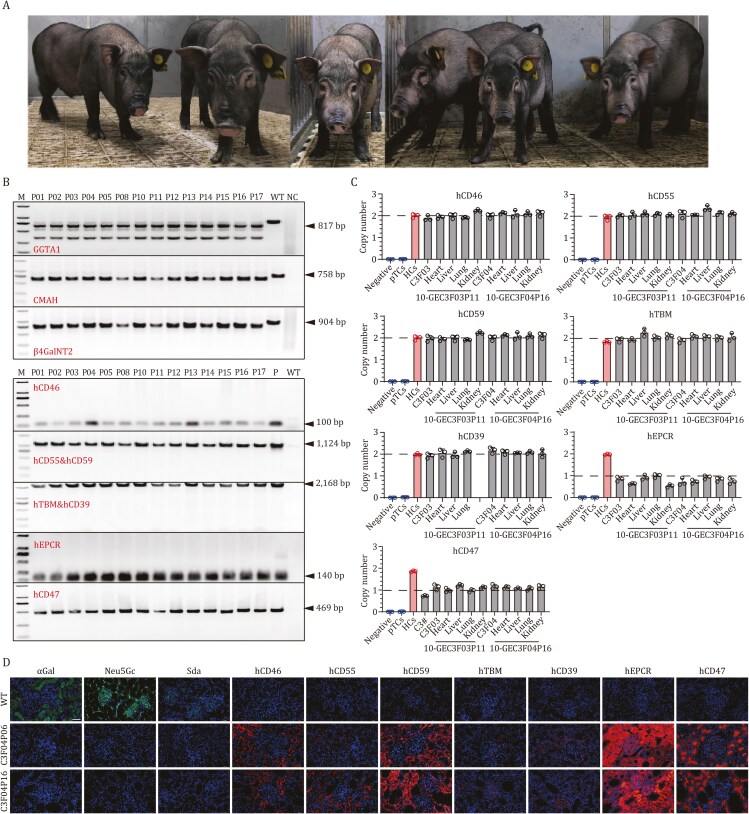
**Identification of 10-GEC pigs.** (A) Photo of 10-GEC pigs. (B) Identification of 10-GEC pigs for TKO and integration of 7 transgenes by PCR. (C) Copy number of 7 transgenes in 10-GEC pigs. (D) Expression of 3 xenoantigens and 7 transgenes in 10-GEC pig kidneys by immunofluorescence. Scale bar = 50 µm. Abbreviations: M, marker; WT, wild type; NC, negative control; pTCs, pre-transfected cells; HCs, human cells; Negative, water; C3#, transgene positive cell colony; GEC, gene-edited cloned.

On the basis of 8-GEC fetus (C3F01), we performed a second transfection and obtained 58 cell colonies after HygR resistance screening and limited dilution culture. First, PCR identification of the target region at the exon 8 of GGTA1 gene showed that 16 colonies (C1#, C3#, C4#, C6#, C7#, C12#, C15#, C16#, C19#, C22#, C23#, C28#, C31#, C36#, C54# and C58#) presented obvious split bands in the target region (Fig. S3A), which were predicted to be the cell colonies with large fragment deletions in GGTA1 gene. Sanger sequencing showed that all these cell colonies contained biallelic mutations of more than 100 bp (Table S10). These 16 cell colonies were subsequently identified by PCR, and the results showed that 15 of them had hEPCR and hCD47 gene insertions (Fig. S3B). Next, we selected one colony (C3#) with a large deletion mutation at the exon 8 of GGTA1 gene as a donor for SCNT, and five 33-day-old live fetuses were obtained (Fig. S3C). The genotyping results showed that all of the GEC fetuses were biallelic TKO (Table S11), along with the integration of 7 transgenes (Fig. S3D and S3E). Moreover, the qPCR results showed the expression of 7 transgenes at mRNA levels in the GEC fetuses (F01–F03, Fig. S3F), hence these GEC fetuses were recognized as 10 gene edits and used as a donor cell for recloning, and reconstructed embryos were transferred into surrogate sows, of which 118 delivered a total of 582 GEC piglets with an average of 4.9 cloned piglets per surrogate sow (Table S12; Fig. 3A). Genotyping results showed that these 10-GEC pigs were genotypically consistent with the donor fetal fibroblast, i.e., biallelic TKO with the integration of 7 transgenes (Fig. 3B; Table S13). Genome-wide sequencing for off-target analysis showed that only 1 intergenic region (RRAGD and ANKRD6 genes) was off-target in the 10-GEC pigs in the presence of 4 base mismatches (Table S14). Droplet digital polymerase chain reaction (ddPCR) results showed that the copy number of five transgenes (hCD46, hCD55, hCD59, hTBM and hCD39) decreased from 3 copies in 8-GEC pigs to 2 copies in 10-GEC pigs, whereas the copy number of the hEPCR and hCD47 genes was 1 in 10-GEC pigs (Fig. 3C). The reduction in copy number may be attributed to the PiggyBac transposon system, which integrates genes via a “cut-and-paste” mechanism and may have excised previously integrated copies during the second round of editing (Li et al., 2013). Genome-wide transgene insertion analysis showed that the 3 transgene copies (2 for five transgenes and 1 for two transgenes) were inserted into the intergenic regions or introns of chromosomes 5, 6 and 13, respectively, and did not cause damage to the functional genes of the pigs (Table S15). The qPCR analysis showed the mRNA expression of all transgenes in heart, kidney and liver tissues of 10-GEC pigs (Fig. S4A). Immunofluorescence staining of kidney tissues from 10-GEC pigs showed that 3 xenoantigens were deficient, and 7 transgenes were expressed, with significantly prominent expression of 2 transgenes (hEPCR and hCD47, Fig. 3D). To obtain the F1 generation, 10-GEC male pigs (*n* = 4) were back-crossed with 8-GEC sows (*n* = 4), giving birth to 24 F1 generation piglets (Fig. S4B; Table S16), of which some successfully carried TKO biallelic mutations with integration of 7 transgenes (Tables S17 and S18). These results demonstrated that our 10-GEC pigs can reproduce offspring, laying the foundation for the expansion and breeding of xenotransplantation donor pig populations.

Next, we collected nose/mouth/throat swabs, feces, skin flake, skin, serum and whole blood samples from 6- to 9-month-old 10-GEC pigs housed at specific pathogen free (SPF)-grade facilities, and performed large-scale screening for a total of 48 pathogenic microorganisms including 13 bacterial species (*Brucella*, *Leptospira*, *Serpulina hyodysenteriae*, *Mycobacterium bovis*, *Mycobacterium tuberculosis*, *Mycobacterium avium-intracellulare complex*, *Mycoplasma hyopneumoniae*, *Salmonella*, *Shigella species*, *Bordetella bronchiseptica*, *Pasteurella multocida*, *swine Actinobacillus pleuropneumoniae* and *Streptococcus suis*), 3 fungal species (Pathogenic dermal fungi, *Cryptococcus neoformans* and *Histoplasma capsulatum*), and 22 viruses (Porcine adenovirus, Porcine encephalomyocarditis virus, Porcine influenza virus H3N2, Human influenza virus, Porcine influenza virus H1N1, Porcine cytomegalovirus, Herpesvirus, Porcine reproductive and respiratory syndrome virus, Porcine parvovirus, Rotavirus, Pseudorabies virus, Rabies virus, Foot-and-mouth disease virus, Classical swine fever virus, Japanese encephalitis virus, Porcine circovirus 1, Porcine circovirus 2, Porcine circovirus 3, Porcine transmissible gastroenteritis virus, Swine vesicular disease virus, Influenza A virus and Influenza B virus), and 10 parasitic species (Ectoparasite, *Fasciolopsis buski*, *Ascaris suum*, *Echinococcus granulosus*, *Strongyloides ransomi*, *Isospora, Eimeria*, *Toxoplasma gondii*, *Trichinella spiralis* and *Neospora caninum*). The results showed that our 10-GEC pigs were negative for a total of 47 pathogenic microorganisms, including porcine cytomegalovirus, with the exception of *Streptococcus suis* (Table S19).

### Functional validation of xenografts from 10-GEC pigs

**Figure 4.**
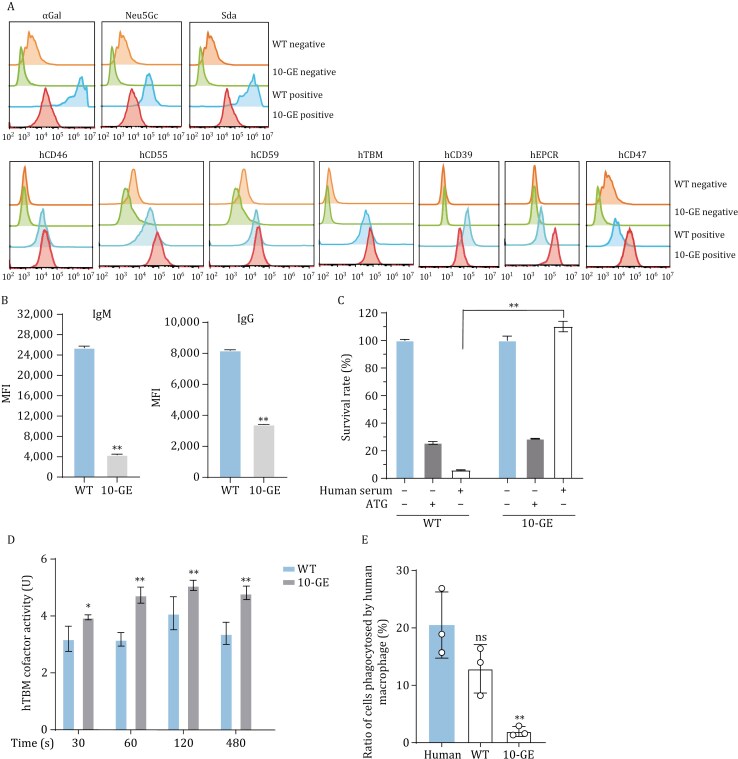
**Identification and functional verification of 10-GEC pigs.** (A) The expression of 3 xenoantigens and 7 transgenes in 10-GEC pig aortic endothelial cells (PAECs) by flow cytometry. (B) Levels of human IgG and IgM binding to 10-GE porcine PAECs. (C) Survival rate of 10-GEC porcine PBMCs. ATG, anti-thymocyte globulin as a positive control. (D) Anti-coagulation activity of 10-GEC pigs’ PAECs. (E) Phagocytosis of 10-GEC PAECs by human macrophages. *P < 0.05, **P < 0.01, ns, no significance. GEC, gene-edited cloned.

Flow cytometric analysis revealed that the expression of 3 xenoantigens was absent, and the expression of 6 transgenes (hCD46, hCD55, hCD59, hTBM, hEPCR and hCD47) except hCD39 were detectable in 10-GEC pigs (Fig. 4A). An antigen-antibody binding assay revealed that the binding ability of human IgG and IgM to 10-GEC pig’s aortic endothelial cells (PAECs) was significantly reduced than that of WT control (Fig. 4B). Complement-dependent cytotoxicity (CDC) assay showed that the survival rate of 10-GEC pig’s peripheral blood mononuclear cells (PBMCs) was higher than that of WT control (Fig. 4C). Coagulation assay revealed that the PAECs of 10-GEC pigs exhibited the significant strong binding ability to thrombin as compared to WT PAECs (Fig. 4D). To confirm that hCD47 effectively prevented phagocytosis of porcine cells by human macrophages, porcine PAECs were incubated with Phorbol 12-Myristate 13-Acetate (PMA)-induced human THP-1 cells, and the results showed that 10-GEC pig’s PAECs were significantly less phagocytosed by human macrophages (Fig. 4E). In conclusion, 10-GEC pigs could effectively resist immune rejection caused by antigen-antibody binding, complement system activation, coagulation dysregulation and macrophage activation *in vitro*.

### Organ transplantation from 10-GEC xenotransplantation donor pigs to NHPs

In order to evaluate the effectiveness of our 10-GEC pigs as a donor for xenotransplantation, we performed the transplantation of multiple organs, i.e., kidney, heart and liver from these 10-GEC pigs to Tibetan macaques. We first collected serum samples from 10 Tibetan macaques and isolated PBMCs from 10-GEC pigs (10-GEC3F02P02 and 10-GEC3F04P12). IgM and IgG antibody binding assay, along with CDC assays, were performed. The results of antigen-antibody binding assay showed that the mean fluorescence intensity (MFI) values for IgM and IgG binding from all Tibetan macaques to PBMCs from 10-GEC pigs were below 2,000, while of CDC assay showed that four macaques exhibited a cell survival rate of > 50% (Fig. S5A), hence those four macaques were selected as recipients for xenotransplantation (Table S20). Two days before surgery, an immunosuppression regimen was adopted as described in our previous study (Wang et al., 2025). On the day of surgery, we simultaneously harvested 1 kidney, 1 heart and 1 liver from one 10-GEC pig (10-GEC3F02P02), and transplanted them into 3 Tibetan macaques after *in vitro* perfusion, and 1 kidney from another 10-GEC pig (10-GEC3F04P12) was transplanted into fourth Tibetan macaque after *in vitro* perfusion. Soon after completion of surgery, the xenograft started working normally, i.e., kidney produced urine, heart started beating, and the liver secreted bile, without HAR (Figs. S5B, S6A, and S6B). The heart and liver transplant recipients were sustained under an anesthesia ventilator on the operating table for clinical observation and care after surgery. On the 3rd postoperative day, unexpected interruption of the oxygen supply led to cardiac arrest in the heart transplant recipients, which resulted in unsuccessful resuscitation and unfortunate death in the liver transplant recipients on the 4th postoperative day. We performed H&E staining of the liver tissues, and the results showed partial vascular endothelial breakdown on postoperative day 1 (Fig. S6C); while on postoperative day 4, necrotic disintegration of hepatocytes was observed (Fig. S6D and S6E); however, immunohistochemistry (IHC) revealed no detection of complement C4d deposition on postoperative day 1 (Fig. S6F). Next, we summarized and analyzed mainly the 2 remaining kidney transplant recipients in subsequent experiments.

**Figure 5.**
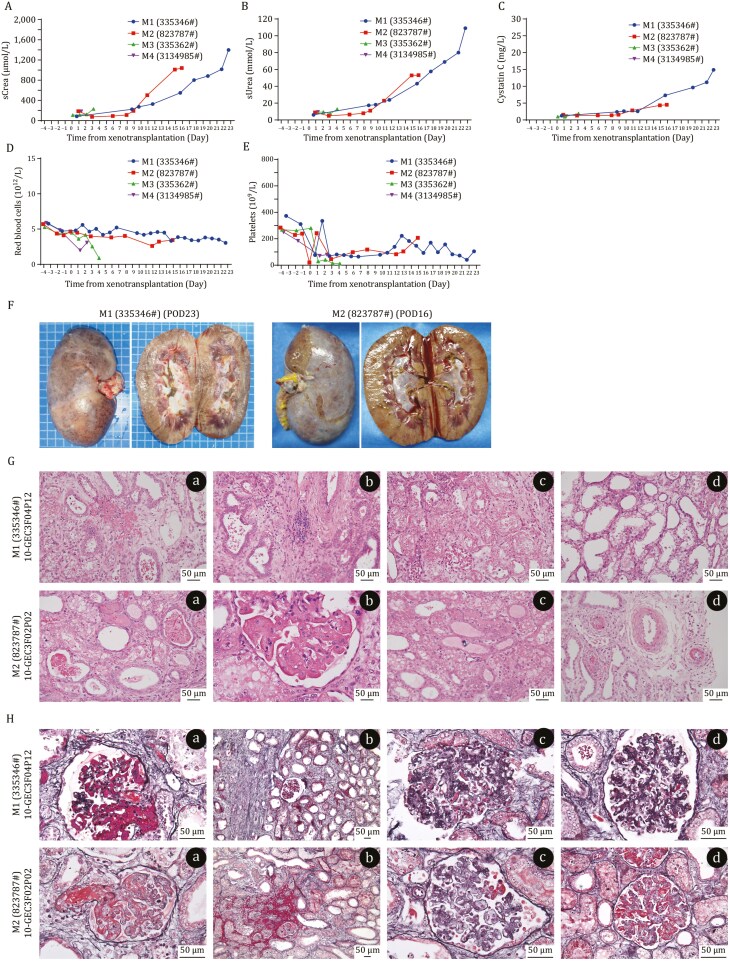
**Renal function, erythrocytes indices of recipient macaques and pathological analysis of kidney xenografts from 10-GEC pigs.** (A‒C) Levels of (A) creatinine, (B) urea and (C) cystatin C in the serum of recipients. (D and E) Number of red blood cells (D) and platelets (E). (F) Necropsy of two kidney xenograft of postoperative day 23 and 16. POD, postoperative day. (G) H&E staining of two kidney xenografts. Scale bar = 50 µm. M1 recipient transplanted kidney manifested interstitial hemorrhage (a), inflammatory infiltration (b), tubular infarction (c) and vacuolar degeneration (d). M2 recipient transplanted kidney showed erythrocyte stasis (a), neutrophil infiltration (b), tubular calcium salt deposition (c) and small arteriolar thrombosis (d). (H) PASM and Masson staining of two kidney xenografts. Scale bar = 50 µm. M1 recipient transplanted kidney manifested small arteriolar-thrombi (a), interstitial fibrosis (b), cellular hyperplasia (c) and tethered basement membrane hyperplasia (d). M2 recipient transplanted kidney showed lesions such as thrombus formation at the entrance of glomerular afferent arterioles (a), interstitial hemorrhage (b), glomerular cell hyperplasia (c) and auricular (ear-like) deformation of the glomerular tuft (d).

During postoperative care, recipients were medicated according to the immunosuppression protocol (Wang et al., 2025), and appropriate treatments or nutritional supplements were given accordingly. The 2 recipients (M1 and M2) with transplanted kidneys survived for 23 and 16 days, respectively. In both recipients, the renal function indices of serum creatinine, urea and cystatin C remained relatively stable up to 10 days after surgery, and then increased gradually (Fig. 5A‒C). The red blood cell count remained stable all the times (Fig. 5D), while the platelets count first dropped to its lowest point on the day of transplantation then maintained at approximately 1 × 10^11^/L thereafter (Fig. 5E). The kidney xenografts showed a soft texture but many hemorrhagic spots at the end of the experiment (Fig. 5F). Hematoxylin-eosin (H&E) analysis showed that M1 recipient transplanted kidney manifested interstitial hemorrhage, inflammatory infiltration, tubular infarction and vacuolar degeneration. The M2 transplanted kidney showed erythrocyte stasis, neutrophil infiltration, tubular calcium salt deposition and small arteriolar thrombosis (Fig. 5G). Periodic acid–Schiff–Methenamine and Masson’s Trichrome (PASM + Masson) staining revealed small arteriolar-thrombi, interstitial fibrosis, cellular hyperplasia and tethered basement membrane hyperplasia in M1 transplanted kidney; lesions such as thrombus formation at the entrance of glomerular afferent arterioles, interstitial hemorrhage, glomerular cell hyperplasia and auricular (ear-like) deformation of the glomerular tuft in M2 kidney recipient (Fig. 5H).

**Figure 6.**
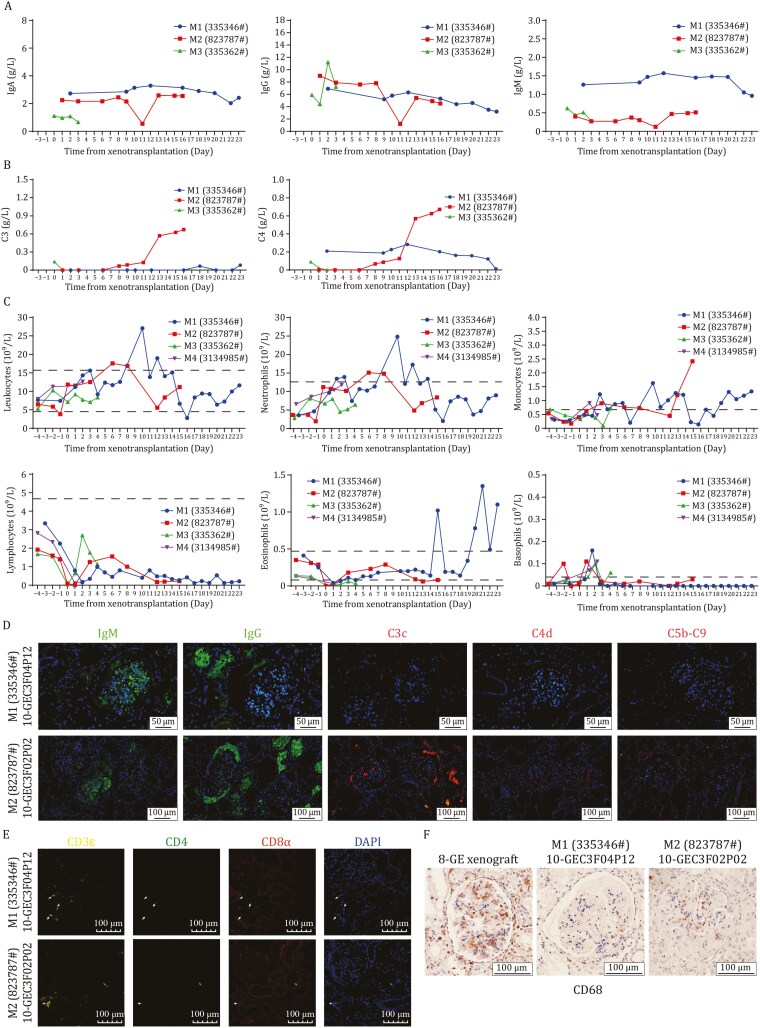
**Humoral, and cellular immune response of recipient macaques.** (A) The levels of IgA, IgG and IgM in the serum of recipient macaques. (B) The levels of complement C3 and C4 in serum of recipient macaques. (C) The numbers of white blood cells (WBCs), neutrophils, monocytes, lymphocytes, eosinophils and basophils in the whole blood of recipient macaques. (D) Antibodies and complement deposition in 10-GE porcine kidney xenograft confirmed by immunofluorescence. Scale bar = 100 µm. (E) T cell infiltration in porcine kidney xenograft confirmed by immunofluorescence (scale bar = 100 μm). (F) CD68^+^ macrophages infiltration in porcine kidney xenografts confirmed by immunohistochemistry, scale bar = 100 μm.

In terms of humoral immune rejection, the serum IgA, IgM and IgG antibodies of the 2 recipients were generally stable (Fig. 6A), and complement C3 and C4 were maintained at relatively stable levels in M1 recipient, whereas increased significantly in M2 recipient after 10 days of transplantation (Fig. 6B). The leukocyte and neutrophil counts were fluctuant in both recipients. The monocyte counts in both recipients increased significantly at the late stages of transplantation. The lymphocytes and basophils remained relatively stable, while eosinophils remained relatively stable in the M2 recipient, and basophils fluctuated greatly in the late stage of transplantation in M1 recipient (Fig. 6C). Immunofluorescence staining showed IgM and IgG antibody deposition, without complement C4d and C5b-C9 deposition in both kidney xenografts, except for C3c deposition in the M2 recipient (Fig. 6D). In terms of cellular immunity, we observed sporadic CD3^+^CD8^+^ T-lymphocyte infiltration, but no CD3^+^CD4^+^ T-cell infiltration (Fig. 6E). Notably, IHC result revealed that the 10-GE kidney xenografts in both recipients exhibited significantly reduced CD68^+^ macrophage infiltration when compared with 8-GE kidney xenografts (Fig. 6F).

Furthermore, all liver function indices, including total protein (TP), albumin (ALB), pseudocholinesterase (PCHE), total bilirubin (TBIL), total bile acid (TBA), aspartate aminotransferase (AST), alanine aminotransferase (ALT) and adenosine deaminase (ADA), remained relatively stable, except alkaline phosphatase (ALP) which tended to increase gradually (Fig. S7A‒I). In terms of electrolyte balance, the levels of sodium (Na), potassium (K), chloride (Cl), calcium (Ca), magnesium (Mg) and bicarbonate (HCO₃^−^) were stable, whereas blood phosphorus (P) increased significantly in the late stage of transplantation (Fig. S7J‒P).

## Discussion

Xenotransplantation entry into the clinics is imminent, so there is an urgent need to construct and select the safe donor pigs with suitable gene combinations. In recent years, with the rapid development of gene editing technology, GE donor pigs with various genetic combinations have emerged, and 10-GED pigs seem to be suitable for clinical xenotransplantation. However, editing multiple genes is comparatively difficult and time consuming. Meanwhile, cross-species transmission of pathogenic microorganisms poses a great challenge to the clinical application of xenotransplantation. Xenotransplantation can be used for clinical phase only if GED pigs are safe and have stable inheritance of the genes. In this study, we successfully produced 10-GED pigs using the CRISPR/Cas9 system, the PiggyBac transposase system, and somatic cell cloning. We presented large-scale screening of pathogenic microorganisms in these 10-GED pigs for the first time, which were negative for 47 tested pathogens, including porcine cytomegalovirus. These donor pigs exhibited normal reproductive ability, representing a significant step forward toward their utilization in clinical xenotransplantation.

Two companies, Revivicor and eGenesis, have been working on the development of xenotransplantation donor pigs for a long time. Currently, both companies have obtained more than 10-GED pigs, and have carried out several cases of pig-to-NHPs (Anand et al., 2023; Eisenson et al., 2024; Moazami et al., 2023), pig-to-brain-dead human (Moazami et al., 2023; Pan et al., 2024), and pig-to-living-patient xenotransplantation (Griffith et al., 2022, 2025; Kawai et al., 2025), thus greatly advancing the clinical application of xenotransplantation. The 10-GED pig developed by Revivicor contained the KO of 3 xenoantigens and growth hormone receptor (GHR), and overexpression of 2 hCRPs (hCD46 and hCD55), 2 coagulation regulatory proteins (hTBM and hEPCR), 1 macrophage phagocytosis-inhibiting gene (hCD47), and 1 anti-inflammatory gene (hHO-1). The 11-GED pig developed by eGenesis also contained the KO of 3 xenoantigens, and knock-in of 2 hCRPs (hCD46 and hCD55), 2 coagulation regulatory proteins (hTBM and hEPCR), 1 macrophage phagocytosis-inhibiting gene (hCD47), and 2 anti-inflammatory genes (hHO-1 and hA20), and inactivation of all PERVs (Anand et al., 2023). In the present study, we developed 10-GED pigs with the KO of 3 xenoantigens, and overexpression of 3 hCRPs (hCD46, hCD55 and hCD59), 3 coagulation regulatory proteins (hTBM, hEPCR and hCD39) and 1 macrophage phagocytosis-inhibiting gene (hCD47). Nevertheless, our donor pigs presented increased complement inhibition and anticoagulation regulation. Among them, hCD59 inhibits the formation of membrane-attacking complexes, which may further inhibit graft injury caused by human complement (Zhou et al., 2005). hCD39 has an inhibitory effect on inflammation along with anticoagulation (Dwyer et al., 2004). In addition, we used a native miniature pig breed with adult weights between 50‒80 kg (Chen et al., 2024, 2025), which is similar to the average weight of humans, and the heart and kidney organ sizes were closer to those of humans as shown by a large number of slaughtering experiments and measurements (Cheng et al., 2025). These characteristics avoided metabolic regulation disorders in pigs caused by KO of GHR gene (Anand et al., 2023).

The survival of xenografts is mainly determined by a combination of multiple factors, including genetic modifications, immunosuppressive strategies, the level of preformed natural antibodies against donor pigs in the recipient, surgical interventions, and postoperative care, etc., as evidenced by a wide range of 0‒337 days (Eisenson et al., 2024), and 6 days to > 429 days (Anand et al., 2023) survival of the renal xenografts from 10-GE, and 11-GE pigs into NHP, respectively. Similarly, we used expression of 7 genes at 3KO background and xenograft survived for 23 and 16 days with no wonder that our genetic modifications successfully alleviated immune rejection, and the pig heart, kidney, and liver can functionally support the life to the recipient macaques.

To shorten the development cycle of donor pigs, multiple plasmids can be co-transfected to generate cells with multiple gene edits. However, this approach also requires the screening and identification of a larger number of single-cell colonies to obtain cell lines suitable for SCNT. During the first transfection and screening process, we obtained a total of 49 single-cell colonies, and eventually only 4 colonies (C2#, C3#, C10#, and C24#) were 8-GE. Moreover, these four colonies contained all WT genotypes, and among them, three cell colonies, C2#, C10#, and C24#, might have KO of single-allele of CMAH gene, and the deleted fragment was a multiple of 3. Therefore, we finally selected the C3# colony for cloning, and obtained 13 fetal pigs, whose genotypes of GGTA1, CMAH, and β4GalNT2 genes were biallelic KO. However, a 53-bp deletion which was exactly a 30-bp deletion in corresponding region when calculated from the start codon ATG, in one of the alleles of GGTA1 gene, which was observed in 8-GEC fetuses and pigs produced by cloning from cell colony C3#, which possibly lead to the formation of a truncated protein in GGTA1 gene (Yang et al., 2021), which could still catalyze the synthesis of the αGal antigen. Therefore, during the second transfection, we finally succeeded in inactivating the GGTA1 gene by targeting at the exon 8 of GGTA1 gene.

Despite ongoing research, clinically effective and safe strategies for monocyte inhibition in xenotransplantation remain limited. In our previous study, varying degrees of renal failure with oliguria or anuria were observed at the end of the transplantation period, possibly due to extensive and severe glomerulosclerosis. The presence of a large number of macrophage aggregates in the glomeruli of transplanted porcine kidneys suggests that glomerulosclerosis may be related to the abnormal activation of macrophages (Wang et al., 2025). However, in the present study, overexpression of hCD47 significantly reduced the number of macrophage infiltrations in the glomeruli of kidney xenografts.

IgM and IgG antibody deposition was observed in both porcine kidney xenografts in this study, which was consistent with porcine heart xenografts utilizing the 10-GE pig developed by Revivicor (Singh et al., 2025). Thus, it is intriguingly speculated that the pig-to-NHPs xenotransplantation is still characterized by the presence of fourth xenoantigen, which may lead to the development of AMR (Hara et al., 2023). Contrarily, no significant AMR was found in numerous cases of multiple GE pig-to-human xenotransplantation in which three xenoantigens were knocked out (Loupy et al., 2023; Porrett et al., 2022), which also suggests that NHP may not be an ideal model for studying the effectiveness of GED pigs in humans.

Biosafety risks are also important impediments to the clinical application of xenotransplantation. In human history, there has never been a shortage of viral invasions that have caused profound disasters for nations. For example, the HIV virus, which spread globally in the 1980s infected more than 40 million people worldwide (Simon et al., 2006). In 2019, a global outbreak of COVID-19 occurred, with more than 700 million people infected and more than 7 million deaths due to COVID-19 infection (Marks and Gulick, 2023). Porcine organ transplantation to humans also faces the transmission and spread of pathogenic microorganisms. Strict screening, prevention, and control measures during the breeding and production of donor pigs can prevent the spread of pathogenic microorganisms. Keeping in view the WHO requirements for the control of pathogenic microorganisms in xenotransplantation donor pigs (Fishman et al., 2012), we conducted a multi-unit, multi-sampled, multi-method large-scale testing of 10-GEC pigs for the first time, and found that all 47 pathogenic microorganisms were absent except *Streptococcus suis*, which can be completely cleared by the use of antibiotics, indicating bio-secure production of our 10-GED pigs. However, a small number of pathogenic microorganisms could not be identified at that time due to the existence of numerous variants and lack of reliable detection methods. Therefore, the establishment of a sound screening method for pathogens screening in GED pigs is extremely important for promoting the clinical application of xenotransplantation.

It is worthwhile to mention that on May 17, 2024, our 10-GEC pig liver was transplanted into a 71-year-old patient with massive liver cancer, making it the fifth case of xenotransplantation from a pig to a human patient worldwide. The patient is in good condition after the operation, and the pig liver can secrete bile in the human body without any signs of hyperacute or acute rejection (Mallapaty, 2024), further confirming the effectiveness of our 10-GED pigs.

In conclusion, we successfully obtained 10-(GTKO/CMAHKO/β4GalNT2KO/hCD46/hCD55/hCD59/hTBM/hCD39/hEPCR/hCD47) GED pigs by gene editing and somatic cell cloning technology, which have normal reproductive ability. We presented large-scale screening of pathogenic microorganisms in these 10-GED pigs for the first time, which were tested negative for 47 pathogens. The effectiveness of the 10-GED pigs was confirmed by pig-to-NHP xenotransplantation, thus providing a suitable donor pig for future clinical trials.

## Supplementary data

Supplementary data is available at *Protein & Cell* online https://doi.org/10.1093/procel/pwaf075.

pwaf075_Supplementary_Data

## Data Availability

All the data generated is available in the manuscript and can also be acquired from the corresponding authors upon special request. The raw sequencing data generated in this study can be accessed on the Genome Sequence Archive (GSA) under the accession CRA029024 (ngdc.cncb.ac.cn/gsa/browse/CRA029024).

## Code availability

No specific custom script was used in this study, and if applicable, will be accessed with reasonable requests.
