# Supplementary material for: Specific pathogen free ten gene-edited donor pigs for xenotransplantation
Source: Protein Cell. 2025 Aug 25;16(12):1002–16. doi: 10.1093/procel/pwaf075 (PMC12742852; doi:10.1093/procel/pwaf075)
Supplement: pwaf075_Supplementary_Data [file pwaf075_supplementary_data.zip › pwaf075_suppl_Supplementary_Figures_S1-S7_Tables_S3-S20.docx]

**Supplemental Materials**

**Methods**

All experimental animals and surgical procedures were approved by the Animal Ethics Committee of Yunnan Agricultural University (202405004). The Tibetan macaques were purchased from the institute of Laboratory Animals of Sichuan Academy of Medical Sciences & Sichuan Provincial People's Hospital, China.

**Vector construction**

sgRNAs were designed based on CRISPR/Cas9 technology to simultaneously target porcine GGTA1, CMAH, β4GalNT2 genes. Meanwhile, the PiggyBac system was utilized to overexpress human genes using human EF-1α promoter (hCD46, hCD55 and hCD59), the human endothelial cell-specific promoter ICAM2 promoter (hTBM and hCD39)(Wang et al., 2025), and CAG promoter (hEPCR and hCD47) (Fig. S1).

**Cell transfection and screening and characterization**

Cas9, sgRNA and human transgene overexpression vectors were transfected into fetal fibroblasts using electroporation, and antibiotic was added for screening at 48 h post-transfection, and surviving cells were cultured by limited dilution to obtain single-cell colonies. The genotypes of the obtained colonies were detected by PCR and Sanger sequencing (Primers are shown in Table S1), and the cells with TKO of xenoantigens along with integration of human transgenes were selected as the donor for SCNT.

**Somatic cell nuclear transfer and embryo transfer**

Oocyte collection, in vitro maturation, SCNT and embryo transfer were performed as described previously (Wei et al., 2013). Briefly, cultured cumulus-oocyte complexes were isolated from cumulus cells by treating with 0.1% (w/v) hyaluronidase. The oocytes with first polar body were selected and cultured in porcine zygote medium-3 (PZM-3) media containing 10% FBS, 0.0171g/mL sucrose, and 0.01μg/mL colchicine and incubated at 38°C, 5 % CO_2_ incubator for 0.5~1 h. The first polar body was enucleated via gentle aspiration using a beveled pipette in TLH-PVA, while the donor cells were injected into the perivitelline space of the enucleated oocytes by using micromanipulator (HMS-320D, Hariolab, Hua Yue Instrument Technology Co., Ltd. China). The reconstructed embryos were fused with a single direct current pulse of 25V/mm for 20 µs using the Electro Cell Fusion Generator (HCF-230, Hariolab, Hua Yue Instrument Technology Co., Ltd. China; LF201, NEPA GENE Co., Ltd., Japan) in fusion medium. Embryos were then cultured in PZM-3 for 0.5–1 h and activated with a single pulse of 150 V/mm for 100 µs in activation medium. The embryos were equilibrated in PZM-3 supplemented with 5 µg/mL cytochalasin B for 2 h at 38.5°C in a four-compartment incubator (DI-4R, Hariolab, Hua Yue Instrument Technology Co., Ltd. China) and then cultured in PZM-3 medium in a humidified atmosphere with 5% CO_2_, 5% O_2_, and 90% N_2_ (APM-30D, ASTEC, Japan) until embryo transfer. The SCNT embryos were surgically transferred into the oviducts of the recipients, and a viable fetus was obtained through caesarian section after 33 days of pregnancy. A fetal fibroblast cell line was established, and subjected to PCR, and, Sanger sequencing for identification. Fetuses with biallelic TKO, and targeted overexpression of human transgenes were selected as donor cells for recloning. After completion of gestation period (~114 days), GEC piglets were obtained through natural delivery and identified by the same way of fetus identification.

**Genotyping of cell lines, cloned fetus and piglets**

The screened cell colonies were directly lysed, and the whole genomic DNA of fetal tissues and ear tissues of cloned piglets were extracted using high-salt precipitation, and used as template for PCR, and the target gene fragments were amplified by PCR reaction. For cell colonies, we first confirmed the integration of five transgenes by gel electrophoresis, and then used the Sanger sequencing analysis to determine the genotypes of the target regions of three xenoantigens and finally the cells with the simultaneous KO of the three genes along with successful integration of the human transgenes were selected as donor cells for cloning. The cloned fetuses and piglets were analyzed by PCR, and Sanger sequencing. The PCR reaction system (50 µL) was 400 ng of template, 2 µL of up and downstream primers, 1 µL of dNTP, 1 µL of Taq DNA polymerase (Cat #P505, Vazyme), 25 µL of buffer. The PCR reaction program was 95°C for 5 min; 95°C for 30 s, 68~58°C (annealing temperature decreased by 1°C per cycle) for 30 s, 72°C for 1 min, 10 cycles; 95°C for 30 s, 58°C for 30 s, 72°C for 1 min, 25 cycles; and 72°C for 7 min. The primers are shown in Table S1.

**Quantitative polymerase chain reaction (qPCR)**

In order to evaluate the mRNA expression levels of seven transgenes, total RNA from heart, liver, lung, kidney tissues of WT and GEC pigs was extracted using TRIzol reagent (Cat#ET111-01-V2, TransGen Biotech) according to the manufacturer's instructions, and the concentration and RNA quality were detected. Complementary DNA (cDNA) was synthesized from total RNA using a PrimeScript RT reagent Kit (Cat#RR047B, TaKaRa) and was used as a template for qPCR in TB green-based qPCR instrument (CFX-96, Bio-Rad, USA). The reaction was performed in a 20 µL reaction mixtures comprising 10 µL of 2×TB Green® Premix Ex Taq™ (Tli RNaseH Plus) (Cat#RR420A, TaKaRa), 1 µL of cDNA, 1 µL of forward primer, 1 µL of reverse primer, and 7 µL of ddH_2_O (primers listed in Table S1). The reaction program is as follows: 95°C for 30 s, followed by 40 cycles of 95°C for 10 s, and 62°C for 45 s. Three technical replicates were conducted for each sample and the relative expression levels of target genes were quantified by 2^-ΔΔct.^

**Western blot**

The samples were lysed in RIPA lysis buffer (Cat#BB‑3201‑2; Bestbio) with protease inhibitor (Cat#D1201‑2; TransGen Biotech) at 4°C. Then, the total protein concentrations of the tissue lysates were determined using a BCA Protein assay kit (Cat#P0009‑1 & 2; Beyotime Biotech). Protein samples were then separated via 10% and 15% SDS–PAGE, and subsequently transferred to polyvinylidene fluoride (PVDF) membrane. The membranes were incubated at room temperature (RT) for 30 min in 5% BSA solution with gentle shaking to block non‑specific binding before incubation with the diluted primary antibody (Table S2). Subsequently, membranes were incubated with horseradish peroxidase‑conjugated secondary antibody (Table S2) for 2 h at RT. Membranes were first washed three times and then incubated with reagent from an Easysee Western Blot kit (Cat#DW101-01, TransGen Biotech) and visualized using an imaging system (ChemiDocTM MP Imaging System, Bio‑Rad Laboratories, Inc.).

**Droplet digital polymerase chain reaction (ddPCR) for transgenic copy number**

The samples including the human whole blood (positive control, n=1), pre-transfected porcine cells (negative control), drug-selected cell lines, fetal cells, and the heart, liver, lung, kidney, spleen and other tissues of the GEC pig were collected and DNA was extracted. Then, these DNA samples were digested by MseI (Cat#FD0984, ThermoFisher), EagI (Cat#FD0334, ThermoFisher) and AsisI (Cat#FD2094, ThermoFisher) restriction endonuclease at 37°C for 2 h, and inactivated at 65°C and 85°C for 20 min. The digested product was diluted to 5ng/µL and used as a template for ddPCR. The primers and probes corresponding to hCD46, hCD55, hCD59, hTBM, hCD39, hEPCR, hCD47 and GAPDH genes (Table S1), ddPCR super mix (Cat#1863024, Bio-Rad), and DNA template were mixed to prepare a 25 µL reaction mixture. Then, ddPCR droplets were generated with QX100 Droplet Generator (Bio-Rad) and transferred to a 96 well plate. PCR reactions were performed at 94°C for 5 min, 94°C for 30 s, 56°C for 1 min (40 cycle), and 98°C for 10 min. After the reaction, the 96 well plate was placed in Bio-Rad Droplet Reader and QunataSoft Software was used to set up the experimental design and read the experiment. Once the program was finished, the copy numbers were analyzed through gating, according to the manufacturer’s instructions (Bio-Rad).

**Immunofluorescence staining**

The paraffin-embedded tissue blocks were cut into 2.5 µm, transferred to glass slides, dewaxed using xylene and gradient alcohol, retrieved antigens with EDTA buffer (Cat#G1207, Servicebio) at 92–98°C for 15 min, and cooled at RT. Then, sections were washed with phosphate buffer saline (PBS) for three times (each time 3 min), incubated with autofluorescence quencher A (Cat#G1221, Servicebio) at RT in dark for 15 min, washed with PBS for three times again, incubated with fetal bovine serum (FBS) at RT for 30 min, and dried. The dried sections were the incubated with corresponding antibodies (Table S2). For visualization, corresponding secondary antibodies (Table S2) were diluted with PBS containing 10% FBS (v/v = 1:200) and used to incubate sections at 4°C in dark for 2 h, and a negative control was incubated with PBS containing 10% FBS. Then, sections were washed with PBS three times and stained with DAPI (Cat#G1012, Servicebio) for 3 min. After washing with PBS for 1 min, autofluorescence quencher B (Cat#G1221, Servicebio) was added for 5 min and washed three times again. Finally, sections were mounted with anti-fluorescence quencher (Cat#G1401, Servicebio) and imaged using an OLYMPUS BX53 fluorescence microscope, and the fluorescence intensity of different samples was compared using ImageJ software.

**Large**-**scale screening of pathogenic microorganisms in 10**-**GEC pigs**

Whole blood was collected from 10-GEC pigs through the anterior vena cava, and the serum was separated. Nasal, oral, and pharyngeal swabs were collected from GEC pigs, and DNA, RNA extractions, cDNA synthesis and PCR and qPCR were performed as reported elsewhere in the methods. The samples were processed accordingly for direct smear method (Ashiri et al., 2021), saturated saline floating method (Ursache et al., 2021) and centrifugal precipitation method (Kumar et al., 2018). Meanwhile, the samples collected above were sent to Vital River Laboratories (VRL) in China for pathogenic microorganisms’ detection, and the metagenomics sequencing analysis was carried out at Hangzhou Matridx Biotechnology Co., Ltd. and Beijing Novogene Co., Ltd.

**H&E staining**

Tissue sections (2-µm-thick) were stained with H&E, PASM + Masson following the manufacturer’s instructions. Briefly, for H&E staining, deparaffinization was followed by incubation with 0.5% hematoxylin (Harris) for 10-15 min, then were differentiated in 1% hydrochloric acid in 70% ethanol, followed by bluing in 1% lithium carbonate solution. Subsequently, slides were counterstained with 1% eosin for 3-5 min, dehydrated through a graded alcohol series, cleared in xylene, and mounted with coverslips. For PASM + Masson staining, the paraffin-embedded kidney tissue sections were deparaffinized and rehydrated with distilled water, followed by oxidation in 1% periodic acid for 15-20 min. The sections were then incubated in preheated methenamine silver working solution at ≥70 °C for 20-25 min, differentiated with gold chloride, and treated with 2% sodium thiosulfate for 2 min. After thorough washing, the sections were counterstained using the Masson’s trichrome protocol: incubated in Bouin’s solution at 60 °C for 1 h and rinsed in tap water to remove background staining, followed by sequential staining with Celestine blue and Mayer’s hematoxylin, acid fuchsin solution for 15-20 min, phosphomolybdic acid for 5-10 min, and aniline blue for 3-5 min. Finally, the sections were rapidly dehydrated in absolute ethanol, cleared in xylene, and mounted with coverslips. This combined staining procedure enables simultaneous visualization of basement membranes (black) and collagen deposition (blue) within the same section. The percentage of glomeruli showing cell hyperplasia, endocapillary hypercellularity, glomerular sclerosis, or crescent formation was determined by counting 50 randomly sampled glomeruli under light microscopy at 630x magnification. Tubular atrophy lesions were evaluated based on the proportion of the atrophic area to the total interstitial area.

**Whole genome sequencing (WGS)**

Whole genome sequencing was performed as reported previously (Wang et al., 2025). Briefly, the samples were derived from 10-GEC pigs (10-GEC3F04P16P05, 10-GEC3F04P16P10, and 10-GEC3F04P12) and WT fetal tissue (F14) which was used for cell line establishment to perform 10 gene editing, and were used for DNA extraction. A total of 0.2 μg of DNA sample was used as input material for DNA library preparation. Briefly, the genomic DNA was fragmented to approximately 350 bp using a Covaris LE220R-plus (Covaris, USA). The DNA fragments were then end-polished, A-tailed, and ligated with full-length adapters for Illumina sequencing, followed by PCR amplification. The PCR products were purified using the AMPure XP system (Beckman Coulter, Beverly, USA). Library quality was assessed on the Agilent 5400 system (AATI) and quantified by real-time PCR (1.5 nM). After library quality control, different libraries were pooled based on effective concentration and targeted data amount. The 5' end of each library was phosphorylated and cyclized. Subsequently, loop amplification was performed to generate DNA nanoballs. These DNA nanoballs were then loaded onto the flow cell of the DNBSEQ-T7 for sequencing at BioHuaxing Bioinformatics Technology Co., Ltd (Beijing, China). In total, deep sequencing of samples with the depth of >50× per individual. Subsequently, quality control (QC) analysis was conducted by using the fastp software with default parameters to remove low-quality reads and ensure the reliability of downstream analysis.

**Identification of insertion location of transgenes**

Identification of insertion location of transgenes was performed as reported previously (Wang et al., 2025). To elucidate the integration site of an insert sequence, quality-controlled sequencing reads were aligned to this insert sequence utilizing the bwa-mem2, followed by processing with samtools software for generating the alignment file in BAM format. From this BAM files, we extracted reads that exhibit alignment to regions flanking both sides of the insert sequence. Given the premise that upon integration into the host genome, reads encompassing the junctions between the insert and genomic DNA will contain sequences alignable to both the insert and the porcine reference genome (GCF_000003025.6_Sscrofa11.1), these flanking reads were subjected to a secondary alignment against the genome (GCF_000003025.6_Sscrofa11.1) using the same methodology. The insertion locus was subsequently inferred based on the results of both alignments.

To construct a new reference genome that includes the integrated insert sequence, the insertion site was identified and the insert sequence was concatenated with the genomic sequence at this precise location. Following above method, all sequencing reads were aligned to this new composite reference genome. Subsequently, for detailed characterization of the integration event, the depth information of a specific region encompassing the insert sequence along with 20 kb of upstream and downstream flanking sequences was extracted and was performed on this region using a sliding window approach (window=100bp). The resulting data were then visualized with Python for the number of copies of the inserted fragment.

**Off-target analysis by WGS**

For off-target effect validation as reported previously (Wang et al., 2025), the offline version of Cas-OFFinder software was utilized to predict potential off-target sites within the pig genome (GCF_000003025.6_Sscrofa11.1). The prediction was based on the NGG protospacer adjacent motif (PAM) and allowed for a maximum of 4 base mismatches. Following the identification of candidate off-target loci, we proceeded with treatment-control variant calling using the Mutect2 tool from GATK. This analysis compared the treatment group against the control group to detect variants potentially induced by the CRISPR/Cas9 system. Subsequently, the variant profiles of 10-GE samples were aggregated and analyzed to identify recurrent variants. Variants that occurred at a higher frequency across multiple samples were highlighted as candidates for further investigation, indicating their potential significance as true off-target effects or other relevant genomic alterations.

**Flow cytometry**

The cells once attained a confluence of 70-80% were collected, digested with trypsin, and centrifuged at 1200 rpm for 3 min. Then cells were resuspended with 50 μL PBS and incubated with primary antibodies (Table S2) according to 1×10^3^ cells/μL at RT in the dark for 15-30 min. After that, cells were washed with PBS and incubated with corresponding secondary antibodies at RT in the dark for 30 min. Cells were washed with PBS and detected by using CytoFLEX flow cytometer (Beckman Coulter, USA). Data were analyzed by FlowJo-V10.8.1 software.

**Antigen-antibody binding assay**

Whole blood (10 mL each) from one healthy volunteer was collected and serum was separated. Meanwhile, 10 mL of whole blood from WT), and 10-GECD pigs were collected to isolate PBMCs. Based on the antigen-antibody binding assay, inactivated serum from human was incubated with WT and 10-GECD pig PBMCs, and then treated with anti-IgG antibody (Cat#628411, Invitrogen) and anti-IgM antibody (Cat#A18842, Invitrogen), respectively, and the levels of binding of IgG and IgM to pig cells was detected by cell flow cytometry.

**Complement-dependent cytotoxicity assay**

The inactivated human serum was diluted (1:1) in staining buffer (PBS containing 1% FBS). And PBMCs derived from WT and 10-GEC pig were collected, washed twice and resuspended in staining buffer. 1×10^5^ cells were incubated with 50 µL inactive Tibetan macaques’ serum (test group) or 50 µL PBS (negative control) for 30 min at RT. Then cells were washed with cold staining buffer to terminate reaction and incubated with rabbit complement sera (1:3 dilution, Cat#S7764, Sigma) for 30 min at RT. Finally, cells were stained with PI staining solution (Cat#GA1174, Servicebio) for 2 min, and analyzed for cell death using a CytoFLEX flow cytometer (Beckman Coulter, USA).

**Coagulation assay**

Endothelial cells (1×10^6^ cells/well) were inoculated in 12-well plates and incubated at 37°C for 24 h. The cells were subsequently washed with PBS followed by assay buffer (50 mM Tris HCl, 150 mM NaCl, 25 mM CaCl₂, 0.1% BSA, pH 7.5, 37°C). Next, 100 μL of 20 U/mL thrombin (Cat#9002-04-4, Absin) or human thrombin was added to each well and incubated for 30 min at 37°C. Then supernatant was subsequently discarded the and mixture was transferred to a 96-well flat-bottom plate, and 10 μL of 1 mM D-Lys(Z)-Pro-Arg-pNA (Cat#HY-P0021A, MCE), was added. The supernatant was analyzed in the zymometer at an absorbance of 405 nm at 37°C, and the concentration of unconjugated thrombin in the supernatant was calculated from the standard curve of thrombin.

**Macrophage phagocytosis assay**

Differentiation of the human macrophage cell line THP-1 was ensured by observing the differentiation status of the cells by incubating them for 3 days with 200 ng/mL PMA (Cat#16561-29-8, Sigma) in THP-1-specific medium. The 8-TG pig’s aortic endothelial cells (PAECs), 10-TG PAECs, WT PAECs, and human umbilical vein endothelial cells (HUVECs) (as the target cells) were stained with the fluorescent stain CFSE (Cat#ab113853, Abcam), after which targeted cells were incubated with human differentiated THP-1 cells (as effector cells) at a 1:1 ratio at 37°C for 6 h. Macrophages stained with an anti-human CD11b antibody (Cat#abs180040-25T, Absin) phagocytosed the CFSE-labeled target cells, which were then measured using FACS. The phagocytic activity was calculated as the number of phagocytosed macrophages/total number of macrophages.

**Kidney, heart and liver xenotransplantation from 10-GEC pigs to NHPs**

10-GEC pig-to-NHP xenotransplantation was conducted at the Yunnan Province Xenotransplantation Engineering Research Center of Yunnan Agricultural University, where the surgical team from the First Affiliated Hospital of Anhui Medical University, and First Affiliated Hospital of Zhejiang University School of Medicine performed the kidney, heart, and liver xenograft transplants to Tibetan macaques. Urine and blood biochemistry, immune indices, coagulation function, infection, immunosuppressant concentration and other tests were conducted regularly after the operation, and the blood flow of the transplanted kidneys was detected through ultrasound. Clinical medical personnel and veterinary professionals rotated caretaking duties for the recipients, and various test indices as well as the mental status of the recipients were analyzed, and treated accordingly.

**Statistical analysis**

The data are expressed as the means ± standard deviations of at least three independent replicates, and comparison between two groups of data were performed by Student’s *t*-test to determine significance (**P*<0.05, ***P*<0.01) using GraphPad Prism software.

**References**

Ashiri A, Rafiei A, Beiromvand M, et al. Screening of Strongyloides stercoralis infection in high-risk patients in Khuzestan Province, Southwestern Iran. *Parasit Vectors* 2021;**14**:37.

Kumar S, Sundararaj P, Kumara H N, et al. Prevalence of gastrointestinal parasites in bonnet macaque and possible consequences of their unmanaged relocations. *PLoS One* 2018;**13**:e0207495.

Ursache A L, Györke A, Mircean V, et al. Toxocara cati and Other Parasitic Enteropathogens: More Commonly Found in Owned Cats with Gastrointestinal Signs Than in Clinically Healthy Ones. *Pathogens* 2021;**10**:198

Wang Y, Chen G, Pan D, et al. Pig-to-human kidney xenotransplants using genetically modified minipigs. *Cell Rep Med* 2024;**5**:101744.

Wei H, Qing Y, Pan W, et al. Comparison of the efficiency of Banna miniature inbred pig somatic cell nuclear transfer among different donor cells. PloS one 2013;**8**:e57728.

**Supplemental Figures**

Figure S1


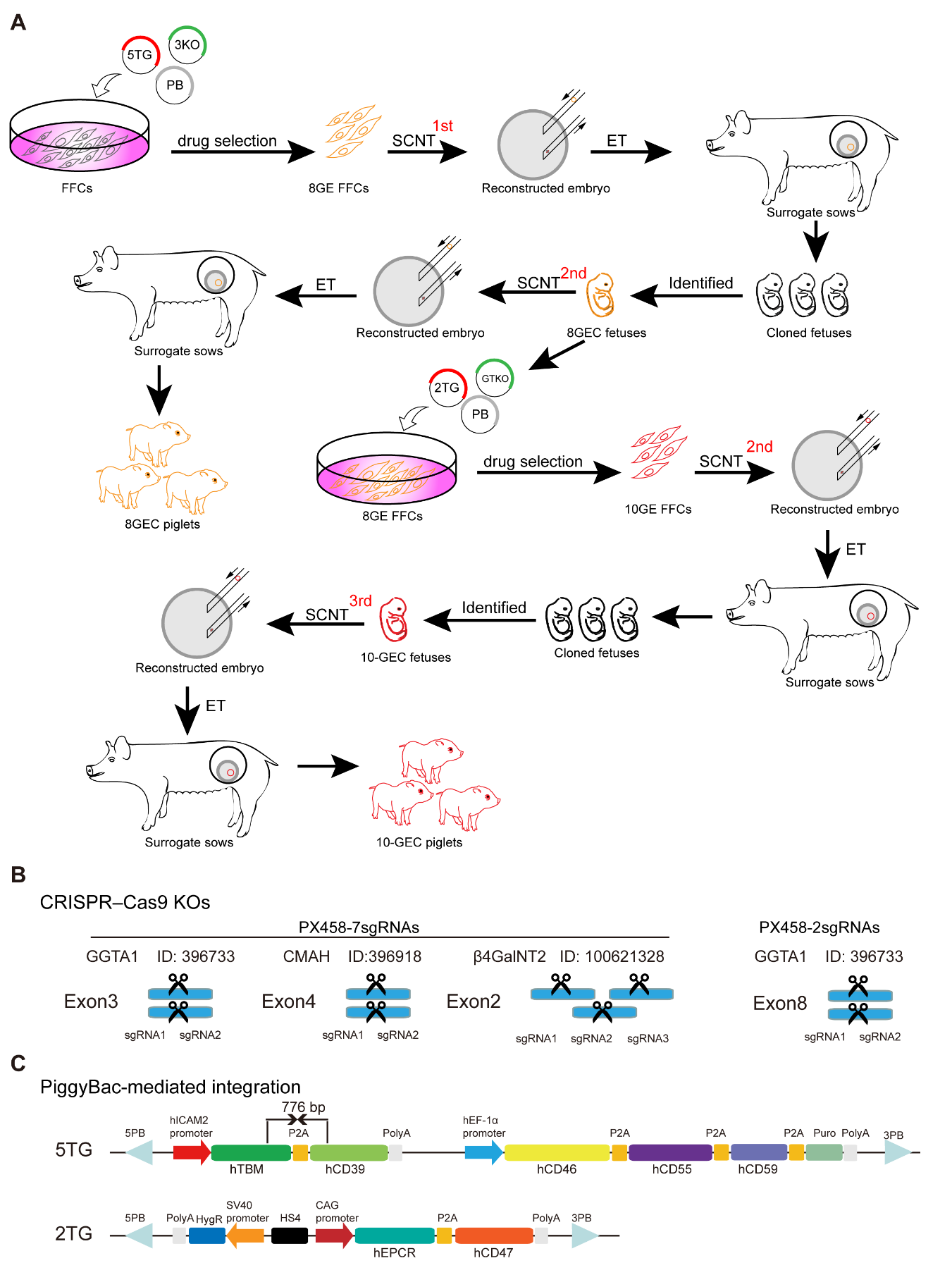


Figure S2


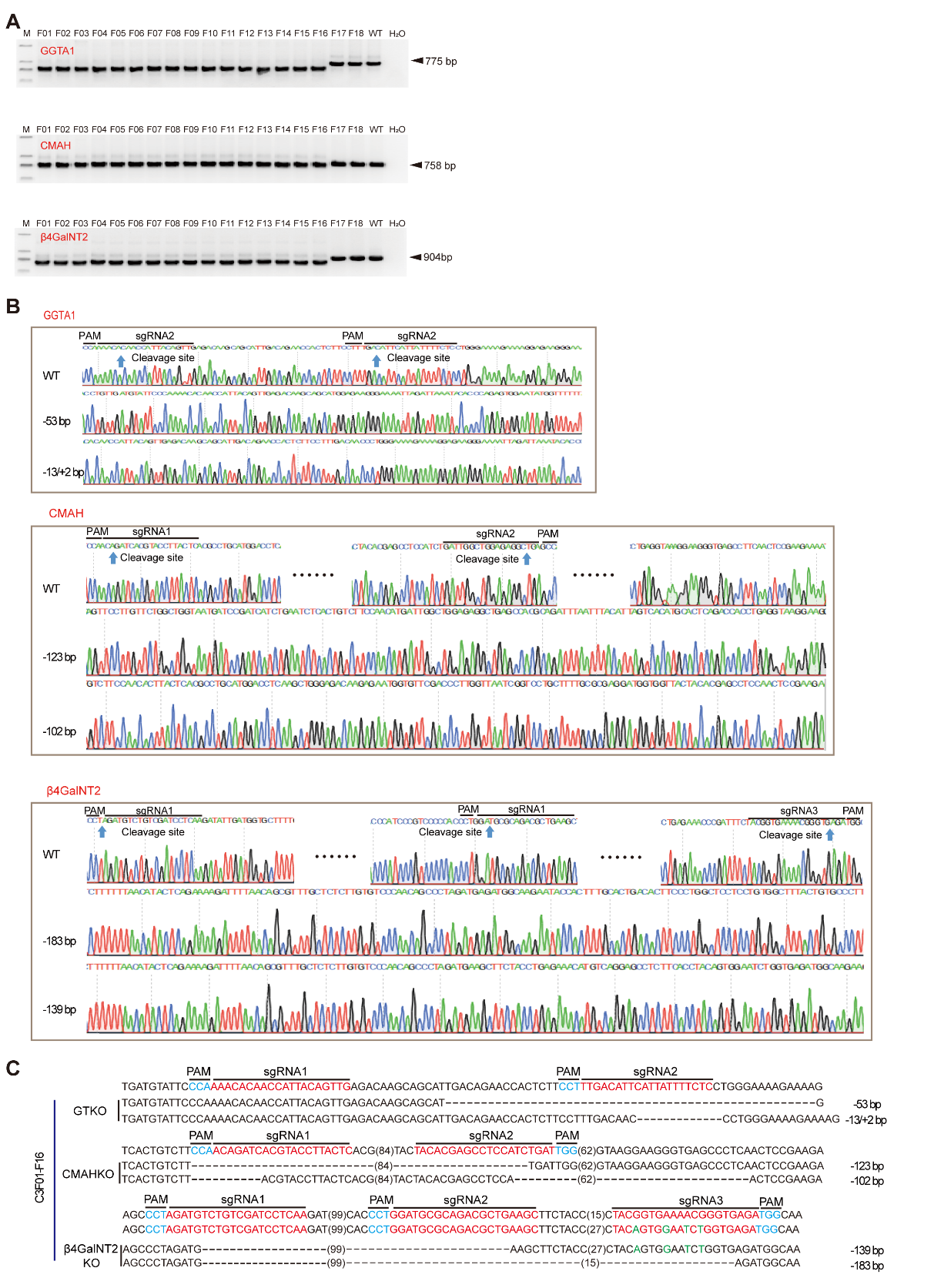


Figure S3


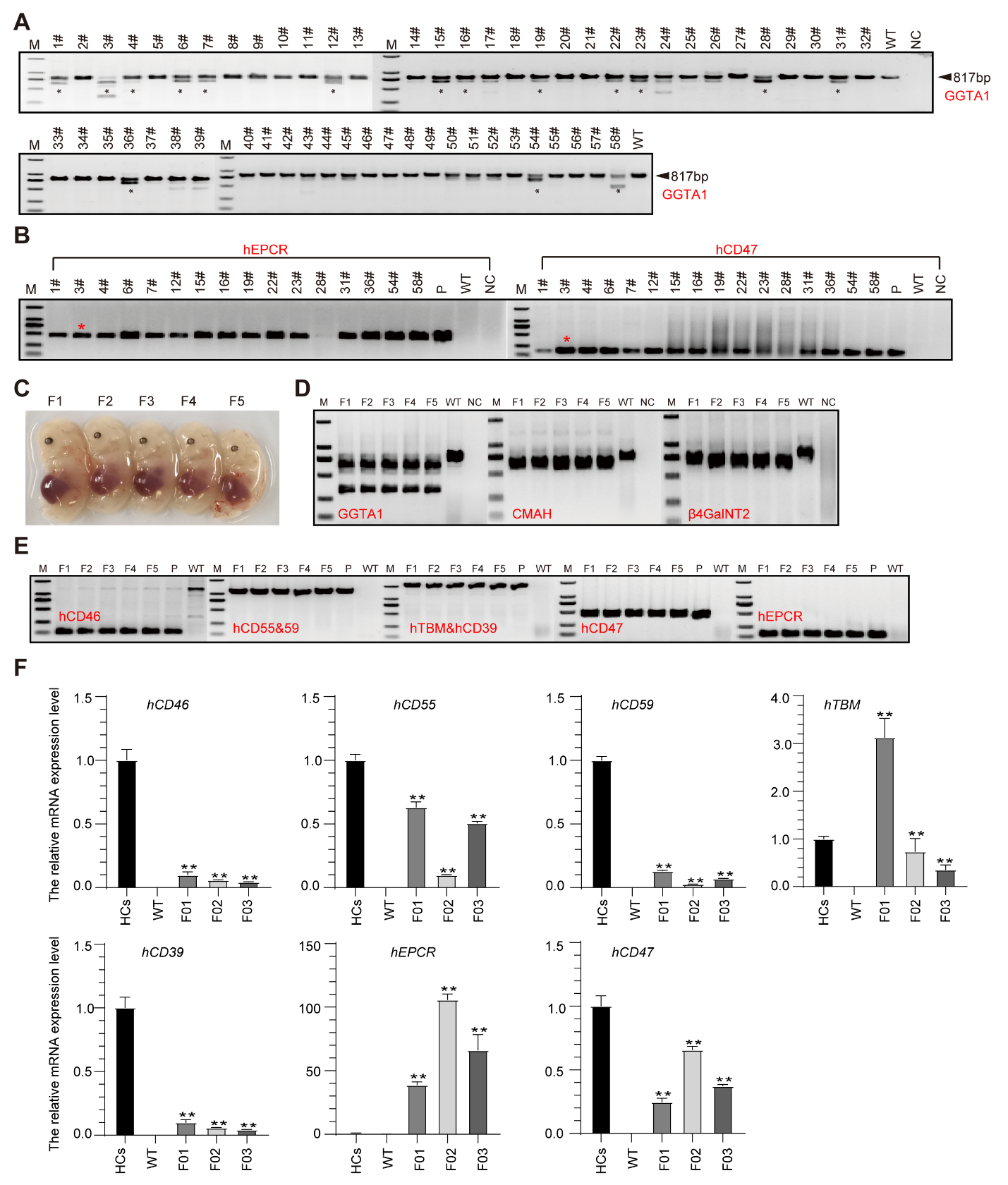


Figure S4


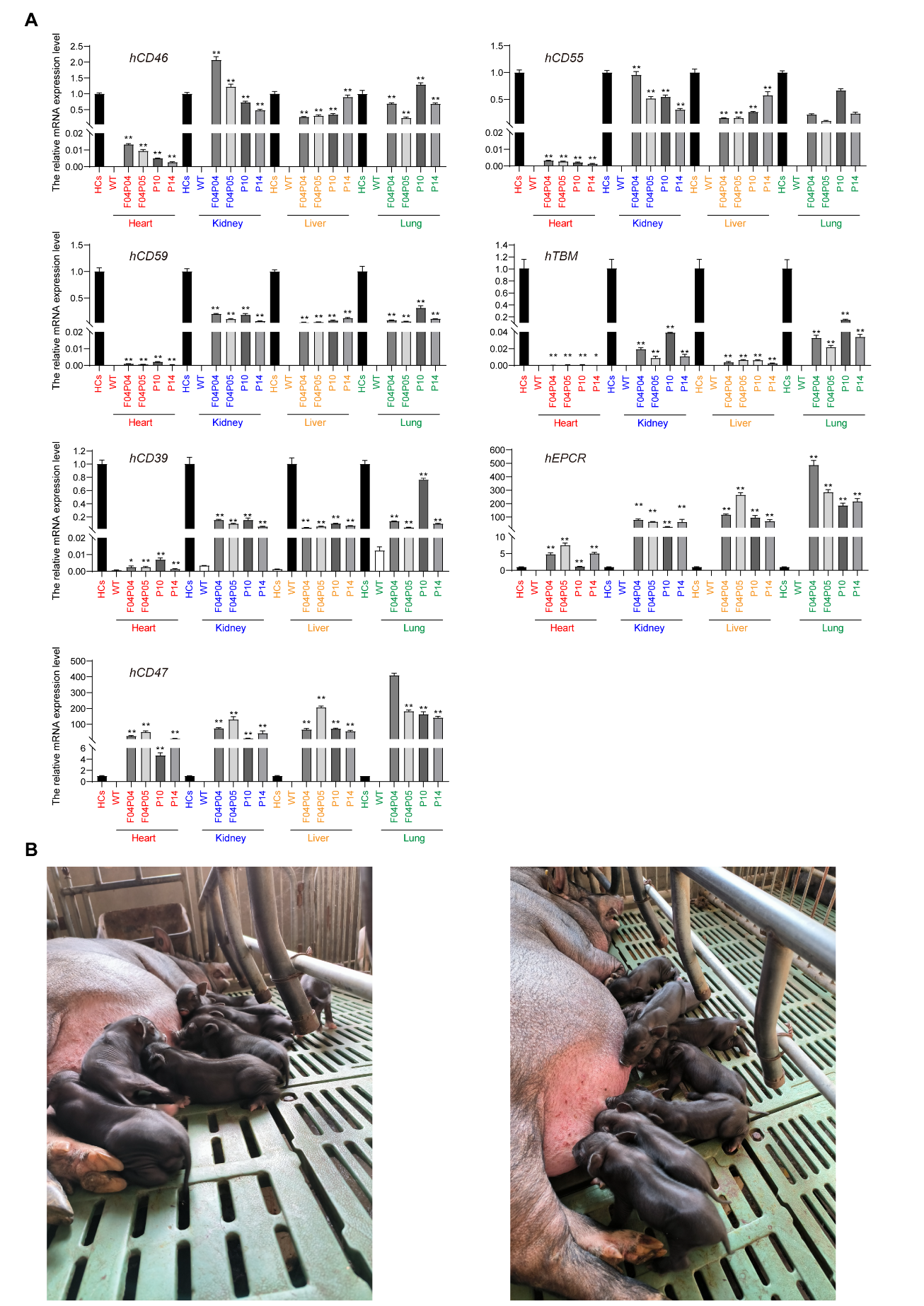


Figure S5


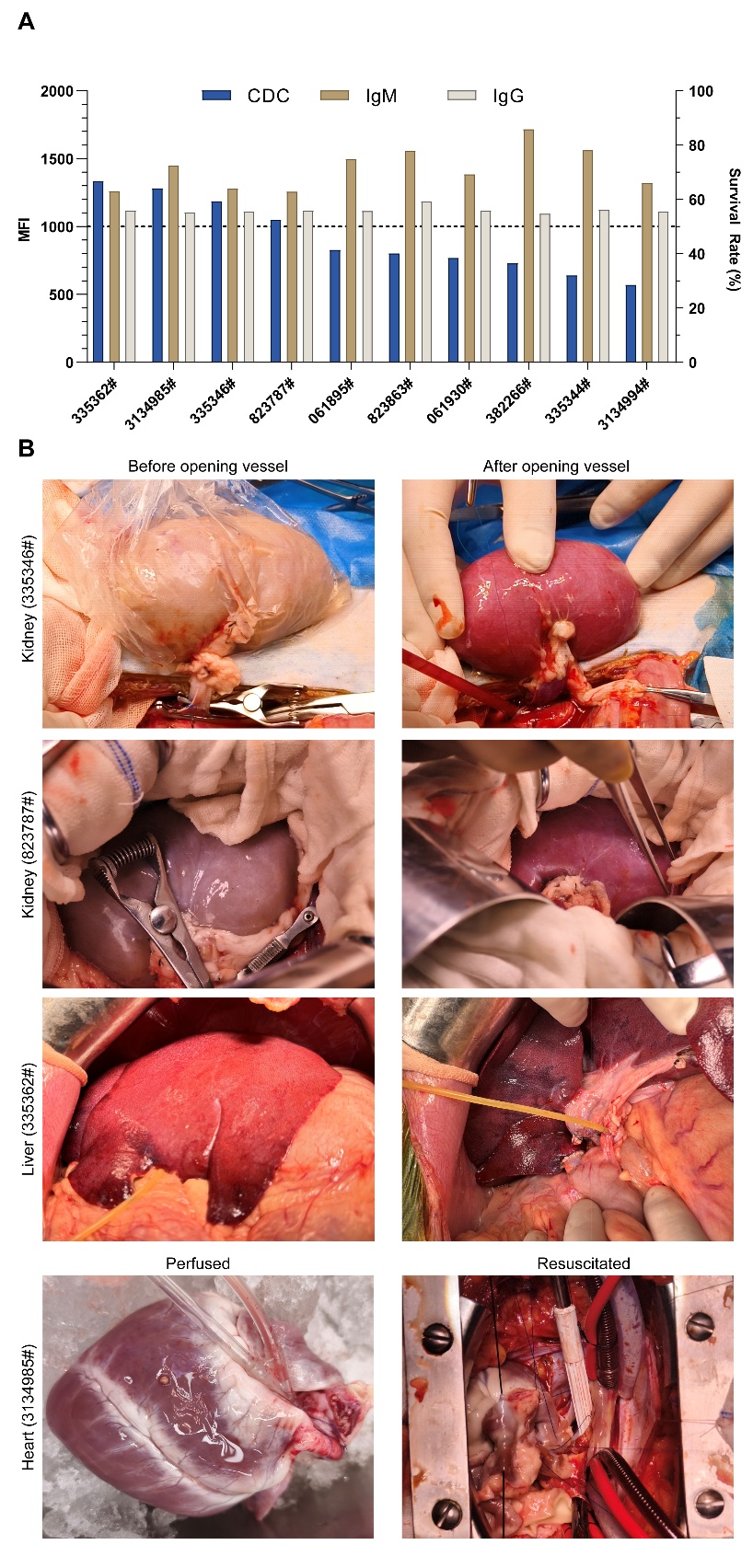


Figure S6


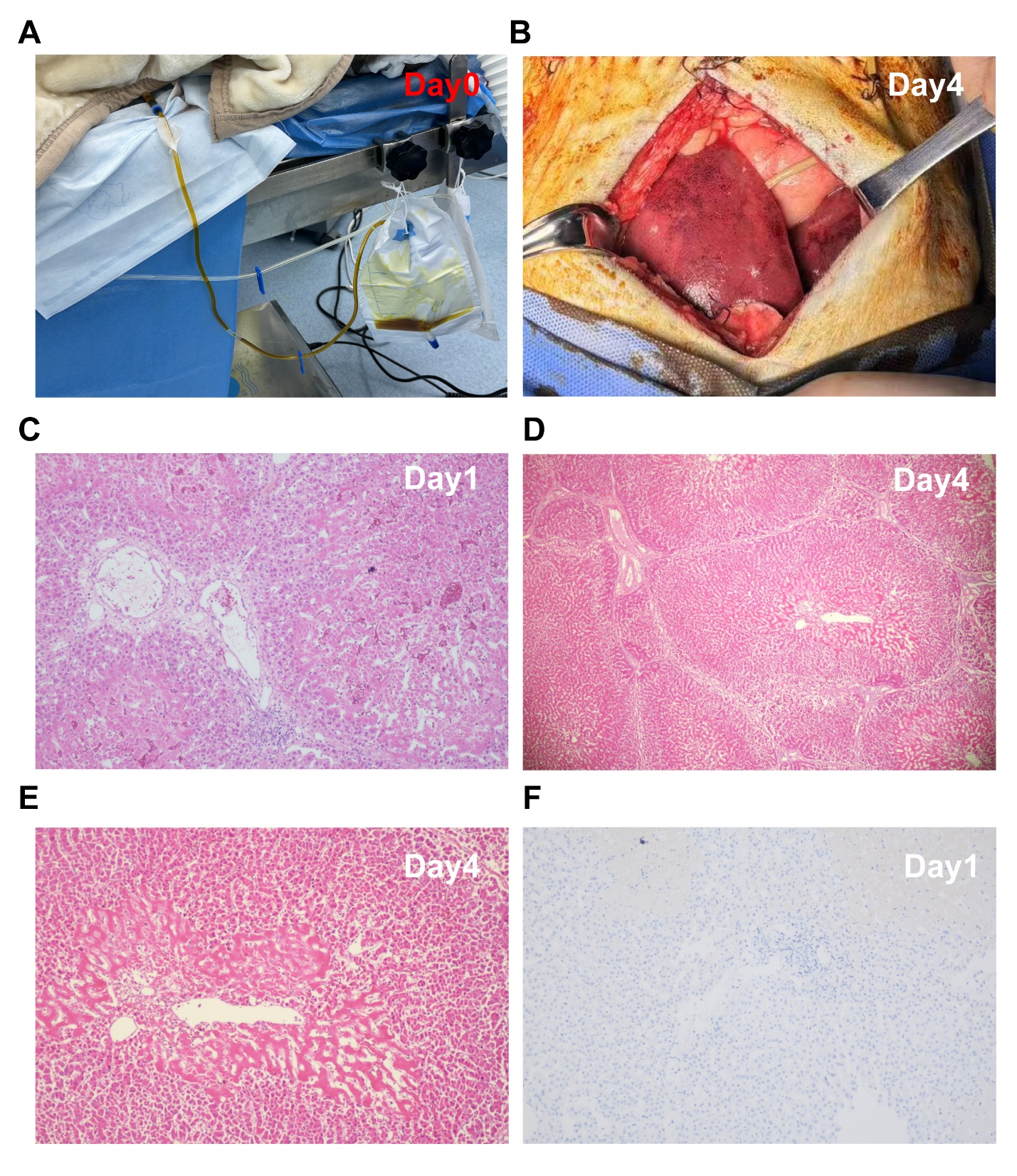


Figure S7


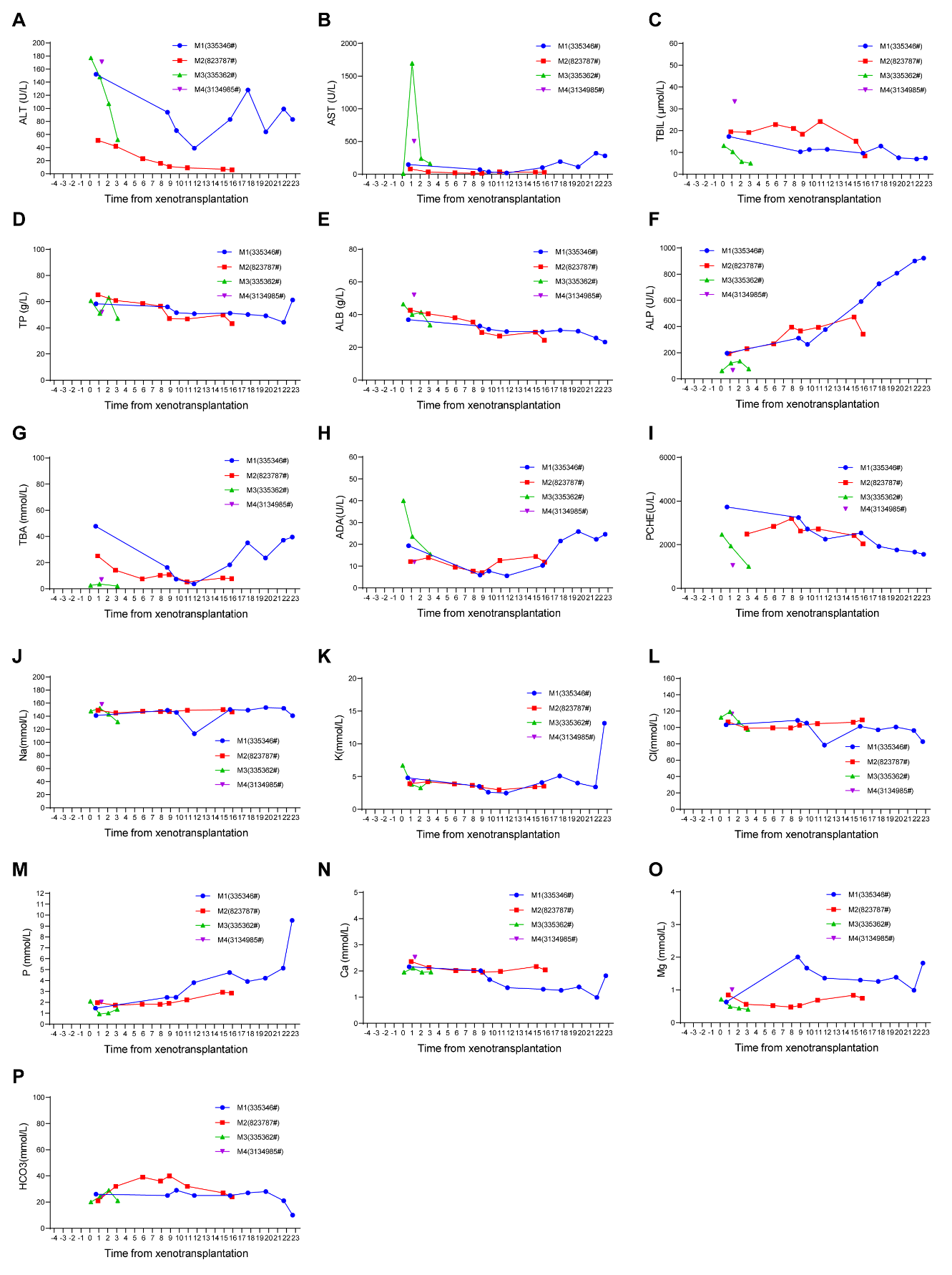


**Supplemental Tables**

Table S1 Primers for genotyping, qPCR and ddPCR

| Primers | Sequence (5’-3’) | Size |
| --- | --- | --- |
| **Genotyping** | | |
| GGTA1E3-F | GGCAACATGGCAGGAAGGAA | 775bp |
| GGTA1E3-R | AGACGGCCCTGTCAGTTCAT |  |
| GGTA1E8-F | CCTGGTTGGCTTCCAAGAGT | 817bp |
| GGTA1E8-R | TTCATCATGCCACTCGGCTT |  |
| CMAH-F | AGCCTTAGAAGCCAGTGCAG | 904bp |
| CMAH-R | GCCCAAGCGAATCTACCTCA |  |
| β4GalNT2-F | TGCTCCATCGCAAGTGAC | 758bp |
| β4GalNT2-R | CCCGCAGTCAGGATTCAC |  |
| hCD55&hCD59-F | TCCAGCACCACCACAAATTGAC | 1124bp |
| hCD55&hCD59-R | CGGTGACCCGCTCGATGTG |  |
| hCD46-F | CTGATGAGACCCACAGAG | 100bp |
| hCD46-F | GCTCCACCATCTGCTTTC |  |
| hTBM&hCD39-F | GCTCGTGCATTCGGGCTTGC | 766bp |
| hTBM&hCD39-R | CCTGGCACCCTGGAAGTCAAAG |  |
| hTBM&hCD39-F | CCTTCCTCAATGCCAGTCAG | 2168bp |
| hTBM&hCD39-R | CCTGGCACCCTGGAAGTCAAAG |  |
| hEPCR-qPCR-F | GCTGAATGATCGTGGTGTTG | 140bp |
| hEPCR-qPCR-R | TGGCCTCCAAAGACTTCAT |  |
| hCD47PCR-F | GATGGATAAGAGTGATGCTGTC | 469bp |
| hCD47PCR-R | TCCAACCACAGCGAGGATATAG |  |
| **qPCR** | | |
| hCD46-F | ccaggtgcaggatcacaact | 146bp |
| hCD46-R | gcaatttggagcggtaagc |  |
| hCD55-F | TCCAGCACCACCACAAATTGAC | 207bp |
| hCD55-R | GGTGGGACCTTGGAAGTTAGAG |  |
| hCD59-F | TCATAGCCTGCAGTGCTACAAC | 103bp |
| hCD59-R | CCCAGCTTTGGTAATGAGACAC |  |
| hTBM-F | CATCCTGGACGACGGTTTCA | 107bp |
| hTBM-R | CGCAGATGCACTCGAAGGTA |  |
| hCD39-F | GGTGCCTATGGCTGGATTA | 211bp |
| hCD39-R | CCTTGCCATAGAGGCGAAA |  |
| hEPCR-qPCR-F | GCTGAATGATCGTGGTGTTG | 140bp |
| hEPCR-qPCR-R | TGGCCTCCAAAGACTTCAT |  |
| hCD47-qPCR-F | TTTCTCATCCATACCACCG | 229bp |
| hCD47-qPCR-R | GATGGATAAGAGTGATGCTGTC |  |
| pGAPDH-F | AGGGCATCCTGGGCTACACT | 367bp |
| pGAPDH-R | TCCACCACCCTGTTGCTGTAG |  |
| hGAPDH-F | GAGTCAACGGATTTGGTCGT | 160bp |
| hGAPDH-R | TGGAAGATGGTGATGGGATT |  |
| **ddPCR** | | |
| hCD46-F | CGTGGTCTCTTCTGCCTATTT | 95bp |
| hCD46-R | AAGGAAACTGAGACGCTACTG |  |
| hCD46 Probe | FAM TCCAGTGAAAGAAGCCAAGATCAGTAAGC |  |
| hCD55-F | GGTGCCAACAAGGCTAAATTC | 99bp |
| hCD55-R | CCTGGACGGCACTCATATTC |  |
| hCD55 Probe | FAM TGCATCCCTCAAACAGCCTTATATCACT |  |
| hCD59-F | CTGCAAGAAGGACCTGTGTAA | 93bp |
| hCD59-R | AATGGAGTCACCAGCAGAAG |  |
| hCD59 Probe | FAM AATGGTGGGACATCCTTATCAGAGAAA |  |
| hTBM-F | ACGTGGATGACTGCATACTG | 100bp |
| hTBM-R | ACCAGGTCGTAGTTAGGGTAG |  |
| hTBM Probe | FAM TCAACACACAGGGTGGCTTCGAG |  |
| hCD39-F | GCTCTGCAATTTCGCCTCTAT | 106bp |
| hCD39-R | GAATGTCCTTGGCCAGTTTCT |  |
| hCD39 Probe | FAM CTTCTTGTGCTATGGGAAGGATCAGGC |  |
| hCD47-F | CAGCGATTGGATTAACCTCCT | 107bp |
| hCD47-R | TGGTATACACGCCGCAATAC |  |
| hCD47 Probe | FAM CCTATATCCTCGCTGTGGTTGGACTGA |  |
| hEPCR-F | CAACCGCACTCGGTATGAA | 97bp |
| hEPCR-R | TTGGCTCCCTTTCGTGTTT |  |
| hEPCR Probe | FAM TTCTGCACATACTGCACACAGGTGTC |  |
| hGAPDH-F | CCTAGGGCTGCTCACATATTC | 85bp |
| hGAPDH-R | CGCCCAATACGACCAAATCTA |  |
| hGAPDH Probe | HEX CTCATGCCTTCTTGCCTCTTGTCTCT |  |
| pGAPDH-F | ccgcgatctaatgttctctttc | 114bp |
| pGAPDH-R | ttcactccgaccttcaccat |  |
| pGAPDH Probe | HEX cagccgcgtccctgagacac |  |

Table S2. Summary of the antibodies used for immunofluorescence, immunohistochemistry and flow cytometry.

|  | Primary antibody | | Secondary antibody | |
| --- | --- | --- | --- | --- |
|  | Catalog | Manufacturer | Catalog | Manufacturer |
| **Immunofluorescence** | | | | |
| GGTA1 | ALX-650-001F-MC05 | Enzo |  |  |
| CMAH(Neu5Gc) | 146903 | BioLegend | 550021 | Zenbio |
| B4GalNT2(DBA) | FL-1031 | Vector Labs |  |  |
| hCD46 | ab108307 | Abcam | GB21303 | Servicebio |
| hCD55 | ab230638 | Abcam | GB21303 | Servicebio |
| hCD55 | ab133684 | Abcam | GB21303 | Servicebio |
| hCD59 | BF0017 | Affinity | GB21301 | Servicebio |
| hCD59 | ab9183 | Abcam | GB21301 | Servicebio |
| hTBM(CD141) | sc-13164 | Santa Cruz | GB21301 | Servicebio |
| hCD39 | ab223842 | Abcam | GB21303 | Servicebio |
| hCD47 | ab226837 | Abcam | GB21303 | Servicebio |
| hEPCR | UM870092 | OriGene | GB21301 | Servicebio |
| anti-CD3e | 85061T | CST | GB21303 | Servicebio |
| anti-CD4 | A22773 | ABClone |  |  |
| anti-CD8 | A23346 | ABClone |  |  |
| IgG | 62-8411 | Invitrogen |  |  |
| IgM | A18842 | Invitrogen |  |  |
| C3c | RAB-0027 | MXB Biotech. | GB21303 | Servicebio |
| C4d | RMA-0857 | MXB Biotech. | GB21303 | Servicebio |
| C5b-C9 | MA5-28502 | Invitrogen | GB21301 | Servicebio |
| **Immunohistochemistry** | | | | |
| CD57 | 14-0577-82 | Invitrogen | PV-9000 | OriGene |
| CD68 | 76437S | CST | PV-9000 | OriGene |
| **Flow cytometry** | | | | |
| GGTA1 | ALX-650-001F-MC05 | Enzo |  |  |
| CMAH(Neu5Gc) | 146903 | BioLegend | 550021 | Zenbio |
| B4GalNT2(DBA) | FL-1031 | Vector Labs |  |  |
| hCD46 | 315304 | Biolegend |  |  |
| hCD55 | 555694 | BD |  |  |
| hCD59 | MA1-19463 | Invitrogen |  |  |
| hTBM | 564123 | BD |  |  |
| hCD39 | 560239 | BD |  |  |
| hCD47 | 556045 | BD |  |  |
| hEPCR | 557950 | BD |  |  |
| **Western Blot** | | | | |
| Antibody | Catalog | Manufacturer | Concentration | |
| hCD55 | DF636141 | Affinity | 1:500 | |
|  | ab133684 | Abcam | 1:2000 | |
| hCD59 | HPA026494 | SIGMA | 1:1000 | |
| hCD46 | HPA016903 | SIGMA | 1:2000 | |
| hTBM | SC-13164 | SANTA Cruz | 1:1000 | |
| hCD39 | ab223842 | Abcam | 1:1000 | |
| β-actin | A5541 | SIGMA | 1:5000 | |
| Secondary antibody | LF102 | Epizyme | 1:5000 | |

**Table S3. Genotyping of colonies for GGTA1, CMAH and β4GalNT2 genes by Sanger sequencing in the first gene editing.**

| **Gene** | **No. of colonies** | **Sequence** | **Mutation** |
| --- | --- | --- | --- |
| GGTA1 | WT | TGATGTATTCCCAAAACACAACCATTACAGTTGAGACAAGCAGCATTGACAGAACCACTCTTCCTTTGACATTCATTATTTTCTCCTGGGAAAAGAAAAG | WT |
|  | C2# | TGATGTATTCCCAAAACACAACCATTACAGTTGAGACAAGCAGCATTGACAGAACCACTCTTCCTT--------CATTATTTTCTCCTGGGAAAAGAAAAG  TGATGTATTCCCAAAACACAACCATTACAGTTGAGACAAGCAGCATTGACAGAACCACTCTTCC-----------ATTATTTTCTCCTGGGAAAAGAAAAG | -7bp 4/6  -1bp 2/6 |
|  | C3# | TGATGTATTCCCAAAACACAACCATTACAGTTGAGACAAGCAGCATTGACAGAACCACTCTTCCTTTGACATTCATTATTTTCTCCTGGGAAAAGAAAAG | WT 1/6 |
|  |  | TGATGTATTCCCAAAACACAACCATTACAGTTGAGACAAGCAGCAT-----------------------------------------------------G | -53bp 2/6 |
|  |  | TGATGTATTCCCAAAACACAACCATTACAGTTGAGACAAGCAGCATTGACAGAACCACTCTTCCTTTGACAAC-------------CCTGGGAAAAGAAAAG | -13bp/+2bp 2/6 |
|  | C5# | TGATGTATTCCCAAAACACAACCATTACAGTTGAGACAAGCAGCATTGACAGAACCACTCTTCCTTTGACATTCATTATTTTCTCCTGGGAAAAGAAAAG | WT 1/13 |
|  |  | TGATGTATTCCCAAAACACAACCATTACAGTTGAGACAAGCAGCATTGACAGAACCACTCTTCCTTTGTG---TTCATTATTTTCTCCTGGGAAAAGAAAAG | -3bp/+2bp 3/13 |
|  |  | TGATGTATTCCCAAAACACAACCATTACAGTTGAGACAAGCAGCATTGACAGAACCACTCTTCCTTT---------TATTTTCTCCTGGGAAAAGAAAAG | -9bp 1/13 |
|  |  | -----------------------------------------------------------------------------------ATTATTTTCTCCTGGGAAAAGAAAAG | -402bp 8/13 |
|  | C6# | TGATGTATTCCCAAAACACAACCATTACAGTTGAGACAAGCAGCATTGACAGAACCACTCTTCCTTTGACATTCATTATTTTCTCCTGGGAAAAGAAAAG | WT 1/4 |
|  |  | TGATGTATTCCCAAAACACAACCATTACAGTTGAGACAAGCAGCATTGACAGAACCACTCTTCCTTTGTG---TTCATTATTTTCTCCTGGGAAAAGAAAAG | -3bp/+2bp 2/4 |
|  |  | TGATGTATTCCCAAAACACAACCATTACAGTTGAGACAAGCAGCATTGACAGAACCACTCTTCCTTT---------TATTTTCTCCTGGGAAAAGAAAAG | -9bp 1/4 |
|  | C10# | TGATGTATTCCCAAAACACAACCATTACAGTTGAGACAAGCAGCATTGACAGAACCACTCTTCCTTTGACATTCATTATTTTCTCCTGGGAAAAGAAAAG | WT 2/8 |
|  |  | TGATGTATTCCCAAAACACAACCATTACAGTTGAGACAAGCAGCATTGACAGAACCACTCTTCCTT--------CATTATTTTCTCCTGGGAAAAGAAAAG | -7bp 2/8 |
|  |  | TGATGTATTCCCAAAACACAACCATTACAGTTGAGACAAGCAGCATTGACAGAACCACTCTTCC-----------ATTATTTTCTCCTGGGAAAAGAAAAG | -10bp 4/8 |
|  | C21# | TGATGTATTCCCAAAACACAACCATTACAGTTGAGACAAGCAGCATTGACAGAACCACTCTTCCTTTGACATTCATTATTTTCTCCTGGGAAAAGAAAAG | WT 6/11 |
|  |  | -----------------------------------------------------------------------------------------TTCTCCTGGGAAAAGAAAAG | -378bp 5/11 |
|  | C24# | TGATGTATTCCCAAAACACAACCATTACAGTTGAGACAAGCAGCATTGACAGAACCACTCTTCCTTTGACATTCATTATTTTCTCCTGGGAAAAGAAAAG | WT 3/7 |
|  |  | TGATGTATTCCCAAAACACAACCATTACAGTTGAGACAAGCAGCATTGACAGAACCACTCTTCCTT--------CATTATTTTCTCCTGGGAAAAGAAAAG | -7bp 2/7 |
|  |  | TGATGTATTCCCAAAACACAACCATTACAGTTGAGACAAGCAGCATTGACAGAACCACTCTTCCTTTGAACATTCATTATTTTCTCCTGGGAAAAGAAAAG | +1bp 2/7 |
|  | C26# | TGATGTATTCCCAAAACACAACCATTACAGTTGAGACAAGCAGCATTGACAGAACCACTCTTCCTTTGACATTCATTATTTTCTCCTGGGAAAAGAAAAG | WT 3/6 |
|  |  | TGATGTATTCCCAAAACACAACCATTACAGTTGAGACAAGCAGCATTGACAGAACCACTCTTCCTTTGA-ATTCATTATTTTCTCCTGGGAAAAGAAAAG | -1bp 3/6 |
|  | C34# | TGATGTATTCCCAAAACACAACCATTACAGTTGAGACAAGCAGCATTGACAGAACCACTCTTCCTTTGACATTCATTATTTTCTCCTGGGAAAAGAAAAG | WT 1/6 |
|  |  | TGATGTATTCCCAAAACACAACCATTACAGTTGAGACAAGCAGCATTGACAGAACCACTCTTCCTTTGA--TTCATTATTTTCTCCTGGGAAAAGAAAAG | -2bp 3/6 |
|  |  | TGATGTATTCCCAAAACACAACCATTACAGTTGAGACAAGCAGCATTGACAGAACCACTCTTCCTTTGAACATTCATTATTTTCTCCTGGGAAAAGAAAAG | **+**1bp 2/6 |
|  | C44# | TGATGTATTCCCAAAACACAACCATTACAGTTGAGACAAGCAGCATTGACAGAACCACTCTTCCTTTGACATTCATTATTTTCTCCTGGGAAAAGAAAAG | WT 5/11 |
|  |  | TGATGTATTCCCAAAA-----------------------------------------------------------ACATTCATTATTTTCTCCTGGGAAAAGAAAAG | -52bp 1/11 |
|  |  | -----------------------------------------------------------------------------------------TTCTCCTGGGAAAAGAAAAG | -380bp 5/11 |
| CMAH | WT | TCACTGTCTTCCAACAGATCACGTACCTTACTCACG(84)TACTACACGAGCCTCCATCTGATTGG(62)GTAAGGAAGGGTGAGCCCTCAACTCCGAAGA | WT |
|  | C2# | TCACTGTCTTCCAACAGATCACGTACCTTACTCACG(84)TACTACACGAGCCTCCATCTGATTGG(62)GTAAGGAAGGGTGAGCCCTCAACTCCGAAGA | WT 3/7 |
|  |  | TCACTGTCTT--------------------------**(84)**-------------------TGATTGG(62)GTAAGGAAGGGTGAGCCCTCAACTCCGAAGA | -123 4/7 |
|  | C3# | TCACTGTCTTCCAACAGATCACGTACCTTACTCACG(84)TACTACACGAGCCTCCATCTGATTGG(62)GTAAGGAAGGGTGAGCCCTCAACTCCGAAGA | WT 7/18 |
|  |  | TCACTGTCTT--------------------------**(84)**-------------------TGATTGG(62)GTAAGGAAGGGTGAGCCCTCAACTCCGAAGA | -123bp 5/18 |
|  |  | TCACTGTCTTCCAACA----------CTTACTCACG(84)TACTACACGAGCCTCCA---------**(62)**---------------------ACTCCGAAGA | 102bp 5/18 |
|  |  | TCACTGTCTTCCAACAGATCACGTACCTTACTCACG(84)TACTACACGAGCCTCCA---------**(62)**---------------------ACTCCGAAGA | 92bp 1/18 |
|  | C10# | TCACTGTCTTCCAACAGATCACGTACCTTACTCACG(84)TACTACACGAGCCTCCATCTGATTGG(62)GTAAGGAAGGGTGAGCCCTCAACTCCGAAGA | WT 2/7 |
|  |  | TCACTGTCTT--------------------------**(84)**-------------------TGATTGG(62)GTAAGGAAGGGTGAGCCCTCAACTCCGAAGA | -123bp 5/7 |
|  | C24# | TCACTGTCTTCCAACAGATCACGTACCTTACTCACG(84)TACTACACGAGCCTCCATCTGATTGG(62)GTAAGGAAGGGTGAGCCCTCAACTCCGAAGA | WT 2/8 |
|  |  | TCACTGTCTT--------------------------**(84)**-------------------TGATTGG(62)GTAAGGAAGGGTGAGCCCTCAACTCCGAAGA | -123bp 6/8 |
| B4GalNT2 | WT | AGCCCTAGATGTCTGTCGATCCTCAAGAT(99)CACCCTGGATGCGCAGACGCTGAAGCTTCTACC(15)CTACGGTGAAAACGGGTGAGATGGCAA | WT |
|  |  | AGCCCTAGATGTCTGTCGATCCTCAAGAT(99)CACCCTGGATGCGCAGACGCTGAAGCTTCTACC(27)CTACAGTGGAATCTGGTGAGATGGCAA | WT |
|  | C2# | AGCCCTAGATGTCTGTCGATCCTCAAGAT(99)CACCCTGGATGCGCAGACGCTGAAGCTTCTACC(27)CTACAGTGGAATCTGGTGAGATGGCAA | WT 1/4 |
|  |  | AGCCCTAGAT-------------------**(99)**---------------------------------**(15)**------------------AGATGGCAA | -184bp 3/4 |
|  | C3# | AGCCCTAGATGTCTGTCGATCCTCAAGAT(99)CACCCTGGATGCGCAGACGCTGAAGCTTCTACC(15)CTACGGTGAAAACGGGTGAGATGGCAA | WT 3/9 |
|  |  | AGCCCTAGATGTCTGTCGATCCTCAAGAT(99)CACCCTGGATGCGCAGACGCTGAAGCTTCTACC(27)CTACAGTGGAATCTGGTGAGATGGCAA | WT 2/9 |
|  |  | AGCCCTAGATG--------------------**(99)**--------------------------------**(15)**-----------------AGATGGCAA | -183bp 2/9 |
|  |  | AGCCCTAGATG------------------**(99)**----------------------AAGCTTCTACC(27)CTACAGTGGAATCTGGTGAGATGGCAA | -139bp 2/9 |
|  | C10# | AGCCCTAGATGTCTGTCGATCCTCAAGAT(99)CACCCTGGATGCGCAGACGCTGAAGCTTCTACC(15)CTACGGTGAAAACGGGTGAGATGGCAA | WT 1/10 |
|  |  | AGCCCTAGATGTCTGTCGATCCTCAAGAT(99)CACCCTGGATGCGCAGACGCTGAAGCTTCTACC(27)CTACAGTGGAATCTGGTGAGATGGCAA | WT 1/10 |
|  |  | AGCCCTAGATG--------------------**(99)**-------------------------------**(15)**-----------------AGATGGCAA | -183bp 4/10 |
|  |  | AGCCCTAGATGTCTGTCGATCCTCAAGAT(99)CACCCTGGAT-----------------------**(15)**-----------------AAGATGGCAA | -61bp 1/10 |
|  |  | AGCCCTAGATGTCTGTCGATCCTCAAGAT(99)CACCCTGGATGCGCAGACGCTGAAGCTTCTACC(15)CTACGGTGAA---------GATGGCAA | -9bp 3/10 |
|  | C12# | AGCCCTAGATGTCTGTCGATCCTCAAGAT(99)CACCCTGGATGCGCAGACGCTGAAGCTTCTACC(15)CTACGGTGAAAACGGGTGAGATGGCAA | WT 2/6 |
|  |  | AGCCCTAGATGTCTGTCGATCCTCAAGAT(99)CACCCTGGATGCGCAGACGCTGAAGCTTCTACC(27)CTACAGTGGAATCTGGTGAGATGGCAA | WT 1/6 |
|  |  | AGCCCTAGATG--------------------**(99)**-------------------------------**(15)**-----------------AGATGGCAA | -183bp 2/6 |
|  |  | AGCCCTAGATGTCTGTCGATCCTCAAGAT(99)CACCCTGGATGCGCAGACGCTGAAGCTTCTACC(15)CTACGGTGAA---------GATGGCAA | -9bp 1/6 |
|  | C21# | AGCCCTAGATGTCTGTCGATCCTCAAGAT(99)CACCCTGGATGCGCAGACGCTGAAGCTTCTACC(15)CTACGGTGAAAACGGGTGAGATGGCAA | WT 2/4 |
|  |  | AGCCCTAGATGTCTGTCGATCCTCAAGAT(99)CACCCTGGATGCGCAGACGCTGAAGCTTCTACC(27)CTACAGTGGAATCTGGTGAGATGGCAA | WT 1/4 |
|  |  | AGCCCTAGATG--------------------**(99)**-------------------------------**(15)**-----------------AGATGGCAA | -183bp 1/4 |
|  | C24# | AGCCCTAGATGTCTGTCGATCCTCAAGAT(99)CACCCTGGATGCGCAGACGCTGAAGCTTCTACC(27)CTACAGTGGAATCTGGTGAGATGGCAA | WT 5/7 |
|  |  | AGCCCTAGATG--------------------**(99)**-------------------------------**(15)**-----------------AGATGGCAA | -183bp 2/7 |

| **Table S4. Summary of genotypes of cell colonies used for the production of 8-GEC male pig** | | | | | | | | | | |
| --- | --- | --- | --- | --- | --- | --- | --- | --- | --- | --- |
| No. | ID | GGTA1 | CMAH | β4GalNT2 | hCD46 | hCD55 | hCD59 | hTBM | hCD39 |  |
| 1 | C2# | +1/△7/△10/+2△9 | WT/△123 | WT/WT/△184/△184 | ○ | ○ | ○ | ○ | ○ |  |
| 2 | C3# | WT/△53/+2△13 | WT/△123/△102/△92 | WT/△139/WT/△183 | ○ | ○ | ○ | ○ | ○ |  |
| 3 | C5# | WT/+2△3/△9/△402 | - | WT | ○ | ○ | ○ | ○ | ○ |  |
| 4 | C6# | WT/△10/+1△3 | - | WT | ○ | ○ | ○ | ○ | ○ |  |
| 5 | C10# | WT/△10/△7 | WT/△123 | WT/WT/△9/+2△59/△184 | ○ | ○ | ○ | ○ | ○ |  |
| 6 | C12# | WT/△10/△7 | - | WT/WT/△9/△184 | ○ | ○ | ○ | ○ | ○ |  |
| 7 | C21# | WT/△378 | - | WT/WT/△182/△182 | ○ | ○ | ○ | ○ | ○ |  |
| 8 | C24# | WT/+1/+11△2/△7 | WT/△123 | WT/△119/△183/△183 | ○ | ○ | ○ | ○ | ○ |  |
| 9 | C26# | WT/△1/△1 | - | WT | ○ | ○ | ○ | ○ | ○ |  |
| 10 | C34# | WT/+2/△1 | - | WT | ○ | ○ | ○ | ○ | ○ |  |
| 11 | C44# | WT/△52/△380 | - | WT | ○ | ○ | ○ | ○ | ○ |  |
| 12 | C4# | WT | - | - | ○ | ○ | ○ | ○ | ○ |  |
| 13 | C8# | WT | - | - | ○ | ○ | ○ | ○ | ○ |  |
| 14 | C9# | WT | - | - | ○ | ○ | ○ | ○ | ○ |  |
| 15 | C11# | WT | - | - | ○ | ○ | ○ | ○ | ○ |  |
| 16 | C13# | WT | - | - | ○ | ○ | ○ | ○ | ○ |  |
| 17 | C14# | WT | - | - | ○ | ○ | ○ | ○ | ○ |  |
| 18 | C15# | WT | - | - | ○ | ○ | ○ | ○ | ○ |  |
| 19 | C17# | WT | - | - | ○ | ○ | ○ | ○ | ○ |  |
| 20 | C18# | WT | - | - | ○ | ○ | ○ | ○ | ○ |  |
| 21 | C19# | WT | - | - | ○ | ○ | ○ | ○ | ○ |  |
| 22 | C20# | WT | - | - | ○ | ○ | ○ | ○ | ○ |  |
| 23 | C22# | WT | - | - | ○ | ○ | ○ | ○ | ○ |  |
| 24 | C27# | WT | - | - | ○ | ○ | ○ | ○ | ○ |  |
| 25 | C28# | WT | - | - | ○ | ○ | ○ | ○ | ○ |  |
| 26 | C29# | WT | - | - | ○ | ○ | ○ | ○ | ○ |  |
| 27 | C31# | WT | - | - | ○ | ○ | ○ | ○ | ○ |  |
| 28 | C32# | WT | - | - | ○ | ○ | ○ | ○ | ○ |  |
| 29 | C33# | WT | - | - | ○ | ○ | ○ | ○ | ○ |  |
| 30 | C35# | WT | - | - | ○ | ○ | ○ | ○ | ○ |  |
| 31 | C39# | WT | - | - | ○ | ○ | ○ | ○ | ○ |  |
| 32 | C48# | Sequencing Defeat | - | - | ○ | ○ | ○ | ○ | ○ |  |
| 33 | Remaining 17 colonies | - | - | - | × | × | × | × | × |  |
| Note: A total of 49 cell colonies were screened, among which C2, C3, C4, C5, C6, C8, C9, C10, C11, C12, C13, C14, C15, C17, C18, C19, C20, C21, C22, C24, C26, C27, C28, C29, C31, C32, C33, C34, C35, C39, C44, C48 (a total of 32) were PCR-positive cell colonies, indicating successful insertion of hTBM/hCD39/hCD46/hCD55/hCD59. These colonies were used for GGTA1 gene target fragment PCR and Sanger sequencing, and the mutated colonies are shown in the table. Then, β4GalNT2 gene target fragment PCR and Sanger sequencing were performed, and the mutated colonies were C2, C3, C10, C12, C21, and C24 (a total of 6). Among them, C12 colony was a single knockout and existed in multiples of 3 (△9), and C21 clone was confirmed to be a single knockout for both GGTA1 and beta4GalNT2. Finally, Sanger sequencing was performed on the CMAH gene target fragment for C2, C3, C10, and C24 clones (a total of 4). (“-”: not tested; “○”: insertion detected; “×”: no insertion detected) | | | | | | | | | | |

**Table S5. Genotyping the GGTA1, CMAH and β4GalNT2 genes of 8-GEC fetuses by Sanger sequencing**

| **Gene** | **No. of pigs** | **Sequence** | **Mutation** |
| --- | --- | --- | --- |
| GGTA1  On exon 3 | WT | TGATGTATTCCCAAAACACAACCATTACAGTTGAGACAAGCAGCATTGACAGAACCACTCTTCCTTTGACATTCATTATTTTCTCCTGGGAAAAGAAAAG | WT |
|  | C3F01 | TGATGTATTCCCAAAACACAACCATTACAGTTGAGACAAGCAGCATTGACAGAACCACTCTTCCTTTGACAAC-------------CCTGGGAAAAGAAAAG | -13bp/+2bp 4/10 |
|  |  | TGATGTATTCCCAAAACACAACCATTACAGTTGAGACAAGCAGCAT-----------------------------------------------------G | -53bp 6/10 |
|  | C3F02 | TGATGTATTCCCAAAACACAACCATTACAGTTGAGACAAGCAGCATTGACAGAACCACTCTTCCTTTGACAAC-------------CCTGGGAAAAGAAAAG | -13bp/+2bp 5/8 |
|  |  | TGATGTATTCCCAAAACACAACCATTACAGTTGAGACAAGCAGCAT-----------------------------------------------------G | -53bp 3/8 |
|  | C3F03 | TGATGTATTCCCAAAACACAACCATTACAGTTGAGACAAGCAGCATTGACAGAACCACTCTTCCTTTGACAAC-------------CCTGGGAAAAGAAAAG | -13bp/+2bp 4/6 |
|  |  | TGATGTATTCCCAAAACACAACCATTACAGTTGAGACAAGCAGCAT-----------------------------------------------------G | -53bp 2/6 |
|  | C3F04 | TGATGTATTCCCAAAACACAACCATTACAGTTGAGACAAGCAGCATTGACAGAACCACTCTTCCTTTGACAAC-------------CCTGGGAAAAGAAAAG | -13bp/+2bp 4/7 |
|  |  | TGATGTATTCCCAAAACACAACCATTACAGTTGAGACAAGCAGCAT-----------------------------------------------------G | -53bp 3/7 |
|  | C3F05 | TGATGTATTCCCAAAACACAACCATTACAGTTGAGACAAGCAGCATTGACAGAACCACTCTTCCTTTGACAAC-------------CCTGGGAAAAGAAAAG | -13bp/+2bp 10/15 |
|  |  | TGATGTATTCCCAAAACACAACCATTACAGTTGAGACAAGCAGCAT-----------------------------------------------------G | -53bp 5/15 |
|  | C3F06 | TGATGTATTCCCAAAACACAACCATTACAGTTGAGACAAGCAGCATTGACAGAACCACTCTTCCTTTGACAAC-------------CCTGGGAAAAGAAAAG | -13bp/+2bp 3/7 |
|  |  | TGATGTATTCCCAAAACACAACCATTACAGTTGAGACAAGCAGCAT-----------------------------------------------------G | -53bp 4/7 |
|  | C3F07 | TGATGTATTCCCAAAACACAACCATTACAGTTGAGACAAGCAGCATTGACAGAACCACTCTTCCTTTGACAAC-------------CCTGGGAAAAGAAAAG | -13bp/+2bp 3/9 |
|  |  | TGATGTATTCCCAAAACACAACCATTACAGTTGAGACAAGCAGCAT-----------------------------------------------------G | -53bp 6/9 |
|  | C3F08 | TGATGTATTCCCAAAACACAACCATTACAGTTGAGACAAGCAGCATTGACAGAACCACTCTTCCTTTGACAAC-------------CCTGGGAAAAGAAAAG | -13bp/+2bp 5/9 |
|  |  | TGATGTATTCCCAAAACACAACCATTACAGTTGAGACAAGCAGCAT-----------------------------------------------------G | -53bp 4/9 |
|  | C3F09 | TGATGTATTCCCAAAACACAACCATTACAGTTGAGACAAGCAGCATTGACAGAACCACTCTTCCTTTGACAAC-------------CCTGGGAAAAGAAAAG | -13bp/+2bp 8/11 |
|  |  | TGATGTATTCCCAAAACACAACCATTACAGTTGAGACAAGCAGCAT-----------------------------------------------------G | -53bp 11/11 |
|  | C3F10 | TGATGTATTCCCAAAACACAACCATTACAGTTGAGACAAGCAGCATTGACAGAACCACTCTTCCTTTGACAAC-------------CCTGGGAAAAGAAAAG | -13bp/+2bp 3/8 |
|  |  | TGATGTATTCCCAAAACACAACCATTACAGTTGAGACAAGCAGCAT-----------------------------------------------------G | -53bp 5/8 |
|  | C3F11 | TGATGTATTCCCAAAACACAACCATTACAGTTGAGACAAGCAGCATTGACAGAACCACTCTTCCTTTGACAAC-------------CCTGGGAAAAGAAAAG | -13bp/+2bp 8/9 |
|  |  | TGATGTATTCCCAAAACACAACCATTACAGTTGAGACAAGCAGCAT-----------------------------------------------------G | -53bp 1/9 |
|  | C3F12 | TGATGTATTCCCAAAACACAACCATTACAGTTGAGACAAGCAGCATTGACAGAACCACTCTTCCTTTGACAAC-------------CCTGGGAAAAGAAAAG | -13bp/+2bp 5/10 |
|  |  | TGATGTATTCCCAAAACACAACCATTACAGTTGAGACAAGCAGCAT-----------------------------------------------------G | -53bp 5/10 |
|  | C3F13 | TGATGTATTCCCAAAACACAACCATTACAGTTGAGACAAGCAGCATTGACAGAACCACTCTTCCTTTGACAAC-------------CCTGGGAAAAGAAAAG | -13bp/+2bp 6/9 |
|  |  | TGATGTATTCCCAAAACACAACCATTACAGTTGAGACAAGCAGCAT-----------------------------------------------------G | -53bp 3/9 |
| CMAH | WT | TCACTGTCTTCCAACAGATCACGTACCTTACTCACG(84)TACTACACGAGCCTCCATCTGATTGG(62)GTAAGGAAGGGTGAGCCCTCAACTCCGAAGA | WT |
|  | C3F01 | TCACTGTCTT--------------------------**(84)**-------------------TGATTGG(62)GTAAGGAAGGGTGAGCCCTCAACTCCGAAGA | -123bp 6/9 |
|  |  | TCACTGTCTTCCAACA----------CTTACTCACG(84)TACTACACGAGCCTCCA---------**(62)**---------------------ACTCCGAAGA | -102bp 3/9 |
|  | C3F02 | TCACTGTCTT--------------------------**(84)**-------------------TGATTGG(62)GTAAGGAAGGGTGAGCCCTCAACTCCGAAGA | -123bp 6/8 |
|  |  | TCACTGTCTTCCAACA----------CTTACTCACG(84)TACTACACGAGCCTCCA---------**(62)**---------------------ACTCCGAAGA | -102b 2/8 |
|  | C3F03 | TCACTGTCTT--------------------------**(84)**-------------------TGATTGG(62)GTAAGGAAGGGTGAGCCCTCAACTCCGAAGA | -123bp 3/8 |
|  |  | TCACTGTCTTCCAACA----------CTTACTCACG(84)TACTACACGAGCCTCCA---------**(62)**---------------------ACTCCGAAGA | -102b 5/8 |
|  | C3F04 | TCACTGTCTT--------------------------**(84)**-------------------TGATTGG(62)GTAAGGAAGGGTGAGCCCTCAACTCCGAAGA | -123bp 2/8 |
|  |  | TCACTGTCTTCCAACA----------CTTACTCACG(84)TACTACACGAGCCTCCA---------**(62)**---------------------ACTCCGAAGA | -102b 6/8 |
|  | C3F05 | TCACTGTCTT--------------------------**(84)**-------------------TGATTGG(62)GTAAGGAAGGGTGAGCCCTCAACTCCGAAGA | -123bp 3/10 |
|  |  | TCACTGTCTTCCAACA----------CTTACTCACG(84)TACTACACGAGCCTCCA---------**(62)**---------------------ACTCCGAAGA | -102bp 7/10 |
|  | C3F06 | TCACTGTCTT--------------------------**(84)**-------------------TGATTGG(62)GTAAGGAAGGGTGAGCCCTCAACTCCGAAGA | -123bp 7/11 |
|  |  | TCACTGTCTTCCAACA----------CTTACTCACG(84)TACTACACGAGCCTCCA---------**(62)**---------------------ACTCCGAAGA | -102bp 4/11 |
|  | C3F07 | TCACTGTCTT--------------------------**(84)**-------------------TGATTGG(62)GTAAGGAAGGGTGAGCCCTCAACTCCGAAGA | -123bp 6/11 |
|  |  | TCACTGTCTTCCAACA----------CTTACTCACG(84)TACTACACGAGCCTCCA---------**(62)**---------------------ACTCCGAAGA | -102bp 6/11 |
|  | C3F08 | TCACTGTCTT--------------------------**(84)**-------------------TGATTGG(62)GTAAGGAAGGGTGAGCCCTCAACTCCGAAGA | -123bp 7/11 |
|  |  | TCACTGTCTTCCAACA----------CTTACTCACG(84)TACTACACGAGCCTCCA---------**(62)**---------------------ACTCCGAAGA | -102bp 4/11 |
|  | C3F09 | TCACTGTCTT--------------------------**(84)**-------------------TGATTGG(62)GTAAGGAAGGGTGAGCCCTCAACTCCGAAGA | -123bp 5/8 |
|  |  | TCACTGTCTTCCAACA----------CTTACTCACG(84)TACTACACGAGCCTCCA---------**(62)**---------------------ACTCCGAAGA | -102bp 3/8 |
|  | C3F10 | TCACTGTCTT--------------------------**(84)**-------------------TGATTGG(62)GTAAGGAAGGGTGAGCCCTCAACTCCGAAGA | -123bp 5/10 |
|  |  | TCACTGTCTTCCAACA----------CTTACTCACG(84)TACTACACGAGCCTCCA---------**(62)**---------------------ACTCCGAAGA | -102bp 5/10 |
|  | C3F11 | TCACTGTCTT--------------------------**(84)**-------------------TGATTGG(62)GTAAGGAAGGGTGAGCCCTCAACTCCGAAGA | -123bp 6/12 |
|  |  | TCACTGTCTTCCAACA----------CTTACTCACG(84)TACTACACGAGCCTCCA---------**(62)**---------------------ACTCCGAAGA | -102bp 6/12 |
|  | C3F12 | TCACTGTCTT--------------------------**(84)**-------------------TGATTGG(62)GTAAGGAAGGGTGAGCCCTCAACTCCGAAGA | -123bp 5/8 |
|  |  | TCACTGTCTTCCAACA----------CTTACTCACG(84)TACTACACGAGCCTCCA---------**(62)**---------------------ACTCCGAAGA | -102bp 3/8 |
|  | C3F13 | TCACTGTCTT--------------------------**(84)**-------------------TGATTGG(62)GTAAGGAAGGGTGAGCCCTCAACTCCGAAGA | -123bp 5/11 |
|  |  | TCACTGTCTTCCAACA----------CTTACTCACG(84)TACTACACGAGCCTCCA---------**(62)**---------------------ACTCCGAAGA | -102bp 6/11 |
| β4GalNT2 | WT | AGCCCTAGATGTCTGTCGATCCTCAAGAT(99)CACCCTGGATGCGCAGACGCTGAAGCTTCTACC(15)CTACGGTGAAAACGGGTGAGATGGCAA | WT |
|  |  | AGCCCTAGATGTCTGTCGATCCTCAAGAT(99)CACCCTGGATGCGCAGACGCTGAAGCTTCTACC(27)CTACAGTGGAATCTGGTGAGATGGCAA | WT |
|  | C3F01 | AGCCCTAGATG--------------------**(99)**--------------------------------**(15)**-----------------AGATGGCAA | -183bp 6/11 |
|  |  | AGCCCTAGATG------------------**(99)**----------------------AAGCTTCTACC(27)CTACAGTGGAATCTGGTGAGATGGCAA | -139bp 5/11 |
|  | C3F02 | AGCCCTAGATG--------------------**(99)**--------------------------------**(15)**-----------------AGATGGCAA | -183bp 2/9 |
|  |  | AGCCCTAGATG------------------**(99)**----------------------AAGCTTCTACC(27)CTACAGTGGAATCTGGTGAGATGGCAA | -139bp 7/9 |
|  | C3F03 | AGCCCTAGATG--------------------**(99)**--------------------------------**(15)**-----------------AGATGGCAA | -183bp 3/7 |
|  |  | AGCCCTAGATG------------------**(99)**----------------------AAGCTTCTACC(27)CTACAGTGGAATCTGGTGAGATGGCAA | -139bp 4/7 |
|  | C3F04 | AGCCCTAGATG--------------------**(99)**--------------------------------**(15)**-----------------AGATGGCAA | -183bp 2/5 |
|  |  | AGCCCTAGATG------------------**(99)**----------------------AAGCTTCTACC(27)CTACAGTGGAATCTGGTGAGATGGCAA | -139bp 3/5 |
|  | C3F05 | AGCCCTAGATG--------------------**(99)**--------------------------------**(15)**-----------------AGATGGCAA | -183bp 4/7 |
|  |  | AGCCCTAGATG------------------**(99)**----------------------AAGCTTCTACC(27)CTACAGTGGAATCTGGTGAGATGGCAA | -139bp 3/7 |
|  | C3F06 | AGCCCTAGATG--------------------**(99)**--------------------------------**(15)**-----------------AGATGGCAA | -183bp 4/7 |
|  |  | AGCCCTAGATG------------------**(99)**----------------------AAGCTTCTACC(27)CTACAGTGGAATCTGGTGAGATGGCAA | -139bp 3/7 |
|  | C3F07 | AGCCCTAGATG--------------------**(99)**--------------------------------**(15)**-----------------AGATGGCAA | -183bp 5/9 |
|  |  | AGCCCTAGATG------------------**(99)**----------------------AAGCTTCTACC(27)CTACAGTGGAATCTGGTGAGATGGCAA | -139bp 4/9 |
|  | C3F08 | AGCCCTAGATG--------------------**(99)**--------------------------------**(15)**-----------------AGATGGCAA | -183bp 6/11 |
|  |  | AGCCCTAGATG------------------**(99)**----------------------AAGCTTCTACC(27)CTACAGTGGAATCTGGTGAGATGGCAA | -139bp 5/11 |
|  | C3F09 | AGCCCTAGATG--------------------**(99)**--------------------------------**(15)**-----------------AGATGGCAA | -183bp 5/9 |
|  |  | AGCCCTAGATG------------------**(99)**----------------------AAGCTTCTACC(27)CTACAGTGGAATCTGGTGAGATGGCAA | -139bp 4/9 |
|  | C3F10 | AGCCCTAGATG--------------------**(99)**--------------------------------**(15)**-----------------AGATGGCAA | -183bp 6/11 |
|  |  | AGCCCTAGATG------------------**(99)**----------------------AAGCTTCTACC(27)CTACAGTGGAATCTGGTGAGATGGCAA | -139bp 5/11 |
|  | C3F11 | AGCCCTAGATG--------------------**(99)**--------------------------------**(15)**-----------------AGATGGCAA | -183bp 4/8 |
|  |  | AGCCCTAGATG------------------**(99)**----------------------AAGCTTCTACC(27)CTACAGTGGAATCTGGTGAGATGGCAA | -139bp 4/8 |
|  | C3F12 | AGCCCTAGATG--------------------**(99)**--------------------------------**(15)**-----------------AGATGGCAA | -183bp 5/10 |
|  |  | AGCCCTAGATG------------------**(99)**----------------------AAGCTTCTACC(27)CTACAGTGGAATCTGGTGAGATGGCAA | -139bp 5/10 |
|  | C3F13 | AGCCCTAGATG--------------------**(99)**--------------------------------**(15)**-----------------AGATGGCAA | -183bp 7/9 |
|  |  | AGCCCTAGATG------------------**(99)**----------------------AAGCTTCTACC(27)CTACAGTGGAATCTGGTGAGATGGCAA | -139bp 2/9 |

**Table S6. Generation of 8-GEC pigs from cloned fetuses**

| No. | ID | Body weight (g) | Status at birth | Survival time (day) | Surrogate mother |
| --- | --- | --- | --- | --- | --- |
| 1 | 33dDN-8-GEC3F01P01 | 100 | mummy | 0 | P754 |
| 2 | 33dDN-8-GEC3GF01P02 | 1000 | healthy | 130 |  |
| 3 | 33dDN-8-GEC3F01P03 | 1050 | healthy | - |  |
| 4 | 33dDN-8-GEC3F01P04 | 600 | stillbirth | 0 |  |
| 5 | 33dDN-8-GEC3F01P05 | 1120 | stillbirth | 0 |  |
| 6 | 33dDN-8-GEC3F01P06 | 500 | healthy | 3 | P774 |
| 7 | 33dDN8-GEC3F03P01 | 850 | healthy | 5 | P814 |
| 8 | 33dDN8-GEC3F03P02 | 800 | healthy | 3 |  |
| 9 | 33dDN8-GEC3F03P03 | 500 | stillbirth | 0 |  |
| 10 | 33dDN8-GEC3F03P04 | 850 | healthy | 9 |  |
| 11 | 33dDN8-GEC3F03P05 | 800 | healthy | 3 |  |
| 12 | 33dDN8-GEC3F03P06 | 700 | stillbirth | 0 |  |
| 13 | 33dDN8-GEC3F03P07 | 850 | stillbirth | 0 |  |
| 14 | 33dDN8-GEC3F03P08 | 650 | healthy | 96 | P738 |
| 15 | 33dDN8-GEC3F03P09 | 800 | healthy | - |  |
| 16 | 33dDN8-GEC3F03P10 | 850 | healthy | 89 |  |
| 17 | 33dDN8-GEC3F03P11 | 650 | healthy | 55 |  |
| 18 | 33dDN8-GEC3F07P08 | 500 | healthy | 2 | P832 |
| 19 | 33dDN8-GEC3F07P09 | 600 | healthy | - |  |
| 20 | 33dDN8-GEC3F07P10 | 450 | stillbirth | 0 |  |
| 21 | 33dDN8-GEC3F07P11 | 600 | stillbirth | 0 |  |
| 22 | 33dDN8-GEC3F07P12 | 400 | stillbirth | 0 |  |
| 23 | 33dDN8-GEC3F07P13 | 450 | stillbirth | 0 |  |
| 24 | 33dDN8-GEC3F07P14 | 500 | stillbirth | 0 |  |
| 25 | 33dDN8-GEC3F07P15 | 700 | stillbirth | 0 |  |
| 26 | 33dDN8-GEC3F07P16 | 250 | mummy | 0 |  |
| 27 | 33dDN8-GEC3F07P01 | 650 | healthy | 2 | P856 |
| 28 | 33dDN8-GEC3F07P02 | 750 | healthy | 2 |  |
| 29 | 33dDN8-GEC3F07P03 | 800 | healthy | 7 |  |
| 30 | 33dDN8-GEC3F07P04 | 200 | mummy | 0 | P757 |
| 31 | 33dDN8-GEC3F07P05 | 250 | stillbirth | 0 |  |
| 32 | 33dDN8-GEC3F07P06 | 400 | stillbirth | 0 |  |
| 33 | 33dDN8-GEC3F07P07 | 240 | mummy | 0 |  |
| 34 | 33dDN8-GEC3F07P17 | 600 | stillbirth | 0 |  |
| 35 | 33dDN8-GEC3F07P18 | 300 | mummy | 0 |  |
| 36 | 33dDN8-GEC3F07P19 | 950 | healthy | - | P697 |
| 37 | 33dDN8-GEC3F07P20 | 1050 | healthy | - |  |
| 38 | 33dDN8-GEC3F07P21 | 200 | mummy | 0 |  |

**Table S7.** **Sanger sequencing for the genotyping of 3 xenoantigens in 8-GEC pigs generated by recloning using fetal cells**

| **Gene** | **No. of pigs** | **Sequence** | **Mutation** |
| --- | --- | --- | --- |
| GGTA1  on exon 3 | WT | TGATGTATTCCCAAAACACAACCATTACAGTTGAGACAAGCAGCATTGACAGAACCACTCTTCCTTTGACATTCATTATTTTCTCCTGGGAAAAGAAAAG | WT |
|  | F01P02 | TGATGTATTCCCAAAACACAACCATTACAGTTGAGACAAGCAGCATTGACAGAACCACTCTTCCTTTGACAAC-------------CCTGGGAAAAGAAAAG | -13bp/+2bp 3/8 |
|  |  | TGATGTATTCCCAAAACACAACCATTACAGTTGAGACAAGCAGCAT-----------------------------------------------------G | -53bp 5/8 |
|  | F03P01 | TGATGTATTCCCAAAACACAACCATTACAGTTGAGACAAGCAGCATTGACAGAACCACTCTTCCTTTGACAAC-------------CCTGGGAAAAGAAAAG | -13bp/+2bp 6/9 |
|  |  | TGATGTATTCCCAAAACACAACCATTACAGTTGAGACAAGCAGCAT-----------------------------------------------------G | -53bp 3/9 |
|  | F03P02 | TGATGTATTCCCAAAACACAACCATTACAGTTGAGACAAGCAGCATTGACAGAACCACTCTTCCTTTGACAAC-------------CCTGGGAAAAGAAAAG | -13bp/+2bp 4/5 |
|  |  | TGATGTATTCCCAAAACACAACCATTACAGTTGAGACAAGCAGCAT-----------------------------------------------------G | -53bp 1/5 |
|  | F03P10 | TGATGTATTCCCAAAACACAACCATTACAGTTGAGACAAGCAGCATTGACAGAACCACTCTTCCTTTGACAAC-------------CCTGGGAAAAGAAAAG | -13bp/+2bp 3/10 |
|  |  | TGATGTATTCCCAAAACACAACCATTACAGTTGAGACAAGCAGCAT-----------------------------------------------------G | -53bp 7/10 |
|  | F07P03 | TGATGTATTCCCAAAACACAACCATTACAGTTGAGACAAGCAGCATTGACAGAACCACTCTTCCTTTGACAAC-------------CCTGGGAAAAGAAAAG | -13bp/+2bp 3/9 |
|  |  | TGATGTATTCCCAAAACACAACCATTACAGTTGAGACAAGCAGCAT-----------------------------------------------------G | -53bp 6/9 |
| CMAH | WT | TCACTGTCTTCCAACAGATCACGTACCTTACTCACG(84)TACTACACGAGCCTCCATCTGATTGG(62)GTAAGGAAGGGTGAGCCCTCAACTCCGAAGA | WT |
|  | F01P02 | TCACTGTCTT--------------------------**(84)**-------------------TGATTGG(62)GTAAGGAAGGGTGAGCCCTCAACTCCGAAGA | -123bp 5/10 |
|  |  | TCACTGTCTTCCAACAGATCACGTACCTTACTCACG(84)TACTACACGAGCCTCCA---------**(62)**---------------------ACTCCGAAGA | -102bp 5/10 |
|  | F03P01 | TCACTGTCTT--------------------------**(84)**-------------------TGATTGG(62)GTAAGGAAGGGTGAGCCCTCAACTCCGAAGA | -123bp 6/9 |
|  |  | TCACTGTCTTCCAACAGATCACGTACCTTACTCACG(84)TACTACACGAGCCTCCA---------**(62)**---------------------ACTCCGAAGA | -102bp 3/9 |
|  | F03P02 | TCACTGTCTT--------------------------**(84)**-------------------TGATTGG(62)GTAAGGAAGGGTGAGCCCTCAACTCCGAAGA | -123bp 5/8 |
|  |  | TCACTGTCTTCCAACAGATCACGTACCTTACTCACG(84)TACTACACGAGCCTCCA---------**(62)**---------------------ACTCCGAAGA | -102bp 3/8 |
|  | F03P10 | TCACTGTCTT--------------------------**(84)**-------------------TGATTGG(62)GTAAGGAAGGGTGAGCCCTCAACTCCGAAGA | -123bp 2/4 |
|  |  | TCACTGTCTTCCAACAGATCACGTACCTTACTCACG(84)TACTACACGAGCCTCCA---------**(62)**---------------------ACTCCGAAGA | -102bp 7/4 |
|  | F07P03 | TCACTGTCTT--------------------------**(84)**-------------------TGATTGG(62)GTAAGGAAGGGTGAGCCCTCAACTCCGAAGA | -123bp 4/10 |
|  |  | TCACTGTCTTCCAACAGATCACGTACCTTACTCACG(84)TACTACACGAGCCTCCA---------**(62)**---------------------ACTCCGAAGA | -102bp 6/10 |
| β4GalNT2 | WT | AGCCCTAGATGTCTGTCGATCCTCAAGAT(99)CACCCTGGATGCGCAGACGCTGAAGCTTCTACC(15)CTACGGTGAAAACGGGTGAGATGGCAA | WT |
|  |  | AGCCCTAGATGTCTGTCGATCCTCAAGAT(99)CACCCTGGATGCGCAGACGCTGAAGCTTCTACC(27)CTACAGTGGAATCTGGTGAGATGGCAA | WT |
|  | F01P02 | AGCCCTAGATG--------------------**(99)**--------------------------------**(15)**-----------------AGATGGCAA | -183bp 2/9 |
|  |  | AGCCCTAGATG------------------**(99)**----------------------AAGCTTCTACC(27)CTACAGTGGAATCTGGTGAGATGGCAA | -139bp 7/9 |
|  | F03P01 | AGCCCTAGATG--------------------**(99)**--------------------------------**(15)**-----------------AGATGGCAA | -183bp 3/9 |
|  |  | AGCCCTAGATG------------------**(99)**----------------------AAGCTTCTACC(27)CTACAGTGGAATCTGGTGAGATGGCAA | -139bp 6/9 |
|  | F03P02 | AGCCCTAGATG--------------------**(99)**--------------------------------**(15)**-----------------AGATGGCAA | -183bp 3/5 |
|  |  | AGCCCTAGATG------------------**(99)**----------------------AAGCTTCTACC(27)CTACAGTGGAATCTGGTGAGATGGCAA | -139bp 2/5 |
|  | F03P10 | AGCCCTAGATG--------------------**(99)**--------------------------------**(15)**-----------------AGATGGCAA | -183bp 2/7 |
|  |  | AGCCCTAGATG------------------**(99)**----------------------AAGCTTCTACC(27)CTACAGTGGAATCTGGTGAGATGGCAA | -139bp 5/7 |
|  | F07P03 | AGCCCTAGATG--------------------**(99)**--------------------------------**(15)**-----------------AGATGGCAA | -183bp 5/16 |
|  |  | AGCCCTAGATG------------------**(99)**----------------------AAGCTTCTACC(27)CTACAGTGGAATCTGGTGAGATGGCAA | -139bp 11/16 |

Note：F01P02, 8-GEC3F01P02; F03P01, 8-GEC3F03P01; F03P02, 8-GEC3F03P02; F03P10, 8-GEC3F03P10; F07P03, 8-GEC3F07P03

**Table S8. Generation of 8-GEC pigs**

| No. | ID | Body weight (g) | Status at birth | Survival time (day) | Surrogate mother |
| --- | --- | --- | --- | --- | --- |
| 1 | DN-8-GE-C3P01 | 600 | healthy | 448 | P668 |
| 2 | DN-8-GE-C3P02 | 600 | stillbirth | 0 |  |
| 3 | DN-8-GE-C3P03 | 850 | healthy | - |  |
| 4 | DN-8-GE-C3P04 | 550 | stillbirth | 0 |  |
| 5 | DN-8-GE-C3P05 | 550 | healthy | 5 | P679 |
| 6 | DN-8-GE-C3P06 | 600 | stillbirth | 0 |  |
| 7 | DN-8-GE-C3P07 | - | mummy | 0 | P585 |
| 8 | DN-8-GE-C3P08 | 750 | healthy | 5 |  |
| 9 | DN-8-GE-C3P09 | 700 | healthy | 479 |  |
| 10 | DN-8-GE-C3P10 | 800 | healthy | 4 |  |
| 11 | DN-8-GE-C3P11 | 1010 | healthy | - |  |
| 12 | DN-8-GE-C3P12 | 900 | stillbirth | 0 |  |
| 13 | DN-8-GE-C3P13 | 800 | healthy | - |  |

**Table S9. Sanger sequencing for the genotyping of 3 xenoantigens in 8-GEC pigs generated by cloning using cell line**

| **Gene** | **No. of pigs** | **Sequence** | **Mutation** |
| --- | --- | --- | --- |
| GGTA1  on exon 3 | WT | TGATGTATTCCCAAAACACAACCATTACAGTTGAGACAAGCAGCATTGACAGAACCACTCTTCCTTTGACATTCATTATTTTCTCCTGGGAAAAGAAAAG | WT |
|  | P01 | TGATGTATTCCCAAAACACAACCATTACAGTTGAGACAAGCAGCATTGACAGAACCACTCTTCCTTTGACAAC-------------CCTGGGAAAAGAAAAG | -13bp/+2bp 3/6 |
|  |  | TGATGTATTCCCAAAACACAACCATTACAGTTGAGACAAGCAGCAT-----------------------------------------------------G | -53bp 3/6 |
|  | P02 | TGATGTATTCCCAAAACACAACCATTACAGTTGAGACAAGCAGCATTGACAGAACCACTCTTCCTTTGACAAC-------------CCTGGGAAAAGAAAAG | -13bp/+2bp 2/5 |
|  |  | TGATGTATTCCCAAAACACAACCATTACAGTTGAGACAAGCAGCAT-----------------------------------------------------G | -53bp 3/5 |
|  | P03 | TGATGTATTCCCAAAACACAACCATTACAGTTGAGACAAGCAGCATTGACAGAACCACTCTTCCTTTGACAAC-------------CCTGGGAAAAGAAAAG | -13bp/+2bp 3/8 |
|  |  | TGATGTATTCCCAAAACACAACCATTACAGTTGAGACAAGCAGCAT-----------------------------------------------------G | -53bp 5/8 |
|  | P04 | TGATGTATTCCCAAAACACAACCATTACAGTTGAGACAAGCAGCATTGACAGAACCACTCTTCCTTTGACAAC-------------CCTGGGAAAAGAAAAG | -13bp/+2bp 3/7 |
|  |  | TGATGTATTCCCAAAACACAACCATTACAGTTGAGACAAGCAGCAT-----------------------------------------------------G | -53bp 4/7 |
| CMAH | WT | TCACTGTCTTCCAACAGATCACGTACCTTACTCACG(84)TACTACACGAGCCTCCATCTGATTGG(62)GTAAGGAAGGGTGAGCCCTCAACTCCGAAGA | WT |
|  | P01 | TCACTGTCTT--------------------------**(84)**-------------------TGATTGG(62)GTAAGGAAGGGTGAGCCCTCAACTCCGAAGA | -123bp 6/15 |
|  |  | TCACTGTCTTCCAACAGATCACGTACCTTACTCACG(84)TACTACACGAGCCTCCA---------**(62)**---------------------ACTCCGAAGA | -102bp 9/15 |
|  | P02 | TCACTGTCTT--------------------------**(84)**-------------------TGATTGG(62)GTAAGGAAGGGTGAGCCCTCAACTCCGAAGA | -123bp 3/8 |
|  |  | TCACTGTCTTCCAACAGATCACGTACCTTACTCACG(84)TACTACACGAGCCTCCA---------**(62)**---------------------ACTCCGAAGA | -102bp 5/8 |
|  | P03 | TCACTGTCTT--------------------------**(84)**-------------------TGATTGG(62)GTAAGGAAGGGTGAGCCCTCAACTCCGAAGA | -123bp 1/6 |
|  |  | TCACTGTCTTCCAACAGATCACGTACCTTACTCACG(84)TACTACACGAGCCTCCA---------**(62)**---------------------ACTCCGAAGA | -102bp 5/6 |
|  | P04 | TCACTGTCTT--------------------------**(84)**-------------------TGATTGG(62)GTAAGGAAGGGTGAGCCCTCAACTCCGAAGA | -123bp 3/7 |
|  |  | TCACTGTCTTCCAACAGATCACGTACCTTACTCACG(84)TACTACACGAGCCTCCA---------**(62)**---------------------ACTCCGAAGA | -102bp 4/7 |
| β4GalNT2 | WT | AGCCCTAGATGTCTGTCGATCCTCAAGAT(99)CACCCTGGATGCGCAGACGCTGAAGCTTCTACC(15)CTACGGTGAAAACGGGTGAGATGGCAA | WT |
|  |  | AGCCCTAGATGTCTGTCGATCCTCAAGAT(99)CACCCTGGATGCGCAGACGCTGAAGCTTCTACC(27)CTACAGTGGAATCTGGTGAGATGGCAA | WT |
|  | P01 | AGCCCTAGATG--------------------**(99)**--------------------------------**(15)**-----------------AGATGGCAA | -183bp 2/4 |
|  |  | AGCCCTAGATG------------------**(99)**----------------------AAGCTTCTACC(27)CTACAGTGGAATCTGGTGAGATGGCAA | -139bp 2/4 |
|  | P02 | AGCCCTAGATG--------------------**(99)**--------------------------------**(15)**-----------------AGATGGCAA | -183bp 4/13 |
|  |  | AGCCCTAGATG------------------**(99)**----------------------AAGCTTCTACC(27)CTACAGTGGAATCTGGTGAGATGGCAA | -139bp 9/13 |
|  | P03 | AGCCCTAGATG--------------------**(99)**--------------------------------**(15)**-----------------AGATGGCAA | -183bp 12/18 |
|  |  | AGCCCTAGATG------------------**(99)**----------------------AAGCTTCTACC(27)CTACAGTGGAATCTGGTGAGATGGCAA | -139bp 6/18 |
|  | P04 | AGCCCTAGATG--------------------**(99)**--------------------------------**(15)**-----------------AGATGGCAA | -183bp 3/9 |
|  |  | AGCCCTAGATG------------------**(99)**----------------------AAGCTTCTACC(27)CTACAGTGGAATCTGGTGAGATGGCAA | -139bp 6/9 |

Note：P01, 8-GEC3P01; P02, 8-GEC3P02; P03, 8-GEC3P03; P04, 8-GEC3P04

**Table S10. Genotyping of colonies for GGTA1 gene by Sanger sequencing in the second gene editing**

| **Gene** | **No. of colonies** | **Sequence** | **Mutation** |
| --- | --- | --- | --- |
| GGTA1  on exon 8 | WT | ATGATGCGCATGAAGACCATCGGGGAGCACATCCTGGC(90)GGTGGCTCAGCTACAGGCCTGGTGGTACAAGGCACATCCTGACGAG | WT |
|  | C1# | ATGATGCGCATGAAGACCATCGGGGAGCACATCCTGGC(90)GGTGGCTCAGCTACAGGCCTGGTGGTACAAGGCACATCCTGACGAG | WT 3/10 |
|  |  | ATGATGCGCATCA---------------------------**(90)**----------------------------------------TGACGAG | +2bp-156bp 6/10 |
|  |  | ATGATGCGCATGAAGACCATCGGGGAGCACATCCTGGC(90)GGTGGCTCAGCTACAGGCCTGGTGG-ACAAGGCACATCCTGACGAG | -1bp 1/10 |
|  | C3# | ----------------------GTGGAGACCCTGGGCCAGTCGGTGGCTCAGCTACAGGCCTGGTGG-------CACATCCTGACGAG | -469bp/-7bp 12/17 |
|  |  | ATGAT-----------------------------------**(90)**-------------------------------GCACATCCTGACGAG | -154bp 5/17 |
|  | C4# | ATGATGCGCATGAAGACCATCGGGGAGCACATCCTGGC(90)GGTGGCTCAGCTACAGGCCTGGTGGTACAAGGCACATCCTGACGAG | WT 4/8 |
|  |  | ATGATGCGCATGAAGACCAT------------------**(90)**--------------------------ACAAGGCACATCCTGACGAG | -134bp 3/8 |
|  |  | ATGATGCGCATGAAGACCATCGGGGAGCACATCCTGGC(90)GGTGGCTCAGCTACAGGCCTGGTGGTCTACAAGGCACATCCTGACGAG | +2bp 1/8 |
|  | C6# | ATGATGCGCATGAAGACCATCGGGGAGCACATCCTGGC(90)GGTGGCTCAGCTACAGGCCTGGTGGTACAAGGCACATCCTGACGAG | WT 1/7 |
|  |  | ATGATGCGCATGAAGACCAT------------------(90)--------------------------ACAAGGCACATCCTGACGAG | -134bp 4/7 |
|  |  | ATGATGCGCATGAAGACCATCGGGGAGCACATCCTGGC**(90)**GGTGGCTCAGCTACAGGCCTGGTGGTTACAAGGCACATCCTGACGAG | +1bp 2/7 |
|  | C7# | ATGATGCGCATGAAGACCATCGGGGAGCACATCCTGGC(90)GGTGGCTCAGCTACAGGCCTGGTGGTACAAGGCACATCCTGACGAG | WT 3/6 |
|  |  | ATGATGCGCATGAAGACCAT------------------**(90)**--------------------------ACAAGGCACATCCTGACGAG | -134bp 2/6 |
|  |  | ATGATGCGCATGAAGACCATCGGGGAGCACATCCTGGC**(90)**GGTGGCTCAGCTACAGGCCTGGTGGTTACAAGGCACATCCTGACGAG | +1bp 1/6 |
|  |  | ATGATGCGCATGAAG-----------------------**(90)**-------------------------TACAAGGCACATCCTGACGAG | -138bp 12/16 |
|  | C12# | ATGATGCGCATGAAGACCAT------------------**(90)**--------------------------ACAAGGCACATCCTGACGAG | -134bp 1/16 |
|  |  | ATGATGCGC---------------------ATCCTGGC(90)GGTGGCTCAGCTACAGGCCTGGTGGTATACAAGGCACATCCTGACGAG | +2bp/-21bp 3/16 |
|  | C15# | ATGATGCGCATGAAGACCAT------------------**(90)**--------------------------ACAAGGCACATCCTGACGAG | -134bp 6/11 |
|  |  | ATGATGCGCATGAAGACCAATCGGGGAGCACATCCTGGC(90)GGTGGCTCAGCTACAGGCCTGGTGGTTACAAGGCACATCCTGACGAG | +2bp 5/11 |
|  | C16# | ATGATGCGCATGAAGACCATCGGGGAGCACATCCTGGC(90)GGTGGCTCAGCTACAGGCCTGGTGGTACAAGGCACATCCTGACGAG | WT 2/8 |
|  |  | ATGATGCGCATCA-------------------------**(90)**---------------------------------------TGACGAG | -154bp 1/8 |
|  |  | ATGATGCGCATGAAGACCA----------CATCCTGGC(90)GGTGGCTCAGCTACAGGCC---------------CATCCTGACGAG | -25bp 4/8 |
|  |  | ATGATGCGCATGAAGACCAATCGGGGAGCACATCCTGGC(90)GGTGGCTCAGCTACAGGCCTGGTGGTTACAAGGCACATCCTGACGAG | +2bp 1/8 |
|  | C19# | ATGATGCGCATGAAGACCATCGGGGAGCACATCCTGGC(90)GGTGGCTCAGCTACAGGCCTGGTGGTACAAGGCACATCCTGACGAG | WT 1/9 |
|  |  | ATGATGCGCATGAAGACCAT------------------**(90)**--------------------------ACAAGGCACATCCTGACGAG | -134bp 8/9 |
|  | C22# | ATGATGCGCATGAAGACCATCGGGGAGCACATCCTGGC(90)GGTGGCTCAGCTACAGGCCTGGTGGTACAAGGCACATCCTGACGAG | WT 5/9 |
|  |  | ATGATGCGCATGAAGACCA-------------------**(90)**---------------------------CAAGGCACATCCTGACGAG | -136bp 4/9 |
|  | C23# | ATGATGCGCATGAAGACCATCGGGGAGCACATCCTGGC(90)GGTGGCTCAGCTACAGGCCTGGTGGTACAAGGCACATCCTGACGAG | WT 1/9 |
|  |  | -----------------------------CATCCTGGC(90)GGTGGCTCAGCTACAGGCCTGGTGGTACAAGGCACATCCTGACGAG | -347bp 4/6 |
|  |  | ATGATGCGCATGAAGACC--------------------**(90)**-------------------------TACAAGGCACATCCTGACGAG | -135bp 1/6 |
|  | C28# | ATGATGCGCATGAAGACCATCGGGGAGCACATCCTGGC(90)GGTGGCTCAGCTACAGGCCTGGTGGTACAAGGCACATCCTGACGAG | WT 1/9 |
|  |  | ATGATGCGCATGAAGACC--------------------**(90)**-------------------------TACAAGGCACATCCTGACGAG | -135bp 3/6 |
|  |  | ATGATGCGCATGAAGAC---------------------**(90)**---------------------------------ACATCCTGACGAG | -144bp 2/6 |
|  | C31# | ATGATGCGCATGAAGACCATCGGGGAGCACATCCTGGC(90)GGTGGCTCAGCTACAGGCCTGGTGGTACAAGGCACATCCTGACGAG | WT 5/7 |
|  |  | ATGATGCGCATGAAGACC--------------------**(90)**-------------------------TACAAGGCACATCCTGACGAG | -135bp 2/7 |
|  | C36# | ATGATGCGCATGAAGACCATCGGGGAGCACATCCTGGC(90)GGTGGCTCAGCTACAGGCCTGGTGGTACAAGGCACATCCTGACGAG | WT 3/4 |
|  |  | ATGATGCGCATGAAGACC--------------------**(90)**-------------------------TACAAGGCACATCCTGACGAG | -135bp 1/4 |
|  | C54# | ATGATGCGCATGAAGACCATCGGGGAGCACATCCTGGC(90)GGTGGCTCAGCTACAGGCCTGGTGGTACAAGGCACATCCTGACGAG | WT 1/8 |
|  |  | ATGATGCGCATGAAGAC--TCGGGGAGCACATCCTGGC(90)GGTGGCTCAGCTACAGGCCTGGTGGTTACAAGGCACATCCTGACGAG | -135bp 1/8 |
|  |  | ATGATGCGCATGAAGACCAT------------------**(90)**--------------------------ACAAGGCACATCCTGACGAG | WT 2/8 |
|  |  | ATGATGCGCATGA-------------------------(90)-------------------------------GCACATCCTGACGAG | -135bp 1/8 |
|  |  | ATGATGCGCAT---------------------------**(90)**---------------------------------------------- | WT 3/8 |
|  | C58# | --------------------------------------**(90)**----------------------------------CATCCTGACGAG  GTAGCTGAGCCACCGACTGGCCCAGGGTCTCCACCCCAAAGTTGTTTTGGAAGACCTGATCCACGTCCATGCAGAAGAGGAAGTCCACCTCGTCTTCATGCGCAT (inserted sequence) | -440bp/+105bp 2/6 |
|  |  | ATGATGCGCATGAA------CGGGGAGCACATCCTGGC**(90)**GGTGGCTCA---------------------------TCCTGACGAG | -135bp 4/6 |

**Table S11. Genotyping the GGTA1, CMAH and β4GalNT2 genes of 10-GEC pigs by Sanger sequencing**

| **Gene** | **No. of pigs** | **Sequence** | **Mutation** |
| --- | --- | --- | --- |
| GGTA1  on exon 3 | WT | TGATGTATTCCCAAAACACAACCATTACAGTTGAGACAAGCAGCATTGACAGAACCACTCTTCCTTTGACATTCATTATTTTCTCCTGGGAAAAGAAAAG | WT |
|  | C3F01 | TGATGTATTCCCAAAACACAACCATTACAGTTGAGACAAGCAGCATTGACAGAACCACTCTTCCTTTGACAAC-------------CCTGGGAAAAGAAAAG | -13bp/+2bp 5/8 |
|  |  | TGATGTATTCCCAAAACACAACCATTACAGTTGAGACAAGCAGCAT-----------------------------------------------------G | -53bp 3/8 |
|  | C3F02 | TGATGTATTCCCAAAACACAACCATTACAGTTGAGACAAGCAGCATTGACAGAACCACTCTTCCTTTGACAAC-------------CCTGGGAAAAGAAAAG | -13bp/+2bp 1/9 |
|  |  | TGATGTATTCCCAAAACACAACCATTACAGTTGAGACAAGCAGCAT-----------------------------------------------------G | -53bp 8/9 |
|  | C3F03 | TGATGTATTCCCAAAACACAACCATTACAGTTGAGACAAGCAGCATTGACAGAACCACTCTTCCTTTGACAAC-------------CCTGGGAAAAGAAAAG | -13bp/+2bp 4/10 |
|  |  | TGATGTATTCCCAAAACACAACCATTACAGTTGAGACAAGCAGCAT-----------------------------------------------------G | -53bp 6/10 |
|  | C3F04 | TGATGTATTCCCAAAACACAACCATTACAGTTGAGACAAGCAGCATTGACAGAACCACTCTTCCTTTGACAAC-------------CCTGGGAAAAGAAAAG | -13bp/+2bp 2/9 |
|  |  | TGATGTATTCCCAAAACACAACCATTACAGTTGAGACAAGCAGCAT-----------------------------------------------------G | -53bp 7/9 |
|  | C3F05 | TGATGTATTCCCAAAACACAACCATTACAGTTGAGACAAGCAGCATTGACAGAACCACTCTTCCTTTGACAAC-------------CCTGGGAAAAGAAAAG | -13bp/+2bp 4/8 |
|  |  | TGATGTATTCCCAAAACACAACCATTACAGTTGAGACAAGCAGCAT-----------------------------------------------------G | -53bp 4/8 |
| GGTA1  on exon 8 | WT | ATGATGCGCATGAAGACCATCGGGGAGCACATCCTGGC(90)GGTGGCTCAGCTACAGGCCTGGTGGTACAAGGCACATCCTGACGAG |  |
|  | C3F01 | -----------------------GTGGAGACCCTGGGCCAGTCGGTGGCTCAGCTACAGGCCTGGTGG-------CACATCCTGACGAG | -469/-7bp 2/5 |
|  |  | ATGAT--------------------------------------**(90)**------------------------------------GCACATCCTGACGAG | -154bp 3/5 |
|  | C3F02 | -----------------------GTGGAGACCCTGGGCCAGTCGGTGGCTCAGCTACAGGCCTGGTGG-------CACATCCTGACGAG | -469/-7bp 5/7 |
|  |  | ATGAT--------------------------------------**(90)**------------------------------------GCACATCCTGACGAG | -154bp 2/7 |
|  | C3F03 | -----------------------GTGGAGACCCTGGGCCAGTCGGTGGCTCAGCTACAGGCCTGGTGG-------CACATCCTGACGAG | -469/-7bp 2/5 |
|  |  | ATGAT--------------------------------------**(90)**------------------------------------GCACATCCTGACGAG | -154bp 5/6 |
|  | C3F04 | -----------------------GTGGAGACCCTGGGCCAGTCGGTGGCTCAGCTACAGGCCTGGTGG-------CACATCCTGACGAG | -469/-7bp 9/9 |
|  | C3F05 | -----------------------GTGGAGACCCTGGGCCAGTCGGTGGCTCAGCTACAGGCCTGGTGG-------CACATCCTGACGAG | -469/-7bp 5/9 |
|  |  | ATGAT--------------------------------------**(90)**------------------------------------GCACATCCTGACGAG | -154bp 4/9 |
| CMAH | WT | TCACTGTCTTCCAACAGATCACGTACCTTACTCACG(84)TACTACACGAGCCTCCATCTGATTGG(62)GTAAGGAAGGGTGAGCCCTCAACTCCGAAGA |  |
|  | C3F01 | TCACTGTCTT--------------------------**(84)**-------------------TGATTGG(62)GTAAGGAAGGGTGAGCCCTCAACTCCGAAGA | -123bp 6/10 |
|  |  | TCACTGTCTTCCAACA----------CTTACTCACG(84)TACTACACGAGCCTCCA---------**(62)**---------------------ACTCCGAAGA | -102bp 4/10 |
|  | C3F02 | TCACTGTCTT--------------------------**(84)**-------------------TGATTGG(62)GTAAGGAAGGGTGAGCCCTCAACTCCGAAGA | -123bp 7/11 |
|  |  | TCACTGTCTTCCAACA----------CTTACTCACG(84)TACTACACGAGCCTCCA---------**(62)**---------------------ACTCCGAAGA | -102bp 4/11 |
|  | C3F03 | TCACTGTCTT--------------------------**(84)**-------------------TGATTGG(62)GTAAGGAAGGGTGAGCCCTCAACTCCGAAGA | -123bp 5/10 |
|  |  | TCACTGTCTTCCAACA----------CTTACTCACG(84)TACTACACGAGCCTCCA---------**(62)**---------------------ACTCCGAAGA | -102bp 5/10 |
|  | C3F04 | TCACTGTCTT--------------------------**(84)**-------------------TGATTGG(62)GTAAGGAAGGGTGAGCCCTCAACTCCGAAGA | -123bp 4/10 |
|  |  | TCACTGTCTTCCAACA----------CTTACTCACG(84)TACTACACGAGCCTCCA---------**(62)**---------------------ACTCCGAAGA | -102bp 6/10 |
|  | C3F05 | TCACTGTCTT--------------------------**(84)**-------------------TGATTGG(62)GTAAGGAAGGGTGAGCCCTCAACTCCGAAGA | -123bp 6/10 |
|  |  | TCACTGTCTTCCAACA----------CTTACTCACG(84)TACTACACGAGCCTCCA---------**(62)**---------------------ACTCCGAAGA | -102bp 4/10 |
| β4GalNT2 | WT | AGCCCTAGATGTCTGTCGATCCTCAAGAT(99)CACCCTGGATGCGCAGACGCTGAAGCTTCTACC(15)CTACGGTGAAAACGGGTGAGATGGCAA | WT |
|  |  | AGCCCTAGATGTCTGTCGATCCTCAAGAT(99)CACCCTGGATGCGCAGACGCTGAAGCTTCTACC(27)CTACAGTGGAATCTGGTGAGATGGCAA | WT |
|  | C3F01 | AGCCCTAGATG--------------------**(99)**--------------------------------**(15)**-----------------AGATGGCAA | -183bp 5/9 |
|  |  | AGCCCTAGATG------------------**(99)**----------------------AAGCTTCTACC(27)CTACAGTGGAATCTGGTGAGATGGCAA | -139bp 4/9 |
|  | C3F02 | AGCCCTAGATG--------------------**(99)**--------------------------------**(15)**-----------------AGATGGCAA | -183bp 6/10 |
|  |  | AGCCCTAGATG------------------**(99)**----------------------AAGCTTCTACC(27)CTACAGTGGAATCTGGTGAGATGGCAA | -139bp 4/10 |
|  | C3F03 | AGCCCTAGATG--------------------**(99)**--------------------------------**(15)**-----------------AGATGGCAA | -183bp 4/10 |
|  |  | AGCCCTAGATG------------------**(99)**----------------------AAGCTTCTACC(27)CTACAGTGGAATCTGGTGAGATGGCAA | -139bp 6/10 |
|  | C3F04 | AGCCCTAGATG--------------------**(99)**--------------------------------**(15)**-----------------AGATGGCAA | -183bp 5/10 |
|  |  | AGCCCTAGATG------------------**(99)**----------------------AAGCTTCTACC(27)CTACAGTGGAATCTGGTGAGATGGCAA | -139bp 5/10 |
|  | C3F05 | AGCCCTAGATG--------------------**(99)**--------------------------------**(15)**-----------------AGATGGCAA | -183bp 3/10 |
|  |  | AGCCCTAGATG------------------**(99)**----------------------AAGCTTCTACC(27)CTACAGTGGAATCTGGTGAGATGGCAA | -139bp 8/10 |

**Table S13.** Genotyping the GGTA1 gene of 10-GEC pigs by Sanger sequencing.

| **Gene** | **No. of pigs** | **Sequence** | **Mutation** |
| --- | --- | --- | --- |
| GGTA1  on exon 8 | WT | ATGATGCGCATGAAGACCATCGGGGAGCACATCCTGGC(90)GGTGGCTCAGCTACAGGCCTGGTGGTACAAGGCACATCCTGACGAG | WT |
|  | F01P05 | -----------------------GTGGAGACCCTGGGCCAGTCGGTGGCTCAGCTACAGGCCTGGTGG-------CACATCCTGACGAG | -469/-7bp 3/6 |
|  |  | ATGAT--------------------------------------**(90)**------------------------------------GCACATCCTGACGAG | -154bp 3/6 |
|  | F01P06 | -----------------------GTGGAGACCCTGGGCCAGTCGGTGGCTCAGCTACAGGCCTGGTGG-------CACATCCTGACGAG | -469/-7bp 6/8 |
|  |  | ATGAT--------------------------------------**(90)**------------------------------------GCACATCCTGACGAG | -154bp 2/8 |
|  | F01P16 | -----------------------GTGGAGACCCTGGGCCAGTCGGTGGCTCAGCTACAGGCCTGGTGG-------CACATCCTGACGAG | -469/-7bp 5/6 |
|  |  | ATGAT--------------------------------------**(90)**------------------------------------GCACATCCTGACGAG | -154bp 1/6 |
|  | F03P11 | -----------------------GTGGAGACCCTGGGCCAGTCGGTGGCTCAGCTACAGGCCTGGTGG-------CACATCCTGACGAG | -469/-7bp 7/7 |
|  | F04P01 | -----------------------GTGGAGACCCTGGGCCAGTCGGTGGCTCAGCTACAGGCCTGGTGG-------CACATCCTGACGAG | -469/-7bp 5/8 |
|  |  | ATGAT--------------------------------------**(90)**------------------------------------GCACATCCTGACGAG | -154bp 3/8 |
|  | F04P02 | -----------------------GTGGAGACCCTGGGCCAGTCGGTGGCTCAGCTACAGGCCTGGTGG-------CACATCCTGACGAG | -469/-7bp 4/4 |
|  | F04P13 | -----------------------GTGGAGACCCTGGGCCAGTCGGTGGCTCAGCTACAGGCCTGGTGG-------CACATCCTGACGAG | -469/-7bp 7/7 |

Note: F01P05, 10-GEC3F01P05; F01P06, 10-GEC3F01P06; P04, 10-GEC3F01P16; F03P11, 10-GEC3F03P11; F04P01, 10-GEC3F04P01; P07, 10-GEC3F04P02; F04P13, 10-GEC3F04P13

| **Table S15. Annotation information of transgenic insertion location** | | | | | | | | |
| --- | --- | --- | --- | --- | --- | --- | --- | --- |
| **Chromosome** | **Left side of insertion position** | **Right side of insertion position** | **Strand** | **10-GEC3F04P16P05** | **10-GEC3F04P16P10** | **10-GEC3F04P12** | **Location information** | **Annotation of location** |
| **Chr5** | 63169557 | 63169558 | + | √ | √ | √ | Intron region of loc110260747 gene | exon2:63159164-63159277 intron:63159278-63180148 exon3:63180149-63180300 |
| **Chr6** | 123097526 | 123097527 | + | √ | √ | √ | Intergenic region | Without gene between 10MB upstream and downstream |
| **Chr13** | 139800856 | 139800857 | - | √ | √ | √ | Intergenic region | Without gene between 10MB upstream and downstream |
| Note：“√” refers to at least 2 reads that support the insertion sequence at the corresponding locus. | | | | | | | | |

| **Table S16. The F1 generation of 10-GEC male (♂) pigs naturally mated with 8-GEC female (♀) pigs** | | | | | | |
| --- | --- | --- | --- | --- | --- | --- |
| No. | ID | Parents | Body weight (g) | Status at birth | Survival time (day) | Gender |
| 1 | G1F1P01 | DN10-GEC3F02P02×  DN8TGC12F01P17 | 550 | weak | 2 | ♂ |
| 2 | G1F1P02 |  | 580 | healthy | 58 | ♀ |
| 3 | G1F1P03 |  | 510 | weak | 3 | ♀ |
| 4 | G1F1P04 | DN10-GEC3F02P02  or  DN10-GEC3F03P07  ×  DN8TGC12F01P08 | 260 | weak | 1 | ♂ |
| 5 | G1F1P05 |  | 430 | healthy | - | ♂ |
| 6 | G1F1P06 |  | 320 | stillbirth | 0 | ♂ |
| 7 | G1F1P07 |  | 290 | weak | 56 | ♂ |
| 8 | G1F1P08 |  | 400 | healthy | - | ♀ |
| 9 | G1F1P09 |  | 420 | healthy | - | ♂ |
| 10 | G1F1P10 |  | 380 | stillbirth | 0 | ♂ |
| 11 | G1F1P11 |  | 410 | healthy | - | ♀ |
| 12 | G1F1P12 | DN10-GEC3F01P04  ×  DN8TGC12F01P02 | 630 | healthy | - | ♀ |
| 13 | G1F1P13 |  | 610 | healthy | 11 | ♀ |
| 14 | G1F1P14 |  | 550 | healthy | - | ♀ |
| 15 | G1F1P15 |  | 550 | healthy | - | ♂ |
| 16 | G1F1P16 |  | 480 | healthy | - | ♀ |
| 17 | G1F1P17 |  | 640 | healthy | - | ♂ |
| 18 | G1F1P18 |  | 400 | healthy | - | ♂ |
| 19 | G1F1P19 |  | 350 | healthy | 11 | ♂ |
| 20 | G1F1P20 | DN10-GEC3F02P02  or  DN10-GEC3F04P12  ×  DN8TGC12F01P25 | 550 | healthy | - | ♀ |
| 21 | G1F1P21 |  | 380 | healthy | - | ♂ |
| 22 | G1F1P22 |  | 320 | stillbirth | 0 | ♂ |
| 23 | G1F1P23 |  | 540 | healthy | - | ♀ |
| 24 | G1F1P24 |  | 230 | stillbirth | 0 | ♂ |

**Table S17.** **Genotype of 3 xenoantigens in F1 generation of 10-GEC pigs by Sanger sequencing**

| **Gene** | **No. of pigs** | **Sequence** | **Mutation** |
| --- | --- | --- | --- |
| GGTA1  on exon 3 | WT | TGATGTATTCCCAAAACACAACCATTACAGTTGAGACAAGCAGCATTGACAGAACCACTCTTCCTTTGACATTCATTATTTTCTCCTGGGAAAAGAAAAG | WT |
|  | G1F1P01 | TGATGTATTCCCAAAA-----------------------------------------------------ACATTCATTATTTTCTCCTGGGAAAAGAAAAG | -52bp 3/10 |
|  |  | TGATGTATTCCCAAAACACAACCATTACAGTTGAGACAAGCAGCAT-----------------------------------------------------G | -53bp 7/10 |
|  | G1F1P02 | TGATGTATTCCCAAAA-----------------------------------------------------ACATTCATTATTTTCTCCTGGGAAAAGAAAAG | -52bp 5/10 |
|  |  | TGATGTATTCCCAAAACACAACCATTACAGTTGAGACAAGCAGCATTGACAGAACCACTCTTCCTTTGACAAC-------------CCTGGGAAAAGAAAAG | +2/-13bp 5/10 |
|  | G1F1P03 | TGATGTATTCCCAAAA-----------------------------------------------------ACATTCATTATTTTCTCCTGGGAAAAGAAAAG | -52bp 4/9 |
|  |  | TGATGTATTCCCAAAACACAACCATTACAGTTGAGACAAGCAGCATTGACAGAACCACTCTTCCTTTGACAAC-------------CCTGGGAAAAGAAAAG | +2/-13bp 5/9 |
|  | G1F1P04 | TGATGTATTCCCAAAA-----------------------------------------------------ACATTCATTATTTTCTCCTGGGAAAAGAAAAG | -52bp 2/9 |
|  |  | TGATGTATTCCCAAAACACAACCATTACAGTTGAGACAAGCAGCATTGACAGAACCACTCTTCCTTTGACAAC-------------CCTGGGAAAAGAAAAG | +2/-13bp 7/9 |
|  | G1F1P05 | TGATGTATTCCCAAAA-----------------------------------------------------ACATTCATTATTTTCTCCTGGGAAAAGAAAAG | -52bp 4/9 |
|  |  | TGATGTATTCCCAAAACACAACCATTACAGTTGAGACAAGCAGCATTGACAGAACCACTCTTCCTTTGACAAC-------------CCTGGGAAAAGAAAAG | +2/-13bp 5/9 |
|  | G1F1P06 | TGATGTATTCCCAAAA-----------------------------------------------------ACATTCATTATTTTCTCCTGGGAAAAGAAAAG | -52bp 5/9 |
|  |  | TGATGTATTCCCAAAACACAACCATTACAGTTGAGACAAGCAGCATTGACAGAACCACTCTTCCTTTGACAAC-------------CCTGGGAAAAGAAAAG | +2/-13bp 4/9 |
|  | G1F1P07 | TGATGTATTCCCAAAA-----------------------------------------------------ACATTCATTATTTTCTCCTGGGAAAAGAAAAG | -52bp 5/10 |
|  |  | TGATGTATTCCCAAAACACAACCATTACAGTTGAGACAAGCAGCATTGACAGAACCACTCTTCCTTTGACAAC-------------CCTGGGAAAAGAAAAG | +2/-13bp 5/10 |
|  | G1F1P08 | TGATGTATTCCCAAAA-----------------------------------------------------ACATTCATTATTTTCTCCTGGGAAAAGAAAAG | -52bp 2/10 |
|  |  | TGATGTATTCCCAAAACACAACCATTACAGTTGAGACAAGCAGCAT-----------------------------------------------------G | -53bp 8/10 |
|  | G1F1P09 | TGATGTATTCCCAAAA-----------------------------------------------------ACATTCATTATTTTCTCCTGGGAAAAGAAAAG | -52bp 3/9 |
|  |  | TGATGTATTCCCAAAACACAACCATTACAGTTGAGACAAGCAGCAT-----------------------------------------------------G | -53bp 6/9 |
|  | G1F1P10 | TGATGTATTCCCAAAA-----------------------------------------------------ACATTCATTATTTTCTCCTGGGAAAAGAAAAG | -52bp 5/10 |
|  |  | TGATGTATTCCCAAAACACAACCATTACAGTTGAGACAAGCAGCATTGACAGAACCACTCTTCCTTTGACAAC-------------CCTGGGAAAAGAAAAG | +2/-13bp 5/10 |
|  | G1F1P11 | TGATGTATTCCCAAAA-----------------------------------------------------ACATTCATTATTTTCTCCTGGGAAAAGAAAAG | -52bp 7/10 |
|  |  | TGATGTATTCCCAAAACACAACCATTACAGTTGAGACAAGCAGCATTGACAGAACCACTCTTCCTTTGACAAC-------------CCTGGGAAAAGAAAAG | +2/-13bp 3/10 |
| GGTA1  on exon 8 | WT | ATGATGCGCATGAAGACCATCGGGGAGCACATCCTGGC(90)GGTGGCTCAGCTACAGGCCTGGTGGTACAAGGCACATCCTGACGAG | WT |
|  | G1F1P01 | ATGATGCGCATGAAGACCATCGGGGAGCACATCCTGGC(90)GGTGGCTCAGCTACAGGCCTGGTGGTACAAGGCACATCCTGACGAG | WT 1/7 |
|  |  | ATGAT--------------------------------------**(90)**------------------------------------GCACATCCTGACGAG | -154bp 6/7 |
|  | G1F1P02 | ATGATGCGCATGAAGACCATCGGGGAGCACATCCTGGC(90)GGTGGCTCAGCTACAGGCCTGGTGGTACAAGGCACATCCTGACGAG | WT 2/10 |
|  |  | -----------------------GTGGAGACCCTGGGCCAGTCGGTGGCTCAGCTACAGGCCTGGTGG-------CACATCCTGACGAG | -7/-469bp 8/10 |
|  | G1F1P03 | ATGATGCGCATGAAGACCATCGGGGAGCACATCCTGGC(90)GGTGGCTCAGCTACAGGCCTGGTGGTACAAGGCACATCCTGACGAG | WT 4/9 |
|  |  | -----------------------GTGGAGACCCTGGGCCAGTCGGTGGCTCAGCTACAGGCCTGGTGG-------CACATCCTGACGAG | -7/-469bp 5/9 |
|  | G1F1P04 | ATGATGCGCATGAAGACCATCGGGGAGCACATCCTGGC(90)GGTGGCTCAGCTACAGGCCTGGTGGTACAAGGCACATCCTGACGAG | WT 3/10 |
|  |  | -----------------------GTGGAGACCCTGGGCCAGTCGGTGGCTCAGCTACAGGCCTGGTGG-------CACATCCTGACGAG | -7/-469bp 7/10 |
|  | G1F1P05 | ATGATGCGCATGAAGACCATCGGGGAGCACATCCTGGC(90)GGTGGCTCAGCTACAGGCCTGGTGGTACAAGGCACATCCTGACGAG | WT 2/10 |
|  |  | -----------------------GTGGAGACCCTGGGCCAGTCGGTGGCTCAGCTACAGGCCTGGTGG-------CACATCCTGACGAG | -7/-469bp 8/10 |
|  | G1F1P06 | ATGATGCGCATGAAGACCATCGGGGAGCACATCCTGGC(90)GGTGGCTCAGCTACAGGCCTGGTGGTACAAGGCACATCCTGACGAG | WT 3/10 |
|  |  | -----------------------GTGGAGACCCTGGGCCAGTCGGTGGCTCAGCTACAGGCCTGGTGG-------CACATCCTGACGAG | -7/-469bp 7/10 |
|  | G1F1P07 | ATGATGCGCATGAAGACCATCGGGGAGCACATCCTGGC(90)GGTGGCTCAGCTACAGGCCTGGTGGTACAAGGCACATCCTGACGAG | WT 1/12 |
|  |  | -----------------------GTGGAGACCCTGGGCCAGTCGGTGGCTCAGCTACAGGCCTGGTGG-------CACATCCTGACGAG | -7/-469bp 11/12 |
|  | G1F1P08 | ATGATGCGCATGAAGACCATCGGGGAGCACATCCTGGC(90)GGTGGCTCAGCTACAGGCCTGGTGGTACAAGGCACATCCTGACGAG | WT 4/7 |
|  |  | -----------------------GTGGAGACCCTGGGCCAGTCGGTGGCTCAGCTACAGGCCTGGTGG-------CACATCCTGACGAG | -7/-469bp 3/7 |
|  | G1F1P09 | ATGATGCGCATGAAGACCATCGGGGAGCACATCCTGGC(90)GGTGGCTCAGCTACAGGCCTGGTGGTACAAGGCACATCCTGACGAG | WT 6/10 |
|  |  | -----------------------GTGGAGACCCTGGGCCAGTCGGTGGCTCAGCTACAGGCCTGGTGG-------CACATCCTGACGAG | -7/-469bp 4/10 |
|  | G1F1P10 | ATGATGCGCATGAAGACCATCGGGGAGCACATCCTGGC(90)GGTGGCTCAGCTACAGGCCTGGTGGTACAAGGCACATCCTGACGAG | WT 3/10 |
|  |  | -----------------------GTGGAGACCCTGGGCCAGTCGGTGGCTCAGCTACAGGCCTGGTGG-------CACATCCTGACGAG | -7/-469bp 7/10 |
|  | G1F1P11 | ATGATGCGCATGAAGACCATCGGGGAGCACATCCTGGC(90)GGTGGCTCAGCTACAGGCCTGGTGGTACAAGGCACATCCTGACGAG | WT 2/8 |
|  |  | -----------------------GTGGAGACCCTGGGCCAGTCGGTGGCTCAGCTACAGGCCTGGTGG-------CACATCCTGACGAG | -7/-469bp 6/8 |
| CMAH | WT | TCACTGTCTTCCAACAGATCACGTACCTTACTCACG(84)TACTACACGAGCCTCCATCTGATTGG(62)GTAAGGAAGGGTGAGCCCTCAACTCCGAAGA | WT |
|  | G1F1P01 | TCACTGTCTTCCAAC-GATCACGTACCTTACTCACG(84)TACTACACGAGCCTCCA---GATTGG(62)GTAAGGAAGGGTGAGCCCTCAACTCCGAAGA | -4 bp 4/8 |
|  |  | TCACTGTCTT--------------------------**(84)**-------------------TGATTGG(62)GTAAGGAAGGGTGAGCCCTCAACTCCGAAGA | -123bp 4/8 |
|  | G1F1P02 | TCACTGTCTTCCAAC-GATCACGTACCTTACTCACG(84)TACTACACGAGCCTCCA---GATTGG(62)GTAAGGAAGGGTGAGCCCTCAACTCCGAAGA | -4 bp 4/10 |
|  |  | TCACTGTCTT--------------------------**(84)**-------------------TGATTGG(62)GTAAGGAAGGGTGAGCCCTCAACTCCGAAGA | -123bp 6/10 |
|  | G1F1P03 | TCACTGTCTTCCAAC-GATCACGTACCTTACTCACG(84)TACTACACGAGCCTCCA---GATTGG(62)GTAAGGAAGGGTGAGCCCTCAACTCCGAAGA | -4 bp 4/10 |
|  |  | TCACTGTCTT--------------------------**(84)**-------------------TGATTGG(62)GTAAGGAAGGGTGAGCCCTCAACTCCGAAGA | -123bp 6/10 |
|  | G1F1P04 | TCACTGTCTTCCAAC-GATCACGTACCTTACTCACG(84)TACTACACGAGCCTCCA---GATTGG(62)GTAAGGAAGGGTGAGCCCTCAACTCCGAAGA | -4 bp 4/10 |
|  |  | TCACTGTCTT--------------------------**(84)**-------------------TGATTGG(62)GTAAGGAAGGGTGAGCCCTCAACTCCGAAGA | -123bp 6/10 |
|  | G1F1P05 | TCACTGTCTTCCAACAGAT-----------------**(84)**-----------------------TGG(62)GTAAGGAAGGGTGAGCCCTCAACTCCGAAGA | -124bp 4/10 |
|  |  | TCACTGTCTTCCAACA----------CTTACTCACG(84)TACTACACGAGCCTCCA---------**(62)**---------------------ACTCCGAAGA | -10/-92bp 6/10 |
|  | G1F1P06 | TCACTGTCTTCCAAC-GATCACGTACCTTACTCACG(84)TACTACACGAGCCTCCA---GATTGG(62)GTAAGGAAGGGTGAGCCCTCAACTCCGAAGA | -4 bp 4/8 |
|  |  | TCACTGTCTTCCAACA----------CTTACTCACG(84)TACTACACGAGCCTCCA---------**(62)**---------------------ACTCCGAAGA | -10/-92bp 4/8 |
|  | G1F1P07 | TCACTGTCTTCCAAC-GATCACGTACCTTACTCACG(84)TACTACACGAGCCTCCA---GATTGG(62)GTAAGGAAGGGTGAGCCCTCAACTCCGAAGA | -4 bp 2/10 |
|  |  | TCACTGTCTTCCAACA----------CTTACTCACG(84)TACTACACGAGCCTCCA---------**(62)**---------------------ACTCCGAAGA | -10/-92bp 8/10 |
|  | G1F1P08 | TCACTGTCTTCCAAC-GATCACGTACCTTACTCACG(84)TACTACACGAGCCTCCA---GATTGG(62)GTAAGGAAGGGTGAGCCCTCAACTCCGAAGA | -4 bp 4/9 |
|  |  | TCACTGTCTT--------------------------**(84)**-------------------TGATTGG(62)GTAAGGAAGGGTGAGCCCTCAACTCCGAAGA | -123bp 5/9 |
|  | G1F1P09 | TCACTGTCTTCCAAC-GATCACGTACCTTACTCACG(84)TACTACACGAGCCTCCA---GATTGG(62)GTAAGGAAGGGTGAGCCCTCAACTCCGAAGA | -4 bp 5/10 |
|  |  | TCACTGTCTTCCAACA----------CTTACTCACG(84)TACTACACGAGCCTCCA---------**(62)**---------------------ACTCCGAAGA | -10/-92bp 5/10 |
|  | G1F1P10 | TCACTGTCTTCCAACAGAT-----------------**(84)**-----------------------TGG(62)GTAAGGAAGGGTGAGCCCTCAACTCCGAAGA | -124bp 3/10 |
|  |  | TCACTGTCTTCCAACA----------CTTACTCACG(84)TACTACACGAGCCTCCA---------**(62)**---------------------ACTCCGAAGA | -10/-92bp 7/10 |
|  | F1P11 | TCACTGTCTTCCAAC-GATCACGTACCTTACTCACG(84)TACTACACGAGCCTCCA---GATTGG(62)GTAAGGAAGGGTGAGCCCTCAACTCCGAAGA | -4bp 5/9 |
|  |  | TCACTGTCTT--------------------------**(84)**-------------------TGATTGG(62)GTAAGGAAGGGTGAGCCCTCAACTCCGAAGA | -123bp 4/9 |
|  |  | TCACTGTCTTCCAACAGAT-----------------**(84)**-----------------------TGG(62)GTAAGGAAGGGTGAGCCCTCAACTCCGAAGA | -124bp 6/7 |
| β4GalNT2 | WT | AGCCCTAGATGTCTGTCGATCCTCAAGAT(99)CACCCTGGATGCGCAGACGCTGAAGCTTCTACC(15)CTACGGTGAAAACGGGTGAGATGGCAA | WT |
|  |  | AGCCCTAGATGTCTGTCGATCCTCAAGAT(99)CACCCTGGATGCGCAGACGCTGAAGCTTCTACC(27)CTACAGTGGAATCTGGTGAGATGGCAA | WT |
|  | G1F1P01 | AGCCCTAGAG------------------**(99)**--------ATGCGCAGACGCTGAAGCTTCTACC(27)CTACAGTGGAATCTGGTGAGATGGCAA | -125bp 4/16 |
|  |  | AGCCCTAGAT--------------AAGAT(99)CACCCTGGAT-----------------------(15)------------------AGATGGCAA | -14/-56bp 4/16 |
|  |  | AGCCCTAGATG--------------------**(99)**--------------------------------**(15)**-----------------AGATGGCAA | -183bp 1/16 |
|  |  | AGCCCTAGATG------------------**(99)**----------------------AAGCTTCTACC(27)CTACAGTGGAATCTGGTGAGATGGCAA | -139bp 7/16 |
|  | G1F1P02 | AGCCCTAGAG------------------**(99)**--------ATGCGCAGACGCTGAAGCTTCTACC(27)CTACAGTGGAATCTGGTGAGATGGCAA | -125bp 6/16 |
|  |  | AGCCCTAGAT--------------AAGAT(99)CACCCTGGAT-----------------------(15)------------------AGATGGCAA | -14/-56bp 1/16 |
|  |  | AGCCCTAGATG--------------------**(99)**--------------------------------**(15)**-----------------AGATGGCAA | -183bp 5/16 |
|  |  | AGCCCTAGATG------------------**(99)**----------------------AAGCTTCTACC(27)CTACAGTGGAATCTGGTGAGATGGCAA | -139bp 4/16 |
|  | G1F1P03 | AGCCCTAGAT-------------------**(99)**---------TGCGCAGACGCTGAAGCTTCTACC(27)CTACAGTGGAATCTGGTGAGATGGCAA | -127bp 11/16 |
|  |  | AGCCCTAGATGTCTGTCGATCCTCAAGAT(99)CACCCTGGA------------------------(15)------------------AGATGGCAA | -57bp 5/16 |
|  | G1F1P04 | AGCCCTAGAG------------------**(99)**--------ATGCGCAGACGCTGAAGCTTCTACC(27)CTACAGTGGAATCTGGTGAGATGGCAA | -125bp 8/15 |
|  |  | AGCCCTAGAT--------------AAGAT(99)CACCCTGGAT-----------------------(15)------------------AGATGGCAA | -14/-56bp 3/15 |
|  |  | AGCCCTAGATG------------------**(99)**----------------------AAGCTTCTACC(27)CTACAGTGGAATCTGGTGAGATGGCAA | -139bp 4/15 |
|  | G1F1P05 | AGCCCTAGAG------------------**(99)**--------ATGCGCAGACGCTGAAGCTTCTACC(27)CTACAGTGGAATCTGGTGAGATGGCAA | -125bp 4/16 |
|  |  | AGCCCTAGAT--------------AAGAT(99)CACCCTGGAT-----------------------(15)------------------AGATGGCAA | -14/-56bp 5/16 |
|  |  | AGCCCTAGATG--------------------**(99)**--------------------------------**(15)**-----------------AGATGGCAA | -183bp 2/16 |
|  |  | AGCCCTAGATG------------------**(99)**----------------------AAGCTTCTACC(27)CTACAGTGGAATCTGGTGAGATGGCAA | -139bp 5/16 |
|  | G1F1P06 | AGCCCTAGAT-------------------**(99)**---------TGCGCAGACGCTGAAGCTTCTACC(27)CTACAGTGGAATCTGGTGAGATGGCAA | -127bp 4/16 |
|  |  | AGCCCTAGATGTCTGTCGATCCTCAAGAT(99)CACCCTGGA------------------------(15)------------------AGATGGCAA | -57bp 3/16 |
|  |  | AGCCCTAGATG--------------------**(99)**--------------------------------**(15)**-----------------AGATGGCAA | -183bp 3/16 |
|  |  | AGCCCTAGATG------------------**(99)**----------------------AAGCTTCTACC(27)CTACAGTGGAATCTGGTGAGATGGCAA | -139bp 6/16 |
|  | G1F1P07 | AGCCCTAGAG------------------**(99)**--------ATGCGCAGACGCTGAAGCTTCTACC(27)CTACAGTGGAATCTGGTGAGATGGCAA | -125bp 14/16 |
|  |  | AGCCCTAGAT--------------AAGAT(99)CACCCTGGAT-----------------------(15)------------------AGATGGCAA | -14/-56bp 2/16 |
|  | G1F1P08 | AGCCCTAGAG------------------**(99)**--------ATGCGCAGACGCTGAAGCTTCTACC(27)CTACAGTGGAATCTGGTGAGATGGCAA | -125bp 5/14 |
|  |  | AGCCCTAGATG--------------------**(99)**--------------------------------**(15)**-----------------AGATGGCAA | -183bp 3/14 |
|  |  | AGCCCTAGATG------------------**(99)**----------------------AAGCTTCTACC(27)CTACAGTGGAATCTGGTGAGATGGCAA | -139bp 6/14 |
|  | G1F1P09 | AGCCCTAGAG------------------**(99)**--------ATGCGCAGACGCTGAAGCTTCTACC(27)CTACAGTGGAATCTGGTGAGATGGCAA | -125bp 11/15 |
|  |  | AGCCCTAGAT--------------AAGAT(99)CACCCTGGAT-----------------------(15)------------------AGATGGCAA | -14/-56bp 4/15 |
|  | G1F1P10 | AGCCCTAGAG------------------**(99)**--------ATGCGCAGACGCTGAAGCTTCTACC(27)CTACAGTGGAATCTGGTGAGATGGCAA | -125bp 11/15 |
|  |  | AGCCCTAGAT--------------AAGAT(99)CACCCTGGAT-----------------------(15)------------------AGATGGCAA | -14/-56bp 4/15 |
|  | G1F1P11 | AGCCCTAGAG------------------**(99)**--------ATGCGCAGACGCTGAAGCTTCTACC(27)CTACAGTGGAATCTGGTGAGATGGCAA | -125bp 10/16 |
|  |  | AGCCCTAGAT--------------AAGAT(99)CACCCTGGAT-----------------------(15)------------------AGATGGCAA | -14/-56bp 6/16 |

| **Table S18. Summary of the genotypes of F1 generation produced by breeding the 10-GEC male pigs with 8-GEC female pigs.** | | | | | | | | | | | | | |
| --- | --- | --- | --- | --- | --- | --- | --- | --- | --- | --- | --- | --- | --- |
| No. | Father | Mother | ID | GGTA1 | CMAH | β4GalNT2 | hCD46 | hCD55 | hCD59 | hTBM | hCD39 | hEPCR | hCD47 |
| 1 | DN10-GEC3F02P02 | DN8TGC12F01P17 | G1F1P01 | △52/△53  WT/△154 | △123/△1△3 | △183/△139/  △125/△14△56 | ○ | ○ | ○ | ○ | ○ | × | × |
| 2 |  |  | G1F1P02 | △52/+2△13  WT/△7△469 | △124/△102 | △183/△139/  △125/△14△56 | ○ | ○ | ○ | ○ | ○ | ○ | ○ |
| 3 |  |  | G1F1P03 | △52/+2△13  WT/△7△469 | △123/△1△3 | △125/△14△56 | ○ | ○ | ○ | ○ | ○ | ○ | ○ |
| 4 | DN10-GEC3F02P02  or  DN10-GEC3F03P07 | DN8TGC12F01P08 | G1F1P04 | △52/+2△13  WT/△7△469 | △124/△102 | △139/△183 | ○ | ○ | ○ | ○ | ○ | × | × |
| 5 |  |  | G1F1P05 | △52/+2△13  WT/△7△469 | △123/△1△3 | △183/△139/  △125/△14△56 | ○ | ○ | ○ | ○ | ○ | × | × |
| 6 |  |  | G1F1P06 | △52/+2△13  WT/△7△469 | △124/△102 | △183/△139/  △125/△14△56 | ○ | ○ | ○ | ○ | ○ | × | × |
| 7 |  |  | G1F1P07 | △52/+2△13  WT/△7△469 | △124/△102 | △125/△14△56 | ○ | ○ | ○ | ○ | ○ | × | × |
| 8 |  |  | G1F1P08 | △52/△53  WT/△154 | △123/△1△3 | △139/△125/  △14△56 | ○ | ○ | ○ | ○ | ○ | ○ | ○ |
| 9 |  |  | G1F1P09 | △52/△53  WT/△154 | △124/△102 | △125/△14△56 | ○ | ○ | ○ | ○ | ○ | ○ | ○ |
| 10 |  |  | G1F1P10 | △52/+2△13  WT/△7△469 | △124/△102 | △139/△183 | ○ | ○ | ○ | ○ | ○ | ○ | ○ |
| 11 |  |  | G1F1P11 | △52/+2△13  WT/△7△469 | △124/△102 | △125/△14△56 | ○ | ○ | ○ | ○ | ○ | × | × |
| Note: “○” refers to detectable transgene, “×” refers to undetectable transgene. | | | | | | | | | | | | | |

| **Table S20. Information of recipient** **Tibetan macaques and donor pigs** | | | | | | | |
| --- | --- | --- | --- | --- | --- | --- | --- |
| NO. | ID | Gender | Age (year) | Weight (Kg) | Blood type | Transplantation | Donor Information |
| 1 | 823787# | Male | 17 | 16.45 | A | Kidney | 33dDN10-GEC3F02P02 (34.1Kg, 10 month, Male, Blood type O) |
| 2 | 335362# | Male | 17 | 16.70 | AB | Liver |  |
| 3 | 3134985# | Male | 17 | 19.3 | A | Heart |  |
| 4 | 335346# | Male | 16 | 15.65 | A | Kidney | 33dDN10-GEC3F04P12 (32.4 Kg, 11 month, Male, Blood type O) |
